# Compact, hydrophilic, lanthanide-binding tags for paramagnetic NMR spectroscopy†
†Electronic supplementary information (ESI) available: Syntheses of **C5** and **C6**, ^1^H NMR spectra and HRMS of Yb^3+^ complexes of **C5–C7**, experimental details for expression, purification and tagging of GB1 Q32C and HPPK S112C/C80A, ^15^N-HSQC spectra of differently tagged ubiquitin A28C, GB1 Q32C and HPPK S112C/C80A, correlation between PCSs of **C7** tagged ubiquitin A28C at pH 8 and pH 6.5, comparison of measured PCSs against residue number for **C5–C8** tagged ubiquitin A28C, isosurface representations of Δ*χ*-tensors of **C1**, **C7** and **C8** tagged ubiquitin A28C, simultaneously calculated Δ*χ*-tensor properties for tagged ubiquitin A28C and GB1 Q32C, experimental RDCs and alignment tensor properties for **C7-Tm^3+^** and **C8-Tm^3+^** tagged ubiquitin A28C, experimental PCSs of tagged ubiquitin A28C, GB1 Q32C and HPPK S112C/C80A, 1D ^1^H and ^13^C NMR spectra of novel small molecule compounds, analytical HPLC traces of **C5–C7**. See DOI: 10.1039/c4sc03892d


**DOI:** 10.1039/c4sc03892d

**Published:** 2015-02-25

**Authors:** M. D. Lee, C.-T. Loh, J. Shin, S. Chhabra, M. L. Dennis, G. Otting, J. D. Swarbrick, B. Graham

**Affiliations:** a Monash Institute of Pharmaceutical Sciences , Monash University , Parkville , VIC 3052 , Australia . Email: bim.graham@monash.edu ; Email: james.swarbrick@monash.edu; b Research School of Chemistry , Australian National University , Canberra , ACT 0200 , Australia

## Abstract

The design, synthesis and evaluation of four novel lanthanide-binding tags for paramagnetic NMR spectroscopy are reported. Each tag is based on the ((2*S*,2′*S*,2′′*S*,2′′′*S*)-1,1′,1′′,1′′′-(1,4,7,10-tetraazacyclododecane-1,4,7,10-tetrayl)tetrakis(propan-2-ol)) scaffold, featuring small chiral alcohol coordinating pendants to minimise the size and hydrophobic character of each tag. The tags feature different linkers of variable length for conjugation to protein *via* a single cysteine residue. Each tag's ability to induce pseudocontact shifts (PCS) was assessed on a ubiquitin A28C mutant. Two enantiomeric tags of particular note, **C7** and **C8**, produced significantly larger Δ*χ*-tensors compared to a previously developed tag, **C1**, attributed to the extremely short linker utilised, limiting the mobility of the bound lanthanide ion. The **C7** and **C8** tags' capacity to induce PCSs was further demonstrated on GB1 Q32C and 6-hydroxymethyl-7,8-dihydropterin pyrophosphokinase (HPPK) S112C/C80A mutants. Whilst factors such as the choice of lanthanide ion, pH and site of conjugation influence the size of the PCSs obtained, the tags represent a significant advance in the field.

## Abstract 2

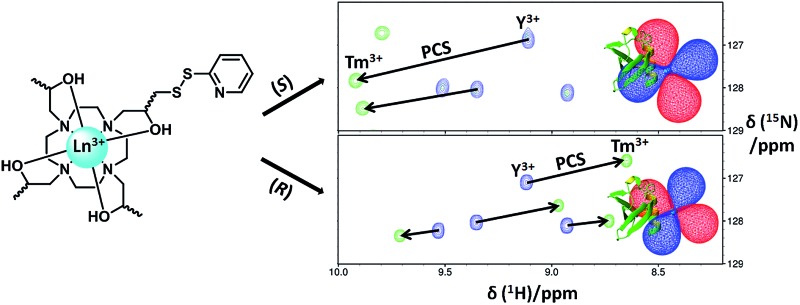
The design, synthesis and evaluation of four novel lanthanide-binding tags for paramagnetic NMR spectroscopy are reported.

## Introduction

The site-specific incorporation of paramagnetic metal ions into proteins allows access to unique NMR parameters that can provide valuable structural insights into protein structure and dynamics.^1–3^ These include pseudocontact shifts (PCS), residual dipolar couplings (RDC) and paramagnetic relaxation enhancement (PRE). PCSs are particularly attractive structural restraints as they are simple to measure (as the difference in chemical shift between a diamagnetic and paramagnetic sample) and encompass both distance and orientation information of nuclei relative to the magnetic susceptibility anisotropy (Δ*χ*) tensor. The PCS of any nuclear spin can be back-calculated from knowledge of the Δ*χ*-tensor:
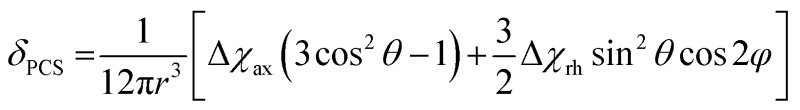
where Δ*χ*_ax and_ Δ*χ*_rh_ are the axial and rhombic components of the Δ*χ*-tensor and *r*, *θ* and *φ* are the polar coordinates of the nuclei with respect to the principal axes of the Δ*χ*-tensor. The *r* ^–3^ distance dependence of PCSs allows them to be measured for nuclei up to 40 Å or more away from the metal ion.^4^ Thus, PCSs provide long-range structural information that can be utilised in the study of protein structure and conformation,^5–9^ protein–protein^10–13^ and protein-small molecule interactions,^14–17^ and even *de novo* protein structure determination.^18,19^

Paramagnetic lanthanide(iii) ions, except Gd^3+^, can be used to induce PCSs in the NMR spectra of macromolecules. Their anisotropic magnetic susceptibilities are inherently large (yet different) and, combined with their similar structure and bonding, allow the substitution of one lanthanide ion for another as a convenient route to vary the magnetic properties of a sample. However, most proteins do not natively bind lanthanide ions, which has spurred recent interest in the design of synthetic lanthanide-binding tags (LBTs)^3,20^ or peptides^21,22^ capable of introducing lanthanide ions into proteins in a site-specific manner.

Lanthanide ions are “hard” Lewis acids that can adopt high coordination numbers, thus polydentate ligands featuring hard bases (such as O and N atoms) are ideal candidates to ensure tight lanthanide ion binding. Rigidity of the lanthanide relative to the protein frame is paramount to prevent the deleterious averaging effects of tag mobility on measured PCSs and RDCs, which tend towards zero with increasing motion. Engineering tagging sites to take advantage of additional coordination to acidic side-chains of proteins,^23–25^ conjugation to proteins through multiple sites of attachment^26,27^ or the use of steric bulk^28^ have been successful strategies to limit tag mobility. It is imperative that the attached LBT must also give rise to a single observable species in solution, as multiple species in *slow* exchange can lead to highly complex spectra that are of limited practical utility.^29–31^

LBTs that bind lanthanide ions extremely tightly, without the need for additional protein interactions, are particularly attractive. They allow the study of proteins in the presence of their own native metal ions and metal ion-bound cofactors (*e.g.* metalloproteins and kinases) and remove any problems associated with excess free lanthanide ions that can result in line broadening in the NMR spectra. LBTs based on DOTA (1,4,7,10-tetraazacyclododecane-1,4,7,10-tetraacetic acid) have proved to be useful, having induced significant PCSs and RDCs in several proteins.^28,32,33^ Although capable of binding lanthanides with dissociation constants of the order of 10^–23^ to 10^–25^ M,^34^ lanthanide complexes of DOTA display a dynamic behaviour in solution at ambient temperature. Inversion of the cyclen ring (defined by the NCCN torsion angle as either *δδδδ* or *λλλλ*) and rotation of the pendant arms (defined by the NCCO torsion angles as either *Δ* or *Λ*) result in a dynamic equilibrium between square anti-prismatic (SAP) and twisted square anti-prismatic (TSAP) coordination geometries.^35^ When bound to a protein, this can lead to the presence of multiple stereoisomers in *slow* exchange, each producing their own paramagnetic effects that greatly complicate analysis of the spectra. In order to limit these conformational exchange processes and simplify the spectra, successful DOTA-based LBT designs have incorporated chiral elements into the pendant arms or cyclen ring,^28,33^ or employed two-points of conjugation to the protein.^32,36^

**Fig. 1:**
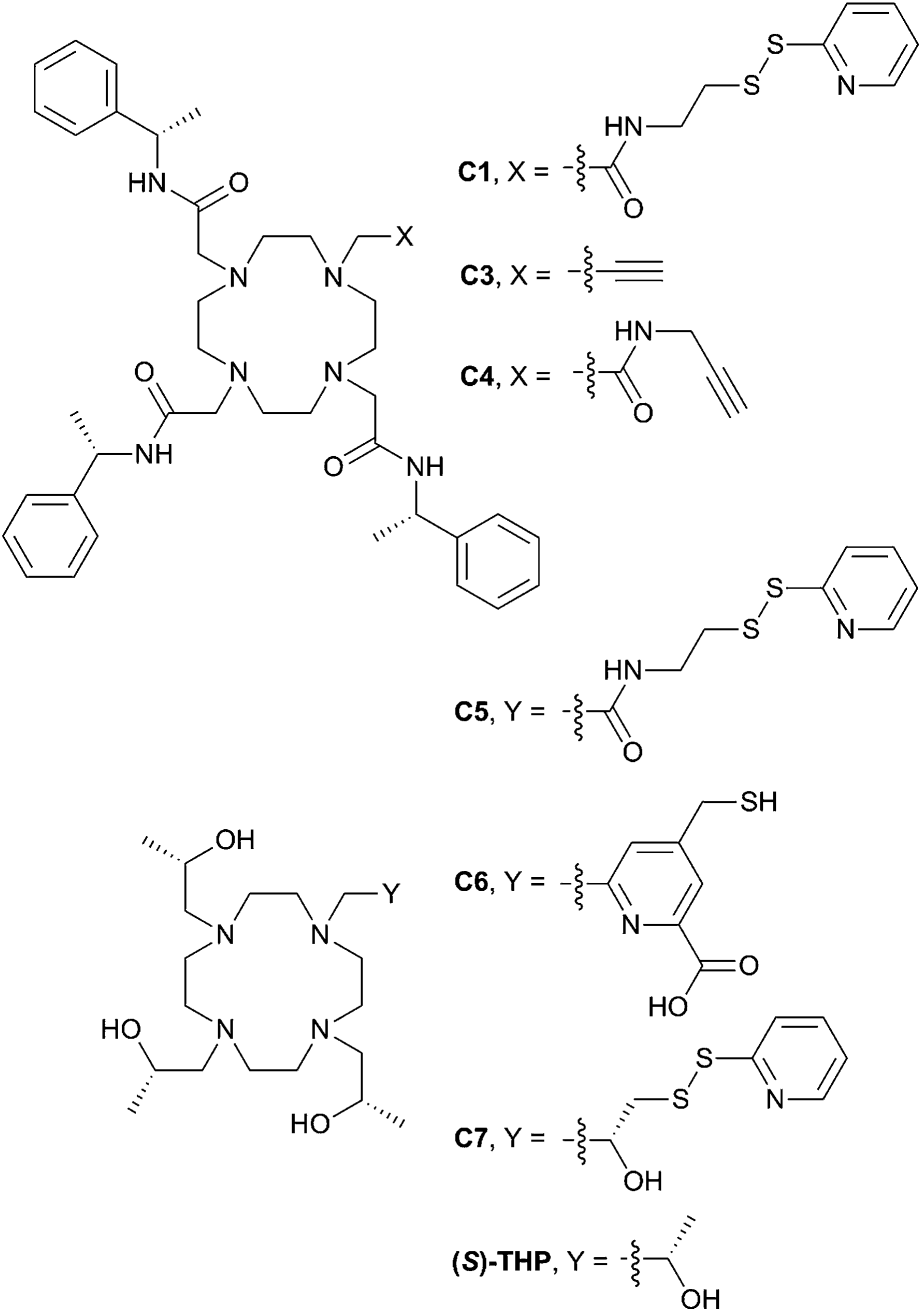
Existing and newly developed LBTs referred to in the text. **C2** and **C8** are the enantiomers of **C1** and **C7**, respectively.

Previously, we demonstrated that attachment of three sterically bulky (*S*)- or (*R*)-phenethylacetamide pendant arms to a 1,4,7,10-tetraazacyclododecane (cyclen) macrocyclic ring (**C1–C4**, Fig. 1) was sufficient to generate a single apparent stereoisomer and to limit tag flexibility, allowing the observation of measureable and sizable paramagnetic effects.^28,37^ However, for some proteins in our laboratory, such as 6-hydroxymethyl-7,8-dihydropterin pyrophosphokinase (HPPK), this family of tags was found to present issues in terms of protein stability, as evidenced by an increased tendency to precipitate during and post conjugation. We suggest this to originate from the incompatibility of the large hydrophobic nature of this tag series with these proteins. It is also foreseeable that the tags' hydrophobic character could complicate the study and screening of weak ligand–protein interactions, as small hydrophobic compounds (*e.g.* from fragment libraries) can potentially associate transiently with the LBT, leading to a transferred PCS effect and a skewed, “meaningless” average ligand PCS.

With this in mind, we have now developed a new series of tags that are much more structurally compact and hydrophilic in nature (**C5–C8**, Fig. 1). These tags are based on ((2*S*,2′*S*,2′′*S*,2′′′*S*)-1,1′,1′′,1′′′-(1,4,7,10-tetraazacyclododecane-1,4,7,10-tetrayl)tetrakis(propan-2-ol)) (**(*S*)-THP**), a cyclen derivative featuring four chiral (*S*)-2-hydroxypropyl pendants. Multiple **(*S*)-THP-Ln^3+^** (where Ln = La, Ce, Nd, Eu, Yb or Lu) complexes have been reported to show ^1^H NMR spectra that display a single set of resonances,^38–40^ which suggested that an **(*S*)-THP** based LBT could also produce a single set of PCSs to nuclei of a bound protein. The **(*S*)-THP-Yb^3+^** complex specifically has been shown to adopt a *Λ*(*λλλλ*) TSAP geometry in solution.^39^

Conjugation of single-point attachment LBTs to proteins requires less prior structural knowledge of the target, fewer mutations for their introduction and can still produce useful effective Δ*χ*-tensors when tag movements are limited.^41^ Thus, our initial focus has been on the development of **(*S*)-THP** derivatives featuring a single thiol-conjugatable group, so as to produce tags applicable to the study of as wide a range of protein systems as possible. The first of these (**C5**) utilises the same pyridyl disulfide-activated linker as our earlier reported **C1** and **C2** tags. Given the absence of the sterically bulky pendants of the latter tags, which were postulated to be an important element in limiting tag flexibility,^28^ it was anticipated that this tag might prove too mobile for NMR applications. Therefore, analogues with shorter linker groups were also engineered. **C6** features a bidentate chelating 2-carboxylpyridine moiety with a conjugatable methylmercaptan group attached to the 4-position of the pyridine ring, and can be viewed as a hybrid of **(*S*)-THP** and the various DPA-based LBTs reported by Otting and co-workers.^23,42–44^ **C7**, and its enantiomer **C8**, feature possibly the smallest practicable modification to **(*S*)-THP** that allows for bioconjugation: a pyridyl disulfide group is attached directly to one of the four chiral 2-hydroxypropyl pendants and the resulting protein-conjugated tags feature only a disulfide bond between the chirally pure **(*S*)/(*R*)-THP-Ln^3+^** chelate and protein.

We now report the synthesis of the new tags (**C5–C8**) and demonstrate their utility in paramagnetic NMR structural studies using human ubiquitin and GB1 as model proteins, as well as the antimicrobial target, HPPK.^45^ As detailed below, the **C5** and **C6** tags are found to perform comparably to **C1** in terms of the magnitude of the Δ*χ*-tensors observed on ubiquitin. More significantly, however, the **C7** and **C8** tags produce considerably larger paramagnetic effects, indicating that the short linker present within these tags translates to a more restricted lanthanide ion attachment to the protein.

## Results and discussion

### Tag synthesis

**Scheme 1:**
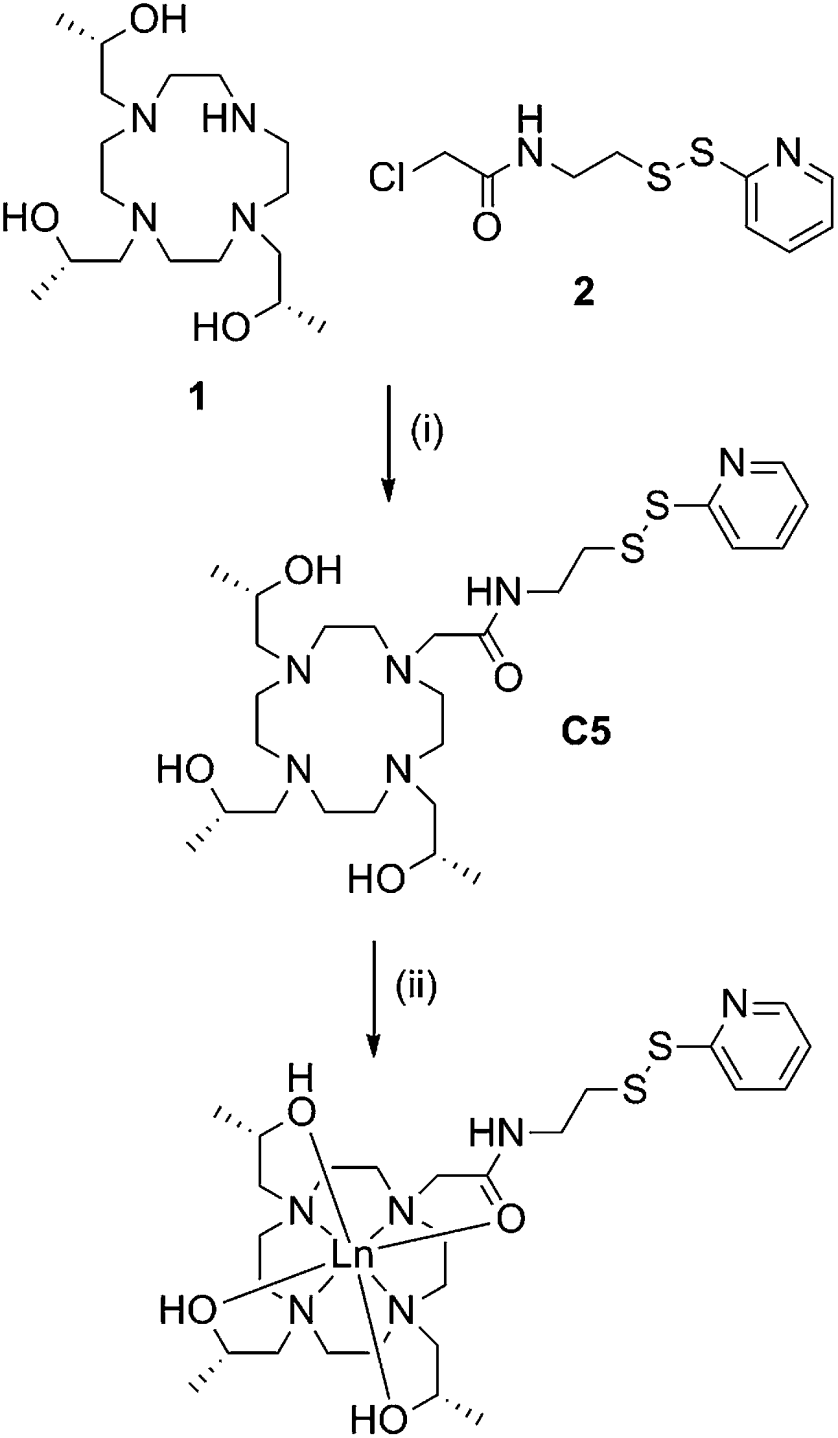
Synthesis of **C5** and its lanthanide complexes. Reagents and conditions: (i) DIPEA, ACN, RT, 72 h, 61%; (ii) LnCl_3_, ACN, H_2_O, pH 7, reflux, overnight, quant.

**C5** was prepared in good overall yield by nucleophilic substitution between the previously reported compounds, (1*S*,4*S*,7*S*)-1,4,7-tris(2-hydroxypropyl)-1,4,7,10-tetraazacyclododecane^46^ (**1**) and 2-chloro-*N*-(2-(pyridin-2-yldisulfanyl)ethyl)acetamide.^28^ (**2**) (Scheme 1).

**Scheme 2:**
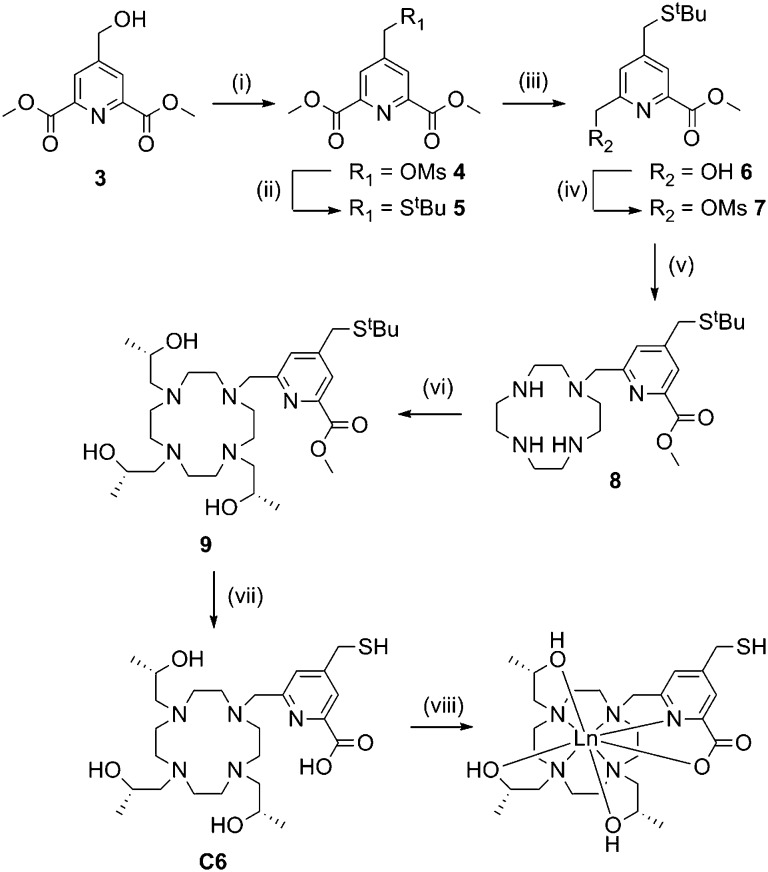
Synthesis of C6 and its lanthanide complexes. Reagents and conditions: (i) MsCl, DIPEA, DCM, 0 °C, 30 min, quant; (ii) ^*t*^BuSH, NaH, DMF, RT, 5 min, 47%; (iii) NaBH_4_, MeOH, DCM, RT, 2 h, 64%; (iv) MsCl, DIPEA, DCM, 0 °C, 30 min, 77%; (v) cyclen, CHCl_3_, RT, O/N, quant.; (vi) (*S*)-propylene oxide, MeOH, RT, 48 h, quant.; (vii) HCl (32%), reflux, 4 h, 85%; (viii) LnCl_3_, ACN, H_2_O, pH 7, reflux, overnight, quant.

Synthesis of **C6** (Scheme 2) required preparation of a novel carboxyl pyridine linker. Dimethyl 4-(hydroxymethyl)pyridine-2,6-dicarboxylate (**3**) was prepared following literature procedures^23^ and converted to the *tert*-butyl thioether **5** *via* the mesylate derivative **4**. Partial reduction with sodium borohydride and mesylation of the resulting hydroxyl group yielded **7**, which was reacted with an excess of cyclen to form **8**. Reaction with an excess of (*S*)-propylene oxide, followed by ester and *tert*-butyl deprotection yielded **C6**. We attempted to activate the thiol of **C6** as a pyridyl disulfide, however the resulting product was unstable during purification, thus the free thiol was used for tagging (*vide infra*).

Metal complexes of **C5** and **C6** were prepared by heating the relevant tag with two equivalents of XCl_3_ salts (X = Y, Dy, Tb, Tm or Yb) at 80 °C in a water–acetonitrile mixture buffered at neutral pH overnight. Coordination of these tags was generally close to quantitative, with excess metal ions and uncomplexed tag removed *via* HPLC purification.

**Scheme 3:**
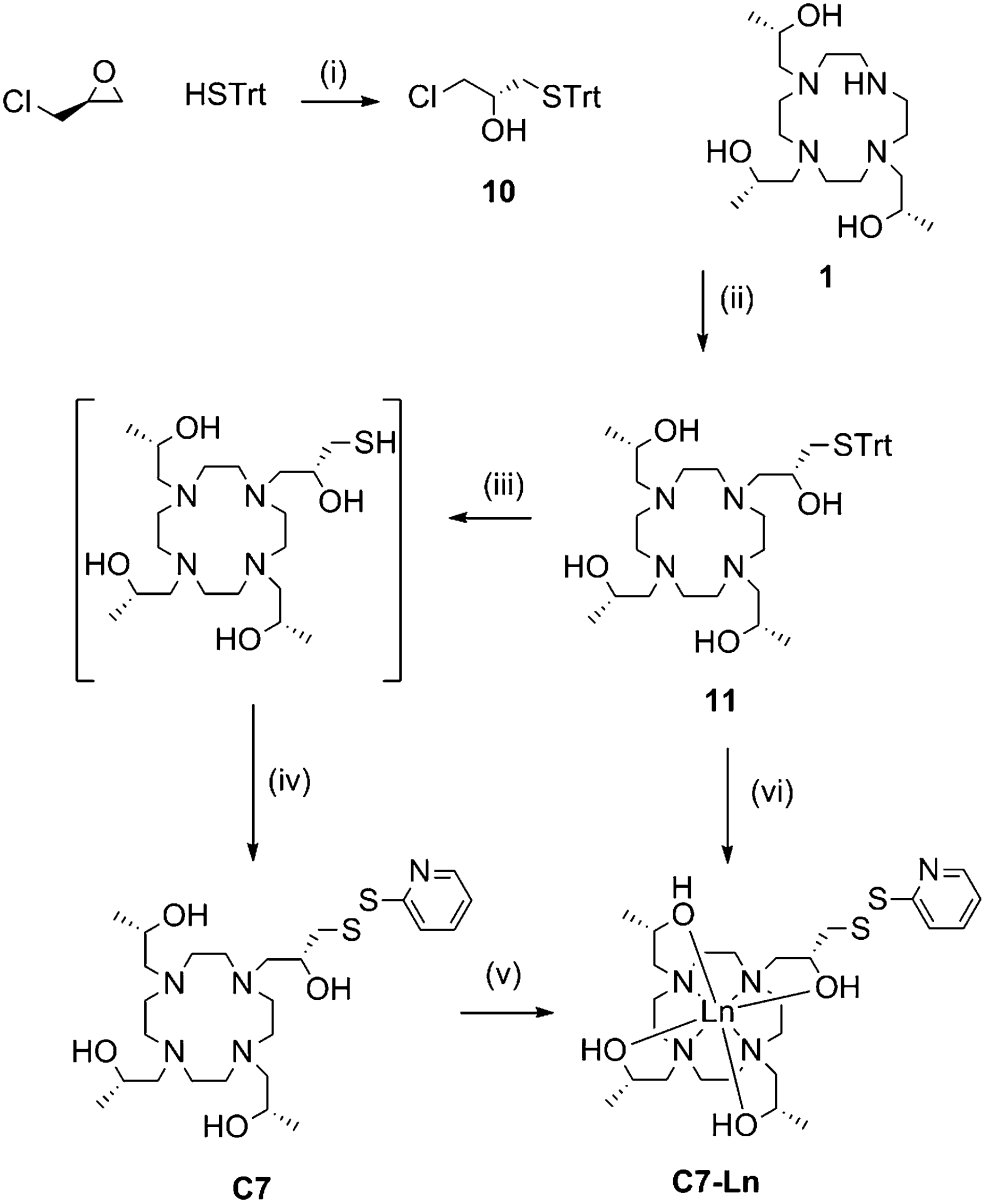
Synthesis of **C7** and its lanthanide complexes. Reagents and conditions: (i) KF, MeOH, RT, 72 h, 95%; (ii) K_2_CO_3_, ACN, reflux, overnight, 60%; (iii) TFA, triethylsilane, DCM, RT, 1 h; (iv) 2,2′-dipyridyldisulfide, MeOH, RT, 15 min, 39% (from **11**); (v) LnCl_3_, EtOH, DIPEA, reflux, overnight; (vi) LnCl_3_, MeOH, reflux, 4 h, 2,2′-dipyridyl disulfide, silver nitrate, RT, 2 h, 34%.

Due to the favourable properties of **C7** (*vide infra*), its synthesis underwent several iterations in order to improve the yield (Scheme 3). Similarly to **C5** and **C6**, the initial method involved synthesis of the tag, followed by metal ion complexation.

Ring opening of (*S*)-epichlorohydrin with triphenylmethane thiol, in the presence of potassium fluoride, produced **10** in excellent yield (95%). **10** then underwent nucleophilic substitution by heating with **1** and potassium carbonate to form **11**. Deprotection of the trityl group was carried out at room temperature with trifluoroacetic acid and triethylsilane. Subsequent thiol activation with 2,2′-dipyridyl disulfide and purification *via* HPLC produced **C7** in 39% yield from **11**.

Formation of **C7-Ln^3+^** complexes was extremely slow in the presence of water and required heating in anhydrous ethanol. Complexation was still relatively slow compared to the other tags. Furthermore, if left for a prolonged period of time (*e.g.* greater than 48 h) noticeable amounts of disulfide rearrangement would occur, resulting in a chelate dimer and regeneration of 2,2′-dipyridyl disulfide. **C7-Ln^3+^** complexes formed this way were thus generally purified from a mixture with uncomplexed **C7**, before a significant amount of disulfide rearrangement could occur, resulting in relatively poor yields.

Various attempts to optimise **C7** complexation were made, including initial passage of **C7** over anion exchange resin (to remove trifluoroacetic acid, present from prior HPLC purification) and addition of organic or inorganic bases to complexation reactions. However, we eventually found the most practical way of producing **C7-Ln^3+^** complexes to be by forming metal complexes of **11**, before trityl deprotection and thiol activation to the final product. Compound **11** was isolated as a neutral compound and readily formed **11-Ln^3+^** without side-product formation, by heating for several hours in methanol with two equivalents of the relevant metal chloride salt. **11-Ln^3+^** was then trityl deprotected with silver nitrate and thiol activated with 2,2′-dipyridyl disulfide, before reverse-phase HPLC purification to yield **C7-Ln^3+^**. This method allowed the formation of **C7-Ln^3+^** complexes from **11** in “one pot” and required one less round of HPLC purification compared to the previous route, resulting in overall higher yields (34% from **11**). The **C8** tag and complexes followed the same procedures with the replacement of (*S*)-propylene oxide and (*S*)-epichlorohydrin with their (*R*)-enantiomers.

Fig. S1–S3† show the ^1^H NMR spectra of the Yb^3+^ complexes of **C5–C7**. Although greatly complicated by the pyridyl disulfide linker breaking the symmetry of the complex, the ^1^H NMR spectrum of **C7-Yb^3+^** bears some resemblances to that of the **(*S*)-THP-Yb^3+^** complex reported by Lelli *et al.* ^39^ Comparing the most resolved signals, the peak at –28 ppm in **(*S*)-THP-Yb^3+^** is split into four overlapping peaks of equal intensity in **C7-Yb^3+^**, while the peak at 52 ppm in **(*S*)-THP-Yb^3+^** is split into three peaks in **C7-Yb^3+^**, one of which is twice the intensity of the other two. The ^1^H NMR spectra of the more structurally-varied **C5-Yb^3+^** and **C6-Yb^3+^** complexes show fewer similarities to **(*S*)-THP-Yb^3+^**. We did not attempt a complete assignment of the ^1^H NMR spectra of the **C5–C7** complexes.

### Testing of tags on a cysteine-bearing mutant of ubiquitin

A human ubiquitin A28C mutant was used as an initial model protein to assess the effects of the paramagnetic properties of each tag. Purified protein was first stirred with ten equivalents of DTT to reduce any oxidised cysteines. Excess DTT was removed by passage over a PD10 column equilibrated with 50 mM HEPES, pH 8.0. For the pyridyl disulfide-containing tags, **C5**, **C7** and **C8**, five equivalents of the relevant lanthanide-complexed tag were added and the solutions stirred for 2 h at room temperature, before excess tag was removed by passage over a PD10 column. Tagging yields varied between 70% to quantitative, as determined by NMR analysis.

In order to conjugate **C6**, reduced protein was first reacted with a ten-fold excess of DTNB for 1 h, before passage over a PD10 column followed by the addition of five equivalents of **C6-Ln^3+^** complex. The reaction was allowed to stir for 2 h at room temperature, before removal of excess tag *via* a PD10 column. Tagging yields were generally quantitative.

**Fig. 2:**
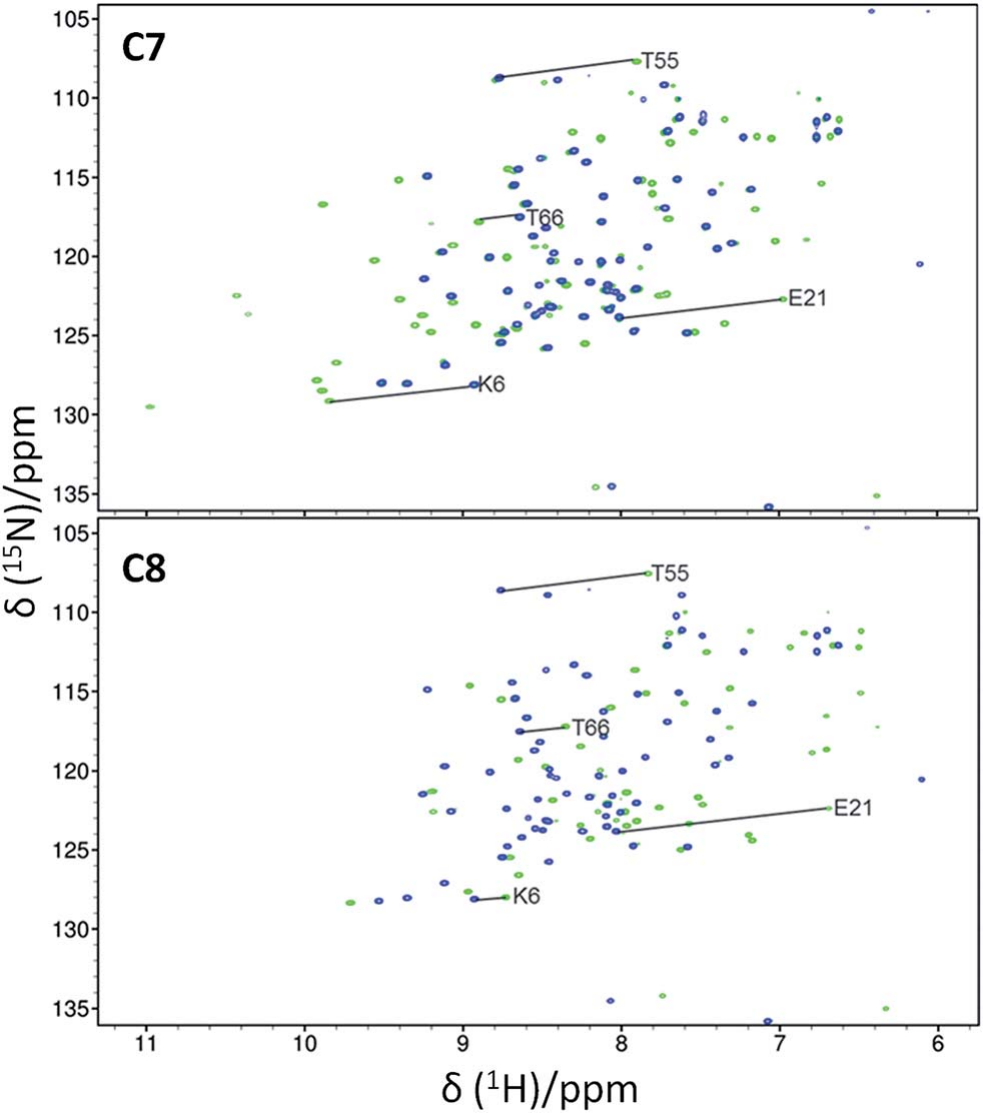
Overlays of ^15^N-HSQC spectra of **C7** (top spectra) and **C8** (bottom spectra) tagged ubiquitin A28C, loaded with either Y^3+^ (blue) or Tm^3+^ (green). The spectra were recorded at 25 °C and pH 8.0 at a ^1^H NMR frequency of 600 MHz. Selected PCSs are indicated with solid lines.

**Fig. 3:**
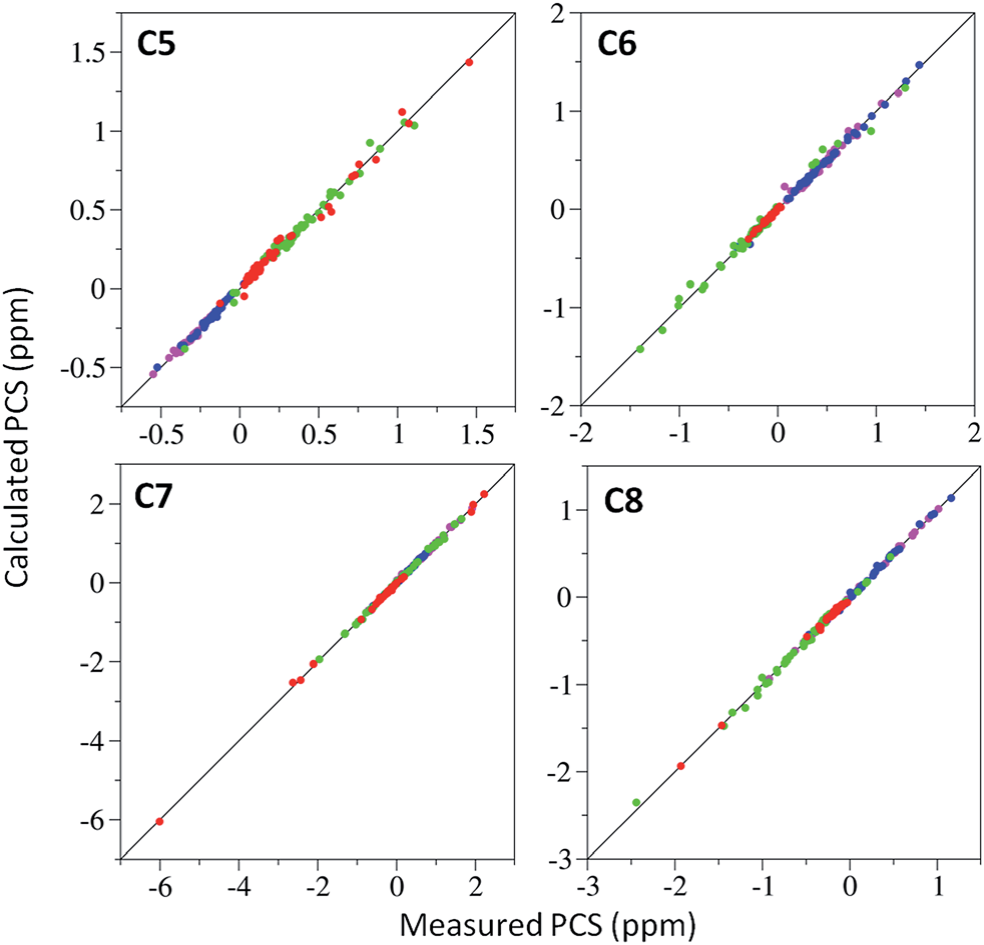
Correlations between experimental and back-calculated PCSs for **C5–C8** bound to ubiquitin A28C loaded with either Dy^3+^ (magenta), Tb^3+^ (blue), Tm^3+^ (green) or Yb^3+^ (red). Solid lines represent perfect correlation.

^15^N-HSQC spectra of each lanthanide complex conjugated to ubiquitin A28C showed significant PCSs (Fig. 2 and S7–S9†). For each tag, the Y^3+^ complex produced minor chemical shift perturbations relative to the untagged protein, with larger shifts limited to residues in the vicinity of the tagging site. In each spectrum, only a single set of PCSs was observed. PCSs were measured as the difference in chemical shift of resonances between the paramagnetic (Dy^3+^, Tb^3+^, Tm^3+^ or Yb^3+^) and diamagnetic (Y^3+^) tagged samples. The Δ*χ*-tensors were determined by fitting the measured PCSs (Tables S2 and S3†) to the first conformer of the NMR structure of ubiquitin (PDB ID ; 2MJB),^47^ both individually for each metal ion (Table 1) and simultaneously for each complex of a given tag with a common metal ion position (Table S4,† *vide infra*). Fig. 3 shows the correlations between measured and back-calculated PCSs for the individually derived Δ*χ*-tensors, demonstrating their high quality, which is also reflected in the low *Q*-values.

**Table 1:** Δ*χ*-Tensor parameters for **C5–C8** tagged ubiquitin A28C^*a*^ ^,^ ^*b*^

Tag	Ln^3+^	# PCS	Δ*χ*_ax_	Δ*χ*_rh_	*Q*	*x*	*y*	*z*	*α*	*β*	*γ*
**C5**	Dy^3+^	39	8.2	5.3	0.04	2.908	2.285	–15.138	141	88	71
	Tb^3+^	47	9.4 (0.5)	2.2 (0.1)	0.06	2.308	–0.421	–17.179	157	95	114
	Tm^3+^	47	–18.7 (1.7)	–6.9 (0.4)	0.06	4.728	–3.051	–17.815	127	96	100
	Yb^3+^	61	–6.7 (0.4)	–2.1 (0.2)	0.08	0.857	–2.115	–18.247	120	97	122
**C6**	Dy^3+^	49	–9.4	–5.9	0.07	8.694	3.797	–11.227	45	49	80
	Tb^3+^	47	–14.6 (0.4)	–3.6 (0.1)	0.04	7.019	2.304	–13.622	44	68	97
	Tm^3+^	51	11.5 (0.7)	4.1 (0.4)	0.10	7.018	3.097	–12.773	41	71	130
	Yb^3+^	51	2.0 (0.1)	0.9 (0.0)	0.08	9.178	2.136	–12.736	42	60	103
**C7**	Dy^3+^	35	26.6 (1.1)	6.0 (0.5)	0.03	–0.734	–3.238	–13.305	71	29	32
	Tb^3+^	40	11.7 (0.3)	1.7 (0.3)	0.04	–1.901	–3.712	–14.371	47	44	59
	Tm^3+^	44	–19.4 (0.7)	–7.8 (0.9)	0.03	–4.314	–1.357	–13.717	9	62	104
	Yb^3+^	51	5.8	3.0	0.03	–0.427	–0.482	–14.129	16	110	85
**C8**	Dy^3+^	28	31.2 (0.7)	7.1 (0.6)	0.02	1.810	–3.922	–13.760	91	44	5
	Tb^3+^	37	14.3 (0.6)	5.1 (0.7)	0.05	2.003	–1.928	–13.959	73	38	19
	Tm^3+^	43	–16.3	–10.2	0.04	0.862	–3.716	–15.075	67	26	46
	Yb^3+^	46	–4.2 (0.1)	–1.9 (0.2)	0.04	1.679	–3.184	–14.267	140	22	169

^*a*^The axial and rhombic components of the Δ*χ*-tensors are reported in units of 10^–32^ m^3^, and the Euler angles in degrees, using the *zyz* convention and unique tensor representation.^53^ Standard deviations (in brackets) were determined from random removal of 10% of the PCSs and recalculating the Δ*χ*-tensor 1000 times, in some cases the *z* and *y* axes of the tensor were of similar magnitude and swapped in different fits, thus standard deviations were not determined. Quality factors (*Q*) were calculated as the root-mean-square deviation between the experimental and back-calculated PCSs divided by the root-mean-square of the experimental PCSs.

^*b*^Metal ion coordinates (*x*, *y*, *z*) are reported relative to the NMR structure of ubiquitin (PDB ID 2MJB ^47^).

Different p*K*_a_ values for the deprotonation of a single alcohol pendant (or, possibly, bound water molecule) have been reported for different **(*S*)-THP-Ln^3+^** complexes. These range from 8.4 for the lanthanum complex to 6.4 for lutetium, with a trend of decreasing p*K*_a_ across the lanthanide series.^39,48^ Thus, at a given pH, different **(*S*)-THP-Ln^3+^** complexes can exist across a range of equilibria between +3 and +2 charged states.

To investigate a potential change in the properties of different **(*S*)-THP-Ln^3+^** tags with pH, we re-recorded the spectra of the **C7-Ln^3+^**-tagged ubiquitin samples at pH 6.5 (Fig. S10 and S11†). Most notably, the PCSs of the Dy^3+^-tagged sample were much smaller at pH 6.5 compared to pH 8 (slope 0.28, *R* ^2^ 0.55). The majority of the Tb^3+^ PCSs were also reduced at lower pH, though to a lesser degree than for Dy^3+^ (slope 0.47, *R* ^2^ 0.62). In contrast, the size of the PCSs observed in the Tm^3+^ (slope 0.95, *R* ^2^ 0.98) and Yb^3+^ (slope 0.94, *R* ^2^ 0.99) samples was not significantly affected by the change in pH. The pH-dependence of the PCSs induced by each **C7-Ln^3+^** complex likely reflects protonation/deprotonation processes involving the pendants arms and/or aquo ligands, leading to changes in the average charge and coordination geometry of each complex, and thus potentially their interactions with the protein surface and resulting metal ion positions. At both pHs, each **C7-Ln^3+^** complex produced a single PCS for each affected nucleus, indicating that any processes such as protonation/deprotonation (and their effect on coordination geometry) are *fast* on the NMR timescale, thus the spectra are straightforward to interpret. The Tm^3+^ and Yb^3+^ complexes are likely to prove of most practical use over a wider, biologically relevant pH range.

For the data recorded at pH 8, we determined Δ*χ*-tensors for lanthanide ions both individually, allowing independent metal ion positions (Table 1), and simultaneously with a common metal ion position for complexes of a given tag (Table S4†). In some cases there were significant differences between the individually and simultaneously determined Δ*χ*-tensors, with the Δ*χ*_ax_ component varying by up to 49% for the most extreme example of the **C5-Dy^3+^** complex. Despite this, the *Q*-values of Δ*χ*-tensors determined from either method were very respectable (0.02–0.12), demonstrating that the tensors from either approach are suitable for structural investigations. Individually derived Δ*χ*-tensors produced *Q*-values that were universally lower than those of the simultaneously calculated tensors; however, the individually determined metal ion positions were up to 6.2 Å apart for different complexes of the same tag. This observation of different metal ion positions in individual Δ*χ*-tensor fits has been noted previously^24,49^ and in those cases was attributed to the uncertainty in determining the metal ion position during the fitting procedure, which can also depend on the coverage and distribution of the PCSs over the tensor “space”. Thus, a common metal ion position that satisfactorily describes the PCSs of each metal ion is often used to increase stability of the metal ion coordinate and tensor components during the fitting. In this case, the apparent different sensitivities of each metal complex to pH (influencing their average charge, coordination geometry and possible interactions with the protein surface) could be seen as justification for the use of individually determined metal ion positions and Δ*χ*-tensors. It is worth noting again that mobility of the metal ion, for instance due to flexibility of the tag linker, results in averaging of PCSs. By fitting a single tensor to these averaged values, we are describing an “effective Δ*χ*-tensor”. The metal ion coordinate associated with this tensor should not be interpreted as a definitive point at which the metal ion is statically located.^41^ Unless specified otherwise, figures and values presented herein were derived using Δ*χ*-tensors corresponding to individual metal ion positions.

### Comparison of performance of the new tags with **C1**

With few exceptions, the lanthanide complexes of the new tags produced Δ*χ*-tensors with Δ*χ*_ax_ components of similar or greater magnitude to those of the corresponding **C1** tag conjugated to the same ubiquitin mutant.^28^ This is a particularly interesting and non-intuitive observation in the case of **C5**, as it suggests that any increase in mobility of the tag, due to the loss of the bulky phenyl amide pendants of **C1**, is compensated for by the altered coordination environment and ligand field associated with the alcohol pendants and/or changes in secondary interactions with the protein, allowing **C5** to generate sizeable paramagnetic effects.

In contrast to the case for **C1**, for which each lanthanide complex reliably produces PCSs of a predictable relative size and sign for a given nuclear spin (*e.g.* Tm^3+^ and Tb^3+^ PCSs are generally opposite in sign, with Tb^3+^ PCSs slightly larger in size), the relative order and size of PCSs induced by the new tags loaded with different lanthanide ions was quite variable (Fig. S12†). Correspondingly, the determined Euler angles of the Δ*χ*-tensors from metal complexes of the same tag also varied to a larger extent than those observed for **C1** (Fig. S13†), suggesting changes in coordination environment with each lanthanide ion, as alluded to above. The noted change in the orientation of the Δ*χ*-tensor for each metal complex of the same tag is potentially a useful property, which can help resolve the redundant solutions that can be encountered in studies using PCSs (associated with the symmetry of the Δ*χ*-tensor), without requiring multiple tagging sites or tags.^50^

Initially, only the (*S*)-enantiomer of each tag was synthesised and assessed. However, given the large Δ*χ*-tensors and excellent fits observed for **C7**, its enantiomer **C8** and the corresponding **C8-Ln^3+^** complexes were also synthesised and conjugated to ubiquitin A28C. Despite the same coordination environment of the lanthanide ions in complexes of either tag enantiomer, different PCSs, Δ*χ*-tensors and metal ion positions (Fig. 2, 3, S8 and S12; Tables 1, S3 and S4†) were observed, likely due to the differences in their interaction with the chiral protein surface, arising from the opposite stereochemistry of the pendant arms. On average, the Δ*χ*_ax_ components of each complex of the **C7** and **C8** tags were larger than those of the **C1**, **C5** or **C6** tags on ubiquitin A28C, suggesting that the very short linker is key to the tags' superior paramagnetic effects. A temperature titration of the **C7-Tm^3+^** tagged sample showed no signs of additional cross-peaks due to conformational exchange (Fig. S14†), although at higher temperatures the observed PCSs were smaller, presumably due to increased tag mobility.

**Fig. 4:**
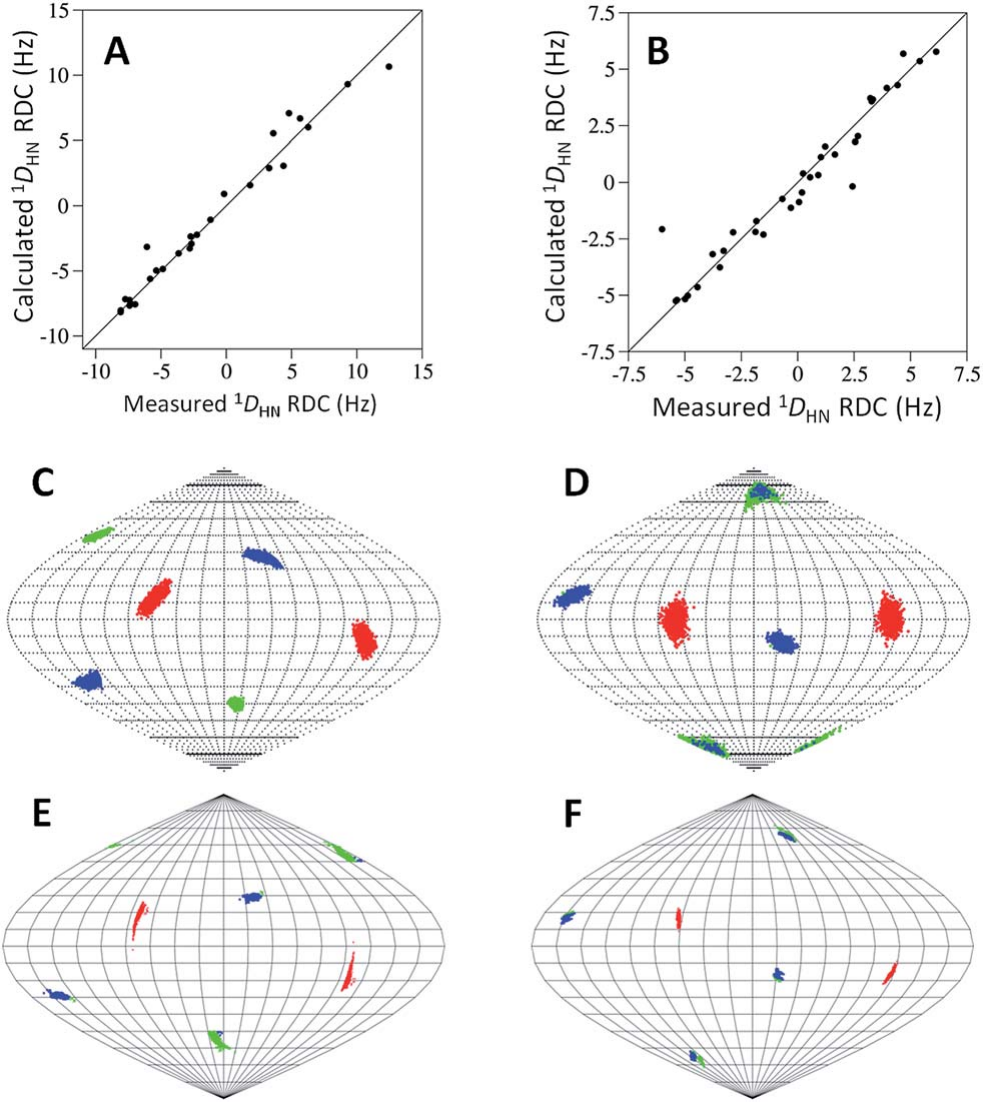
Correlations between experimental and calculated ^1^ *D*_HN_ RDCs recorded at a ^1^H NMR frequency of 600 MHz for **C7-Tm^3+^** (A) and **C8-Tm^3+^** (B) tagged ubiquitin A28C. Solid lines represent perfect correlation. Orientations of the principal axes of the alignment (C **C7**, D **C8**) and Δ*χ* (E **C7**, F **C8**) tensors. The points show where the principal axes of the tensors penetrate the sphere with the axes coloured as follows: *z* (blue), *y* (green), *x* (red). For the alignment tensors, 1000 replicates of SVD calculation using the structural noise Monte-Carlo method (‘-mcStruc’) within PALES are shown. For the Δ*χ*-tensors, 1000 replicates with a random 10% of the PCS data removed each time are shown. The convention |*z*| > |*y*| > |*x*| is used to name the axes, resulting in swapping of the |*z*| and |*y*| axes in different fits when their magnitudes are similar.

In order to investigate the rigidity of the **C7** and **C8** tags and their ability to induce partial alignment in the magnetic field, one bond ^1^H–^15^N RDCs (^1^ *D*_HN_) of the Tm^3+^ complexes of **C7** and **C8** were measured relative to the Y^3+^-tagged protein. ^1^ *D*_HN_ RDCs up to 12.5 and 6.1 Hz were observed at 600 MHz for **C7** and **C8** respectively. Alignment tensors were determined by fitting the measured RDCs (Table S5†) to a structure of ubiquitin using single value decomposition within PALES^51^ (Table S6†). The measured and calculated ^1^ *D*_HN_ RDCs (Fig. 4A and B) are in good agreement and the principal axes of the alignment (Fig. 4C and D) and Δ*χ* (Fig. 4E and F) tensors are very similar, demonstrating that the orientation of the tensors are relatively well defined for either enantiomer. The Δ*χ*-tensor components derived from the alignment tensor parameters match very favourably with the PCS derived Δ*χ*-tensor values for **C7-Tm^3+^** (Tables 1 and S6; eqn S1†). However, for **C8-Tm^3+^**, the alignment tensor predicted Δ*χ*_ax_ and Δ*χ*_rh_ are 62% and 54% of their respective PCS determined values, suggesting some degree of mobility is still present. It is not uncommon for alignment tensors to be smaller than Δ*χ*-tensors.^27,49,52^ This is partly attributed to the greater sensitivity of RDCs to protein and tag movements than PCSs. The *Q*-factors of the alignment tensors are larger than those of the Δ*χ*-tensors. Due to the chiral nature of the tags, **C7** may be engaged in different secondary interactions with the protein, helping to limit its mobility to a greater degree than **C8**.

### Further testing of **C7** and **C8** on a cysteine-bearing mutant of GB1 and HPPK

To demonstrate the general utility of the **C7** and **C8** tags, lanthanide complexes of both tags were conjugated to a GB1 Q32C mutant. Each sample produced a single set of PCSs from which Δ*χ*-tensors were determined (Fig. S15 and S16; Tables 2, S7 and S8†). Differences between individual and simultaneously derived Δ*χ*-tensors were apparent, though to a lesser extent than observed for ubiquitin. Both Tb^3+^ complexes, but particularly the **C7** complex, resulted in only small PCSs and significantly smaller Δ*χ*-tensors on GB1 compared to ubiquitin. Thus, the GB1 spectra, which were recorded at pH 6.5, seem consistent with the ubiquitin spectra recorded at pH 6.5, in that they suggest that the Tb^3+^ (and likely Dy^3+^) complexes are of less practical use at a lower pH. Conversely, both Tm^3+^ complexes resulted in sizable PCSs and Δ*χ*-tensors. The Δ*χ*_ax_ components of **C7-Tm^3+^** and **C8-Tm^3+^** on GB1 Q32C are 73% and 91% of their respective values on ubiquitin A28C, demonstrating the influence of the tagging site and protein environment on the tags' performance.

**Table 2:** Δ*χ*-Tensor parameters for **C7** and **C8** tagged GB1 Q32C and **C7** tagged HPPK S112C/C80A^*a*^ ^,^ ^*b*^

Protein	Tag	Ln^3+^	# PCS	Δ*χ*_ax_	Δ*χ*_rh_	*Q*	*x*	*y*	*z*	*α*	*β*	*γ*
GB1	**C7**	Tb^3+^	47	2.2 (1.1)	1.0 (0.7)	0.09	29.244	29.993	13.297	18	39	60
		Tm^3+^	37	–14.2 (0.9)	–4.4 (0.5)	0.06	31.745	29.577	12.618	145	56	80
	**C8**	Tb^3+^	40	6.1 (0.4)	3.1 (0.3)	0.05	33.668	30.260	14.419	155	41	175
		Tm^3+^	40	–14.9 (0.4)	–6.4 (0.6)	0.06	34.227	32.261	17.019	172	73	173
HPPK	**C7**	Tm^3+^	81	54.5 (0.5)	12.5 (0.5)	0.04	14.304	13.802	13.906	149	55	127

^*a*^See footnote a in Table 1.

^*b*^Metal ion coordinates (*x*, *y*, *z*) for each tag are relative to the crystal structures of GB1 (PDB ID 1PGA)^54^ or HPPK (PDB ID 3QBC).^45^

This variability was further observed in our investigations of the 20 kDa-sized protein HPPK (to be fully reported elsewhere). Tagging at different sites produced Δ*χ*-tensors with varied Δ*χ*_ax_ components, up to 54.5 × 10^–32^ m^3^ for a HPPK S112C/C80A mutant tagged with **C7-Tm^3+^** (Fig. S16 and S17; Tables 2 and S9†). Given that the PCS and RDC data for **C7-Tm^3+^** tagged ubiquitin A28C had previously indicated that the chelate was relatively rigid on ubiquitin, such an increase in the Δ*χ*_ax_ component for HPPK S112C/C80A was highly unexpected. Spectra of both proteins were recorded at pH 8, thus different deprotonation/protonation equilibria based on solvent water alone are insufficient to explain such variance. However, different interactions with the protein surface could also affect the charged state of the tag. For this particular HPPK mutant, the cysteine for tagging was introduced on the β-sheet of a short β-hairpin, which features an aspartic acid (D107) on the adjacent β-sheet. The calculated metal ion position is above and between D107 and S112, which both point in the same direction in the HPPK crystal structure (Fig. S18†). The carboxyl group of D107 could conceivably be interacting with either the hydroxyl pendants of the tag or directly with the lanthanide ion to influence the charge of the chelate and its paramagnetic properties. In addition, HPPK samples tagged with **C7** and **C8** appeared more stable to precipitation than those tagged with **C1** or **C2**, allowing the acquisition of multiple NMR experiments of each sample.

## Experimental

### Materials and methods

(1*S*,4*S*,7*S*)-1,4,7-Tris(2-hydroxypropyl)-1,4,7,10-tetraazacyclododecane,^46^ 2-chloro-*N*-(2-(pyridin-2-yldisulfanyl)ethyl)acetamide^28^ and dimethyl 4-(hydroxymethyl)pyridine-2,6-dicarboxylate^23^ were prepared following literature procedures. The synthesis of **C5** and **C6** is described in the ESI.†

### Synthetic procedures

#### (*S*)-1-Chloro-3-(tritylthio)propan-2-ol (**10**)

Triphenylmethanethiol (2.242 g, 8.11 mmol) was added to a solution of (*S*)-epichlorohydrin (500 mg, 5.40 mmol) and potassium fluoride (628 mg, 10.81 mmol) in MeOH (15 mL) and the mixture was stirred vigorously at room temperature for 72 h. Insoluble material was removed by filtration and the filtrate concentrated under reduced pressure. The resulting residue was washed with H_2_O (10 mL) and Et_2_O (10 mL) and the aqueous layer washed twice more with Et_2_O (10 mL each). The organic layers were combined, dried with anhydrous MgSO_4_ and concentrated under reduced pressure. The resulting oil was purified by silica flash chromatography (10% EtOAc in PET Spirits) to yield **10** as a colourless oil. Yield: 1.888 g (95%). ^1^H NMR (400 MHz, MeOD) *δ* 7.41 (m, 6H), 7.29 (m, 6H), 7.22 (m, 3H), 3.41 (m, 3H, C*H*OH, C*H*_2_Cl), 2.41 (m, 2H, C*H*_2_S). ^13^C NMR (101 MHz, MeOD) *δ* 146.13 (*C*), 130.78 (*C*H), 128.94 (*C*H), 127.84 (*C*H), 71.33 (*C*HOH), 67.62 (*C*(Ph)_3_), 49.032 (*C*H_2_Cl), 36.96 (*C*H_2_S). *R*_f_ (10% EtOAc in PET Spirits): 0.19.

#### (2*S*,2′*S*,2′′*S*)-1,1′,1′′-(10-((*R*)-2-Hydroxy-3-(tritylthio)propyl)-1,4,7,10-tetraazacyclododecane-1,4,7-triyl)tris(propan-2-ol) (**11**)

Potassium carbonate (601 mg, 4.35 mmol) was added to a solution of (1*S*,4*S*,7*S*)-1,4,7-tris(2-hydroxypropyl)-1,4,7,10-tetraazacyclododecane (300 mg, 0.87 mmol) and **10** (321 mg, 0.87 mmol) in ACN (5 mL). The mixture was heated to reflux for 20 h, after which an additional equivalent of **10** (321 mg, 0.87 mmol) was added and refluxed for a further 4 h. After cooling to room temperature, insoluble salts were removed by filtration and the filtrate concentrated under reduced pressure. 1 M NaOH (25 mL) was added to the residue and washed with CHCl_3_ (3 × 25 mL). The organic layers were combined, dried with anhydrous MgSO_4_ and concentrated under reduced pressure. The resulting residue was purified by silica flash chromatography (0–10% MeOH, 1% NH_3_ in CHCl_3_) to yield **11** as a yellow oil. Yield: 357 mg (60%). ^1^H NMR (400 MHz, CDCl_3_) *δ* 7.41 (m, 6H), 7.26 (m, 6H), 7.18 (m, 3H), 5.28 (br, 1H), 4.97 (br, 1H), 3.86 (m, 3H, C*H*CH_3_), 3.46 (m, 1H, C*H*CH_2_S), 2.95–2.73 (m, 8H), 2.45 (dd, *J* = 12.4, 5.2 Hz, 1H), 2.37–2.16 (m, 6H), 2.11 (dd, *J* = 4.5, 1.6 Hz, 1H), 2.08 (dd, *J* = 4.4, 1.7 Hz, 1H), 2.01 (m, 7H), 1.96–1.89 (m, 2H), 1.08 (d, *J* = 6.3 Hz, 3H, C*H*_3_), 1.06 (d, *J* = 6.2 Hz, 6H, C*H*_3_). ^13^C NMR (101 MHz, CDCl_3_) *δ* 144.93 (*C*), 129.76 (*C*H), 127.93 (*C*H), 126.64 (*C*H), 66.72 (*C*(Ph_3_)), 66.19 (*C*HOH), 63.72 (*C*H_2_), 63.12 (*C*H_2_), 63.02 (*C*HOH), 62.86 (*C*HOH), 61.87, 51.50, 51.44, 51.11, 51.00 (previous 5 signals *C*H_2_), 36.30 (*C*H_2_S), 20.09 (*C*H_3_), 20.00 (*C*H_3_). LC-MS: *m*/*z* (ESI, 20 V) 436.3 (100%) [M + 2H-trityl]^+^, 679.4 (28%) [M + H]^+^.

#### (2*S*,2′*S*,2′′*S*)-1,1′,1′′-(10-((*R*)-2-Hydroxy-3-(pyridin-2-yldisulfanyl)propyl)-1,4,7,10-tetraazacyclododecane-1,4,7-triyl)tris(propan-2-ol), trifluoroacetate salt (**C7**)

Trifluoroacetic acid (1 mL) was added slowly to a solution of **11** (350 mg, 0.52 mmol) and triethylsilane (124 μL, 0.77 mmol) in DCM (2 mL), forming a cloudy mixture that was stirred at room temperature for 1 h. Volatile reagents were removed by blowing a stream of N_2_ over the open reaction vessel, before further concentrating under reduced pressure. The resulting residue was dissolved in MeOH (5 mL) and DCM (1 mL), before 2,2′-dipyridyldisulfide (229 mg, 1.04 mmol) was added and the solution stirred at room temperature for 15 min before concentrating under reduced pressure. The residue was washed between 0.1% TFA in H_2_O (15 mL) and DCM (15 mL) and the aqueous layer purified by reverse-phase HPLC (0.1% TFA and a 5–100% ACN gradient over 20 min on a C18 preparative column). Fractions containing pure product were lyophilised to yield the trifluoroacetate salt of **C7** as a yellow oil. Yield: 208 mg (39%, assuming a pentatrifluoroacetate salt). ^1^H NMR (400 MHz, D_2_O) *δ* 8.56 (m, 1H, *H*6 of Pyr), 8.30 (ddd, *J* = 8.4, 7.7, 1.6 Hz, 1H, *H*4 of Pyr), 8.13 (d, *J* = 8.4 Hz, 1H, *H*3 of Pyr), 7.70 (m, 1H, *H*5 of Pyr), 4.25–4.04 (m, 4H, C*H*OH), 3.62–3.42 (m, 4H), 3.34–2.96 (m, 14H), 2.90–2.55 (m, 8H), 1.14 (d, *J* = 6.1 Hz, 6H, C*H*_3_), 1.08 (d, *J* = 6.3 Hz, 3H, C*H*_3_). ^13^C NMR (101 MHz, D_2_O) *δ* 155.55 (*C*2 of Pyr), 145.40 (*C*4 of Pyr), 142.73 (*C*6 of Pyr), 125.44 (*C*3 of Pyr), 124.03 (*C*5 of Pyr), 64.94 (*C*HOH), 62.71 (*C*HOH), 60.82 (*C*HOH), 59.82, 59.74, 56.75, 50.49, 50.35, 49.76, 49.27 (previous 7 signals *C*H_2_), 43.51 (*C*H_2_S), 20.27 (*C*H_3_), 19.78 (*C*H_3_), 19.75 (*C*H_3_). HRMS (ESI) *m*/*z* calcd [M + H]^+^ C_25_H_48_N_5_O_4_S_2_: 546.3142, found: 546.3140. Analytical HPLC: *t*_R_ 4.21 min, 98% (254 nm).

### Formation of lanthanide complexes

Complexes of **C5** and **C6** were prepared by refluxing the ligands for 18 h with 2 equivalents of Y^3+^, Dy^3+^, Tb^3+^, Tm^3+^ or Yb^3+^-trichloride salts in a 1 : 1 ACN : H_2_O solution adjusted to neutral pH, followed by purification by HPLC (0.1% TFA and a 0–80% ACN gradient on a C18 preparative column) to afford the complexes as off-white solids after lyophilisation. In the case of **C6**, TCEP was added prior to purification to prevent disulfide formation.

Complexes of **C7** and **C8** were most readily prepared from **11**. An example of the formation of the **C7-Yb^3+^** complex follows. A solution of **11** (30 mg, 0.044 mmol) and YbCl_3_ (19 mg, 0.066 mmol) in MeOH (1.5 mL) was heated at 50 °C for 4 h, after which LCMS analysis indicated complete complexation. The solution was cooled to room temperature, then 2,2′-dipyridyldisulfide (29 mg, 0.13 mmol) and silver nitrate (37 mg, 0.22 mmol) added whilst vigorously stirring, forming a milky beige mixture, before formation of a beige precipitate that eventually turned grey. After 2 h, LCMS analysis indicated complete trityl deprotection and thiol activation, and the mixture was concentrated under reduced pressure. 0.1% TFA in H_2_O (5 mL) and DCM (5 mL) were added to the grey residue and the suspension transferred to a 15 mL centrifuge tube. The suspension was shaken vigorously and the precipitate sedimented and organic and aqueous phases separated, by centrifugation for 3 min at 2000 rcf. The aqueous phase was carefully removed and purified by reverse-phase HPLC (0.1% TFA and a 5–100% ACN gradient over 30 min on a C18 preparative column). Fractions containing pure product were lyophilised to yield the trifluoroacetate salt of **C7-Yb^3+^** as an off-white solid. Yield: 17 mg (34%, assuming a tetratrifluoroacetate salt).

Working stock solutions of each metal complex were prepared at 20 mM in H_2_O and stored frozen at –20 °C when not in use.

#### **C5-Y^3+^**

^1^H NMR (400 MHz, D_2_O) *δ* 8.56 (m, 1H), 8.18 (m, 1H), 8.08 (m, 1H), 7.60 (ddd, *J* = 7.5, 5.5, 1.1 Hz, 1H), 4.71 (m, 1H), 4.56 (m, 2H), 4.03 (d, *J* = 16.3 Hz, 1H), 3.94 (m, 1H), 3.61–3.29 (m, 9H), 3.21–3.04 (m, 4H), 2.82 (m, 1H), 2.74–2.28 (m, 12H), 1.36 (d, *J* = 5.9 Hz, 3H), 1.28 (d, *J* = 5.9 Hz, 3H), 1.24 (d, *J* = 5.8 Hz, 3H). HRMS (ESI) *m*/*z* calcd [M – 2H]^+^ C_26_H_46_N_6_O_4_S_2_Y: 659.2091, found: 659.2087.

#### **C6-Y^3+^**

^1^H NMR (400 MHz, D_2_O) *δ* 8.02 (s, 1H), 7.76 (s, 1H), 4.55–4.34 (m, 3H), 3.91 (s, 2H), 3.81 (d, *J* = 15.0 Hz, 1H), 3.73–3.46 (m, Hz, 7H), 3.24–3.05 (m, 4H), 2.66 (d, *J* = 13.1 Hz, 2H), 2.58–2.35 (m, 6H), 2.32–2.15 (m, 4H), 1.43 (d, *J* = 5.8 Hz, 3H), 1.24 (d, *J* = 5.7 Hz, 3H), 0.88 (d, *J* = 5.7 Hz, 3H). HRMS (ESI) *m*/*z* calcd [M – 2H]^+^ C_22_H_43_N_5_O_5_SY: 614.2043, found: 614.2046.

#### **C7-Y^3+^**

^1^H NMR (400 MHz, D_2_O) *δ* 8.38 (t, *J* = 4.5 Hz, 1H), 7.90–7.74 (m, 2H), 7.31 (m, 1H), 4.61–4.44 (m, 2H), 4.21–4.01 (m, 2H), 3.59–2.57 (m, 20H), 2.44–2.10 (m, 6H), 1.20 (m, 5H), 1.13 (d, *J* = 6.1 Hz, 2H), 1.08 (d, *J* = 6.1 Hz, 2H). HRMS (ESI) *m*/*z* calcd [M – 2H]^+^ C_25_H_45_N_5_O_4_S_2_Y: 632.1917, found: 632.1963.

^1^H NMR spectra and HRMS of the Yb^3+^ complexes of **C5–C7**, are shown in Fig. S1–S6.†

### NMR sample preparation

Uniformly ^15^N-labelled human ubiquitin A28C was prepared as described.^24^ Prior to tagging the protein was first reduced by stirring with a 10-fold excess of DTT for 1 h at room temperature, before passage over a PD-10 column equilibrated with degassed buffer (50 mM HEPES, pH 8).

For the **C5** and **C7/8** tags a 5-fold excess of the respective lanthanide complex was added to a solution of protein and stirred at room temperature for 2 h. Excess tag was removed by passage over a PD-10 column before the sample was concentrated using a Millipore ultrafilter (3 kDa) to a final protein concentration of approximately 100 μM.

In order to tag **C6**, the protein cysteines were first pre-activated by addition of 10 equivalents of 5,5′-dithiobis-2-nitrobenzoic acid (DTNB), producing a yellow coloured solution that was allowed to stir at room temperature for 1 h. Excess DTNB and TNB^2–^ leaving group were removed by passage through a PD10 column, yielding a colourless solution. A 5-fold excess of the respective **C6** complex was then added, forming a yellow solution that was stirred at room temperature for 2 h. Excess tag and TNB^2–^ leaving group was removed by passage over a PD-10 column and samples were concentrated as above.

### NMR spectroscopy

Spectra of differently tagged ubiquitin A28C in 90%/10% H_2_O/D_2_O, 50 mM HEPES, pH 8.0, were recorded at 25 °C on either Varian INOVA or Bruker Avance 600 MHz NMR spectrometers equipped with cryogenic probes. ^1^H^N^ PCSs and ^1^ *D*_HN_ couplings were measured by recording ^15^N-fast-HSQC spectra with and without the 180° (^1^H) pulse during the ^15^N (*t*_1_) evolution time. 2D ^15^N-fast-HSQC were typically acquired with *t*_1max_ (^15^N) = 51–62 ms and *t*_2max_ (^1^H) = 142 ms.

### Calculation of Δ*χ* and alignment tensors

Fitting of Δ*χ*-tensors was carried out within the program Numbat.^53^ The tensors were fitted to the first conformer of the NMR structure of ubiquitin (PDB ; 2MJB^47^). Unambiguous PCS assignments were used to calculate an initial estimate of the Δ*χ*-tensor, from which iterative cycles of further assignment and recalculation were made. The Δ*χ*-tensors for GB1 and HPPK were determined in an analogous way, fitting to the crystal structures of GB1 (PDB ; 1PGA^54^) and HPPK (PDB ; 3QBC^45^).

Backbone amide ^1^ *D*_HN_ RDCs were fitted to the first conformer of the NMR structure of ubiquitin (PDB ; 2MJB^47^) using single value decomposition *via* the “-bestFit” flag in PALES.^51^

## Conclusions

We have presented the synthesis of three new LBT designs. Each tag is capable of binding lanthanide ions tightly and producing significant PCSs without need for the addition of free paramagnetic metal ions to protein samples. Each design features hydroxypropyl pendant arms, rendering the tags smaller and more hydrophilic than previously reported DOTA-style tags. The **C5** tag can be readily synthesised and displayed comparable paramagnetic effects to **C1**, whose utility has been proven in several studies.^55–57^ The **C6** tag also performed comparably with **C1** on ubiquitin, however it features the longest synthesis of any of the tags and requires more protein handling *via* DTNB activation for conjugation. The **C7/8** design features a particularly short linker, resulting in limited mobility relative to the protein surface, hence generating the largest paramagnetic effects on ubiquitin. The capability of **C7** and **C8** to produce paramagnetic effects on other proteins was further demonstrated on GB1 and HPPK. The performance of the tags varied with factors including the lanthanide used, pH and site of conjugation. Given their favourable properties, it is anticipated that **C7** and **C8** (particularly their Tm^3+^ complexes) will prove useful in the investigation of a wide range of biologically interesting proteins by paramagnetic NMR spectroscopy.
