# Supplementary material for: Compact, hydrophilic, lanthanide-binding tags for paramagnetic NMR spectroscopy
Source: Chem Sci. 2015 Feb 25;6(4):2614–24. doi: 10.1039/c4sc03892d (PMC5812434; doi:10.1039/c4sc03892d)
Supplement: Supplementary file 1 [file SC-006-C4SC03892D-s001.pdf]

Electronic Supplementary Material (ESI) for Chemical Science.
This journal is © The Royal Society of Chemistry 2015

Supplementary Information

Compact, hydrophilic, lanthanide-binding tags for paramagnetic NMR spectroscopy

M. D. Lee,[a] C.-T. Loh,[b] J. Shin,[a] S. Chhabra,[a] M. L. Dennis,[a] G. Otting,[b] J. D. Swarbrick[a]* and B. Graham[a]*

Contents

Syntheses of **C5** and **C6**........ S2
^{1}H NMR spectra of Yb^{3+} complexes of **C5**–**C7**........ S4
High-resolution mass spectra of Yb^{3+} complexes of **C5**–**C7**........ S6
GB1 expression, purification and tagging........ S8
HPPK expression, purification and tagging........ S8
^{15}N-HSQC spectra of **C5**–**C8** tagged ubiquitin A28C........ S9
Experimental PCSs measured for **C5**–**C8** tagged ubiquitin A28C........ S12
$\Delta\chi$-Tensor parameters for **C5**–**C8** tagged ubiquitin A28C (common fits)........ S16
^{15}N-HSQC spectra of **C7** tagged ubiquitin A28C at pH 6.5........ S17
Correlations between PCSs of **C7** tagged ubiquitin A28C at pH 8.0 and pH 6.5........ S17
Plots of measured PCSs *vs.* residue number for **C5**–**C8** tagged ubiquitin A28C........ S18
Comparison of $\Delta\chi$-tensor orientations and individual metal positions determined for different metal complexes of **C1**, **C7** and **C8** bound to ubiquitin A28C........ S19
Temperature titration of **C7** tagged ubiquitin A28C........ S20
Experimental $^{1}D_{HN}$ RDCs for **C7** and **C8** tagged ubiquitin A28C loaded with Tm^{3+}........ S21
Alignment tensor parameters for **C7** and **C8** tagged ubiquitin A28C loaded with Tm^{3+}........ S22
Equation S1........ S22
^{15}N-HSQC spectra of **C7** and **C8** tagged GB1 Q32C........ S23
Correlations between experimental and back-calculated PCSs for **C7** and **C8** tagged GB1 Q32C and **C7** tagged HPPK S112C/C80A........ S24
$\Delta\chi$-Tensor parameters for **C7** and **C8** tagged GB1 Q32C (common fits)........ S24
Experimental PCSs measured for **C7** and **C8** tagged GB1 Q32C........ S25
^{15}N-HSQC spectra of **C7** tagged HPPK S112C/C80A........ S27
Structure of HPPK and PCS-determined metal ion position for **C7** tagged HPPK S112C/C80A loaded with Tm^{3+}........ S27
Experimental PCSs measured for **C7** tagged HPPK S112C/C80A loaded with Tm^{3+}........ S28
^{1}H and ^{13}C NMR spectra of novel compounds........ S29
Analytical HPLC traces of new lanthanide-binding tags........ S43
References........ S44

Syntheses of C5 and C6

***N*-(2-(pyridin-2-yldisulfanyl)ethyl)-2-(4,7,10-*tris*((*S*)-2-hydroxypropyl)-1,4,7,10-tetraazacyclododecan-1-yl)acetamide, trifluoroacetate salt (C5).** (1*S*,4*S*,7*S*)-1,4,7-*tris*(2-hydroxypropyl)-1,4,7,10-tetraazacyclododecane (468 mg, 1.35 mmol), 2-chloro-N-(2-(pyridin-2-yldisulfanyl)ethyl)acetamide (532 mg, 2.02 mmol) and DIPEA (352 µL, 2.02 mmol) were dissolved in ACN (8 mL) and stirred at room temperature for 72 h. The solution was concentrated under reduced pressure and the resulting residue purified by reverse-phase HPLC (0.1% TFA and a 5–50% ACN gradient on a C18 preparative column). Fractions containing pure product were lyophilised to yield the trifluoroacetate salt of **C5** as a yellow oil. Yield: 792 mg (61 %, assuming a pentatrifluoroacetate salt). ^{1}H NMR (400 MHz, D_2O) δ 8.60 (m, 1H), 8.37 (m, 1H), 8.20 (d, *J* = 8.4 Hz, 1H), 7.76 (m, 1H), 4.18 (br, 2H, C*H*OH), 4.06 (m, 1H, C*H*OH), 3.68-3.43 (m, 7H), 3.39 – 3.11 (m, 12H), 3.06 (br, 2H), 2.98 (m, 3H), 2.79 (m, 2H), 2.54 (m, 2H), 1.17 (br, 6H, C*H*$_3$), 1.08 (d, *J* = 6.3 Hz, 3H, C*H*$_3$). ^{13}C NMR (101 MHz, D_2O) δ 172.27 (*C*=O), 156.11 (*C*), 144.35 (*C*H), 143.86 (*C*H), 124.66 (*C*H), 123.66 (*C*H), 63.90 (*C*HOH), 61.05 (*C*HOH), 60.69 (*C*HOH), 60.56, 60.24, 59.49, 54.79, 51.42, 50.65, 50.27, 50.04, 49.01, 46.62, 46.15, 37.72, 37.46, 36.33 (previous 14 peaks, *C*H$_2$), 20.12 (*C*H$_3$), 20.01 (*C*H$_3$), 19.95 (*C*H$_3$). HRMS (ESI) *m/z* cal'd for $[M+H]^+$ $C_{26}H_{49}N_6O_4S_2$: 573.3251, found: 573.3258. Analytical HPLC: t_R 4.27 min, 97% (254 nm).

Dimethyl 4-(((methylsulfonyl)oxy)methyl)pyridine-2,6-dicarboxylate (4). Dimethyl 4-(hydroxymethyl)pyridine-2,6-dicarboxylate (638 mg, 2.83 mmol) and DIPEA (1480 µL, 8.49 mmol) was dissolved in anhydrous DCM (55 mL). Methanesulfonyl chloride (330 µL, 4.25 mmol) was added dropwise to the solution over an ice bath. After complete addition, the solution was allowed to warm to room temperature and stirred for 30 min. The solution was washed with H_2O (2 x 50 mL) and the organic layer was dried with anhydrous $MgSO_4$ and concentrated under reduced pressure to yield **4** as a white solid that was used without further purification. Yield: 864 mg (quant). ^{1}H NMR (400 MHz, $CDCl_3$) δ 8.31 (s, 2H, *H*3, *H*5), 5.36 (s, 2H, C*H*$_2$), 4.04 (s, 6H, OC*H*$_3$), 3.14 (s, 3H, SC*H*$_3$). ^{13}C NMR (101 MHz, $CDCl_3$) δ 164.77 (*C*=O), 149.07 (*C*2, *C6*), 146.10 (*C*4), 126.09 (*C*3, *C*5), 67.18 (*C*H$_2$), 53.57 (O*C*H$_3$), 38.30 (S*C*H$_3$). LC-MS: *m/z* (ESI, 20 V) 304.1 (100%) $[M+H]^+$.

Dimethyl 4-((*tert*-butylthio)methyl)pyridine-2,6-dicarboxylate (5). *Tert*-butylthiol (410 µL, 3.54 mmol) was added dropwise to a mixture of NaH (60% in mineral oil, 141 mg, 3.54mmol) in DMF (5 mL). The thiolate solution was then added dropwise to a solution of **4** (858 mg, 2.83 mmol) in DMF (7 mL). The solution was stirred for 5 min at room temperature. Et_2O (100 mL) was added and the solution washed with H_2O (2 x 100 mL). The combined aqueous phase was washed with Et_2O (50 mL) and the combined organic layers dried with anhydrous $MgSO_4$ and filtered before concentrating by evaporating under a gentle N_2 flow. The resulting residue was purified by silica flash chromatography (30% EtOAc in PET Spirits) to yield **5** as a white solid. Yield: 399 mg (47%). ^{1}H NMR (400 MHz, $CDCl_3$) δ 8.27 (s, 2H, *H*3, *H*5), 3.98 (s, 6H, OC*H*$_3$), 3.81 (s, 2H, C*H*$_2$), 1.30 (s, 9H, C(C*H*$_3$)$_3$). ^{13}C NMR (101 MHz, $CDCl_3$) δ 165.11 (*C*=O), 151.97 (*C*4), 148.44 (*C*2, *C*6), 128.41 (*C*3, *C*5), 53.26 (O*C*H$_3$), 43.91 (*C*(CH$_3$)$_3$), 32.35 (*C*H$_2$), 30.94 (C(*C*H$_3$)$_3$). LC-MS: *m/z* (ESI, 20 V) 298.2 (100%) $[M+H]^+$. R_f(30% EtOAc in PET Spirits): 0.28.

Methyl 4-((*tert*-butylthio)methyl)-6-(hydroxymethyl)picolinate (6). Sodium borohydride (104 mg, 2.74 mmol) was added slowly to a stirring solution of **5** (678 mg, 2.28 mmol) in MeOH (60 mL) and DCM (20 mL) over an ice bath. The solution was allowed to warm to room temperature. Further portions of sodium borohydride (50 mg, 1.32 mmol each) were added after 1 and 1.5 h. After 2 h the reaction was concentrated under reduced pressure. EtOAc (50 mL) was added to the resulting residue and washed with H_2O (2 x 50 mL). The organic layer was dried with anhydrous $MgSO_4$ and concentrated under reduced pressure. The resulting oil was purified by silica flash chromatography (40% EtOAc in PET Spirits) to yield **6** as a white solid. Yield: 395 mg (64%). ^{1}H NMR (400 MHz, $CDCl_3$) δ 8.02 (s, 2H, *H*3), 7.45 (s, 1H, *H*5), 4.83 (s, 2H, OC*H*$_2$), 3.98 (s, 3H, OC*H*$_3$), 3.77 (s, 2H, SC*H*$_2$), 1.33 (s, 9H, C(C*H*$_3$)$_3$). ^{13}C NMR (101 MHz, $CDCl_3$) δ 165.48 (*C*=O), 160.60 (*C*6), 151.05 (*C*4), 147.10 (*C*2), 124.57 (*C*3), 124.42 (*C*5), 64.61 (O*C*H$_2$), 53.05 (O*C*H$_3$), 43.80 (*C*(CH$_3$)$_3$, 32.52 (S*C*H$_2$), 31.00 (C(*C*H$_3$)$_3$). LC-MS: *m/z* (ESI, 20 V) 270.2 (100%) $[M+H]^+$. R_f (40% EtOAc in PET Spirits): 0.14.

Methyl 4-((*tert*-butylthio)methyl)-6-(((methylsulfonyl)oxy)methyl)picolinate (7). Compound **6** (402 mg, 1.49 mmol) and DIPEA (779 µL, 4.47 mmol) was dissolved in anhydrous DCM (20 mL). Methanesulfonyl chloride (231 µL, 2.98 mmol) was added dropwise to the solution over an ice bath. After complete addition, the solution was allowed to warm to room temperature and stirred for 15 min. The solution was washed with H_2O (2 x 20 mL) and the organic layer was

dried with anhydrous $MgSO_4$ and concentrated under reduced pressure. The resulting oil was purified by silica flash chromatography (40% EtOAc in PET Spirits) to yield **7** as an orange oil. Yield: 401 mg (77%). ^{1}H NMR (400 MHz, $CDCl_3$) δ 8.09 (s, 1H, *H*3), 7.64 (s, 1H, *H*5), 5.38 (s, 2H, CH_2OS), 3.97 (s, 3H, OCH_3), 3.78 (s, 2H, CH_2S), 3.12 (s, 3H, SCH_3), 1.32 (s, 9H, $C(CH_3)_3$).^{13}C NMR (101 MHz, $CDCl_3$) δ 165.20 (*C*=O), 154.59 (*C*6), 151.57 (*C*4), 147.97 (*C*2), 125.54 (*C*3, *C*5), 71.03 (*C*H_2OS), 53.12 (O*C*H_3), 43.85 (*C*$(CH_3)_3$), 38.13 (S*C*H_3), 32.40 (*C*H_2S), 30.94 ($C(CH_3)_3$).LC-MS: *m/z* (ESI, 20 V) 348.1 (100%) $[M+H]^+$. R_f (40% EtOAc in PET Spirits): 0.28.

Methyl 6-((1,4,7,10-tetraazacyclododecan-1-yl)methyl)-4-((*tert*-butylthio)methyl)picolinate (8). Compound **7** (451 mg, 1.30 mmol) dissolved in $CHCl_3$ (30 mL) was added dropwise to a solution of 1,4,7,10-tetraazacyclododecane (1.344 g, 7.80 mmol) in $CHCl_3$ (100 mL) and stirred overnight at room temperature. The solution was washed with 1 M NaOH (1 x 100 mL) and H_2O (3 x 100 mL) to remove excess 1,4,7,10-tetraazacyclododecane. The organic layer was dried with anhydrous $MgSO_4$ and concentrated under reduced pressure to yield **8** as a colourless oil. Yield: 561 mg (quantitative). ^{1}H NMR (400 MHz, $CDCl_3$) δ 7.85 (d, *J* = 1.4 Hz, 1H, *H*3), 7.64 (d, *J* = 1.4 Hz, 1H, *H*5), 3.84 (s, 3H, OCH_3), 3.73 (s, 2H, NCH_2Ar), 3.64 (s, 2H, SCH_2), 2.73 – 2.67 (m, 4H), 2.54 (m, 8H), 2.48 – 2.44 (m, 4H), 1.20 (s, 9H, $C(CH_3)_3$).^{13}C NMR (101 MHz, $CDCl_3$) δ 165.59 (*C*=O), 160.79 (*C*6), 149.95 (*C*4), 147.12 (*C*2), 126.36 (*C*5), 124.15 (*C*3), 60.66 (N*C*H_2Ar), 52.69 (O*C*H_3), 51.81, 47.03, 46.33, 45.09, 43.25 (*C*$(CH_3)_3$), 32.31 (S*C*H_2), 30.78 ($C(CH_3)_3$). LC-MS: *m/z* (ESI, 20 V) 368.20 $[M-^tBu+H]^+$ (100%), 424.40 $[M+H]^+$ (66%).

Methyl 4-((*tert*-butylthio)methyl)-6-((4,7,10-*tris*((S)-2-hydroxypropyl)-1,4,7,10-tetraazacyclododecan-1-yl)methyl)picolinate (9). Compound **8** (550 mg, 1.30 mmol) was dissolved in MeOH (30 mL) and (*S*)-propylene oxide (546 µL, 7.80 mmol) was added. The solution was stirred at room temperature for 48 h. Solvent and excess (*S*)-propylene oxide was removed under reduced pressure to yield **9** as colourless oil that was used without further purification. Yield: 777 mg (quant.). ^{1}H NMR (400 MHz, $CDCl_3$) δ 7.98 (d, *J* = 1.2 Hz, 1H, *H*3), 7.45 (d, *J* = 1.2 Hz, 1H, *H*5), 5.02 (br, 2H, O*H*), 4.40 (br, 1H, O*H*), 4.08-4.04 (br, 2H, CHOH), 4.01 (d, *J* = 13.8 Hz, NCH_2Ar), 3.93 (m, 4H, OCH_3 and C*H*OH), 3.86 (d, *J* =13.3 Hz, 1H, CH_2S), 3.78 (d, *J* =13.3 Hz, 1H, CH_2S), 3.73 (d, *J* =13.9 Hz, 1H, NCH_2Ar), 3.16 (m, 2H), 2.93 (m, 2H), 2.82 (m, 4H), 2.52 (m, 1H), 2.38 (m, 1H), 2.27 – 1.80 (m, 12H), 1.31 (s, 9H, $C(CH_3)_3$), 1.23 (d, *J* = 6.1 Hz, 3H, CHCH_3), 1.12 (d, *J* = 6.1 Hz, 3H, CHCH_3), 0.90 (d, *J* = 6.1 Hz, 3H, CHCH_3).^{13}C NMR (101 MHz, $CDCl_3$) δ 165.95 (*C*=O), 157.69 (*C*6), 150.62 (*C*4), 147.55 (*C*2), 128.41 (*C*5), 124.34 (*C*3), 62.89 (*C*HCH_3), 62.83 , 62.58 (*C*HCH_3), 62.32 (*C*HCH_3), 61.80, 61.24, 59.02, 52.83 (O*C*H_3), 52.60, 51.79, 51.42, 50.24, 50.15, 50.11, 49.86, 47.45, 43.62 (*C*$(CH_3)_3$), 32.32 (*C*H_2S), 31.00 ($C(CH_3)_3$), 22.05 (CH*C*H_3), 21.77 (CH*C*H_3), 21.68 (CH*C*H_3). LC-MS: *m/z* (ESI, 20 V): 299.79 $[M+2H]^{2+}$ (100%), 598.50 $[M+H]^+$ (9%).

4-(Mercaptomethyl)-6-((4,7,10-*tris*((*S*)-2-hydroxypropyl)-1,4,7,10-tetraazacyclododecan-1-yl)methyl)picolinic acid, trifluoroacetate salt (C6). Compound **9** (184 mg, 0.31 mmol) was dissolved in HCl (32%, 10 mL) and heated to reflux for 4 h. Solvent was removed under reduced pressure and the resulting residue purified by reverse-phase HPLC (0.1% TFA and a 5–100% ACN gradient over 20 min on a C18 preparative column). Fractions containing pure product were lyophilised to afford **C6** as a white residue. Yield: 139 mg (85 %).^{1}H NMR (400 MHz, D_2O) δ 8.29 (d, *J* = 1.5 Hz, 1H, *H*3), 7.98 (d, *J* = 1.5 Hz, 1H, *H*5), 4.44 (br, 1H, C*H*OH), 4.29 (d, *J* = 14.0 Hz, 1H, NCH_2Ar), 4.23 – 4.14 (m, 1H, C*H*OH), 4.02 (br, 1H, C*H*OH), 3.95 (s, 2H, CH_2SH), 3.83 (d, *J* = 14.0 Hz, 1H, NCH_2Ar), 3.66 (m, 3H), 3.39 (m, 5H), 3.26 (m, 4H), 3.15 (d, *J* = 14.3 Hz, 1H), 3.00 (d, *J* = 12.8 Hz, 1H), 2.79 (m, 5H), 2.66 (m, 2H), 2.56 (d, *J* = 14.3 Hz, 1H), 1.35 (d, *J* = 4.5 Hz, 3H, CH_3), 1.15 (d, *J* = 6.3 Hz, 3H, CH_3), 0.91 (d, *J* = 4.6 Hz, 3H, CH_3).^{13}C NMR (101 MHz, D_2O) δ 164.72 (*C*=O), 160.05 (*C*4), 151.65 (*C*6), 147.14 (*C*2), 130.01 (*C*5), 125.43 (*C*3), 63.03 (*C*HOH), 60.62 (*C*HOH), 59.87(CH_2), 59.55 (*C*HOH), 59.31, 58.72, 55.45, 50.48, 49.62, 49.39, 48.69, 47.34, 46.46, 45.56 (previous 10 signals CH_2), 26.82 (CH_2SH), 20.43 (CH_3), 19.57 (CH_3), 19.48(CH_3). HRMS (ESI) *m/z* cal'd for $[M+H]^+$ $C_{25}H_{46}N_5O_5S$: 528.3220, found: 528.3207. Analytical HPLC: t_R 3.86 min, 97% (254 nm).

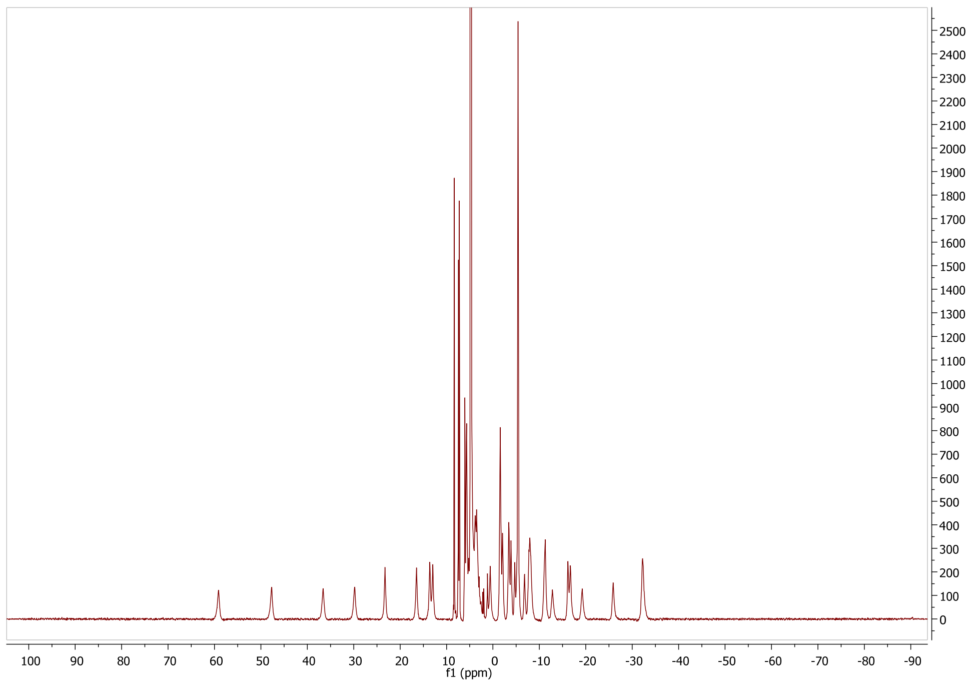

Figure S1. ^{1}H NMR spectrum of **C5-Yb^{3+}**. The spectra were recorded at 25°C and pH 2 at a ^{1}H NMR frequency of 400 MHz.

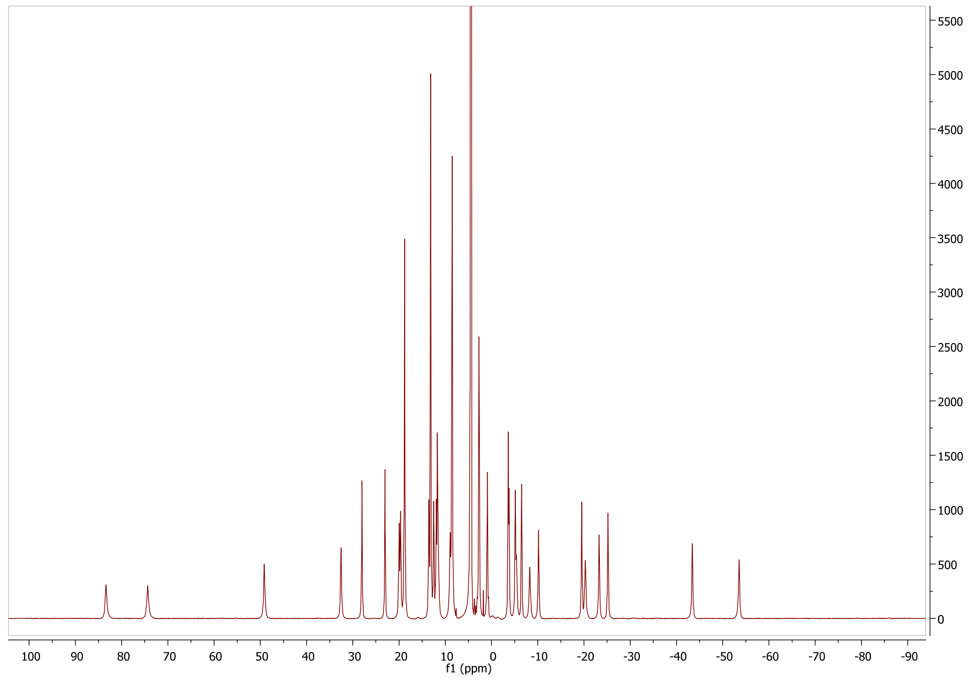

Figure S2. ^{1}H NMR spectrum of **C6-Yb^{3+}**. The spectrum was recorded at 25 °C and pH 2 at a ^{1}H NMR frequency of 400 MHz.

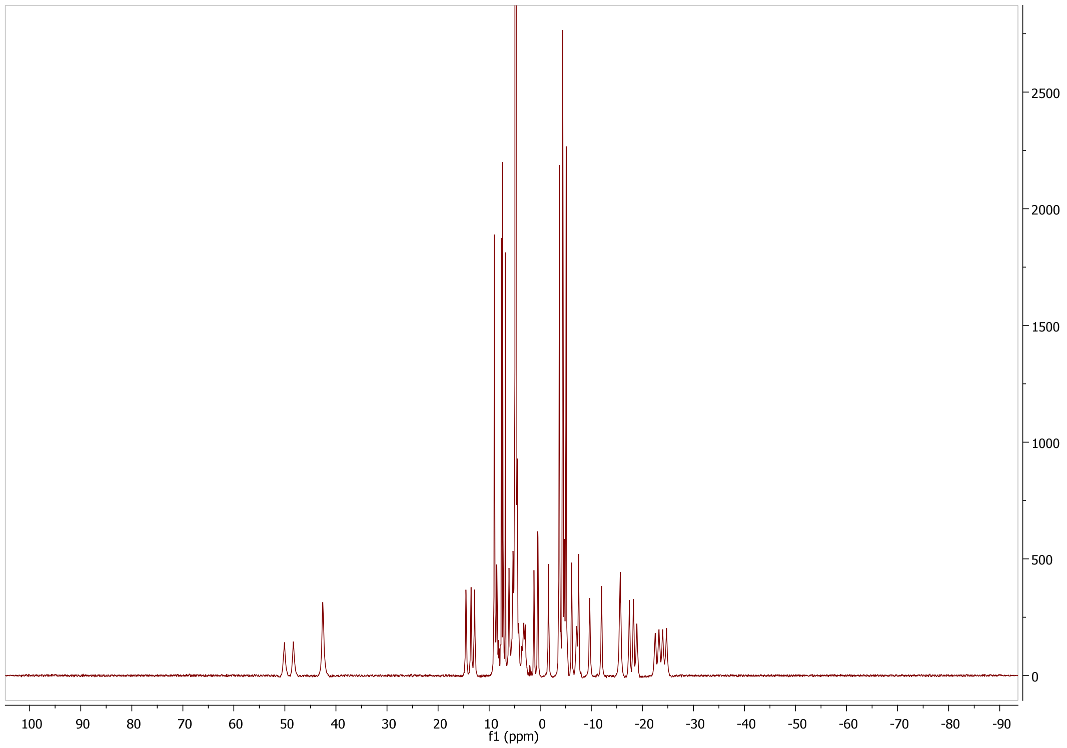

Figure S3. ^{1}H NMR spectrum of **C7-Yb^{3+}**. The spectrum was recorded at 25 °C and pH 2 at a ^{1}H NMR frequency of 400 MHz.

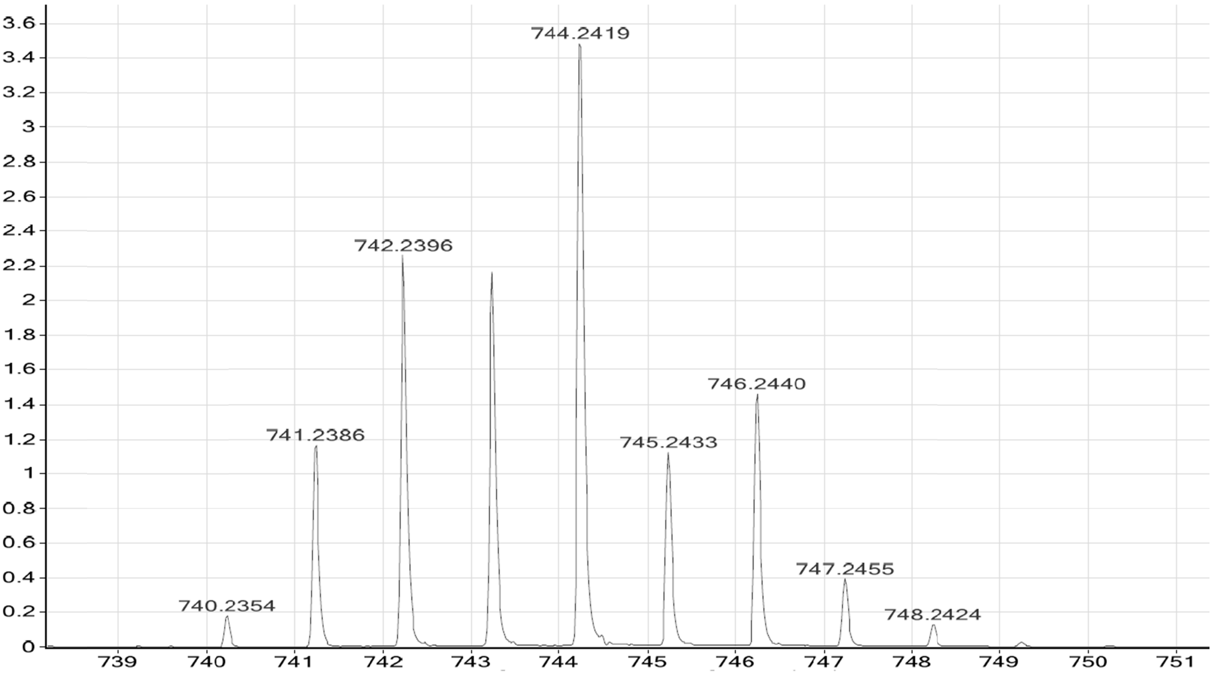

Figure S4. High-resolution mass spectrum of **C5-Yb^{3+}**.

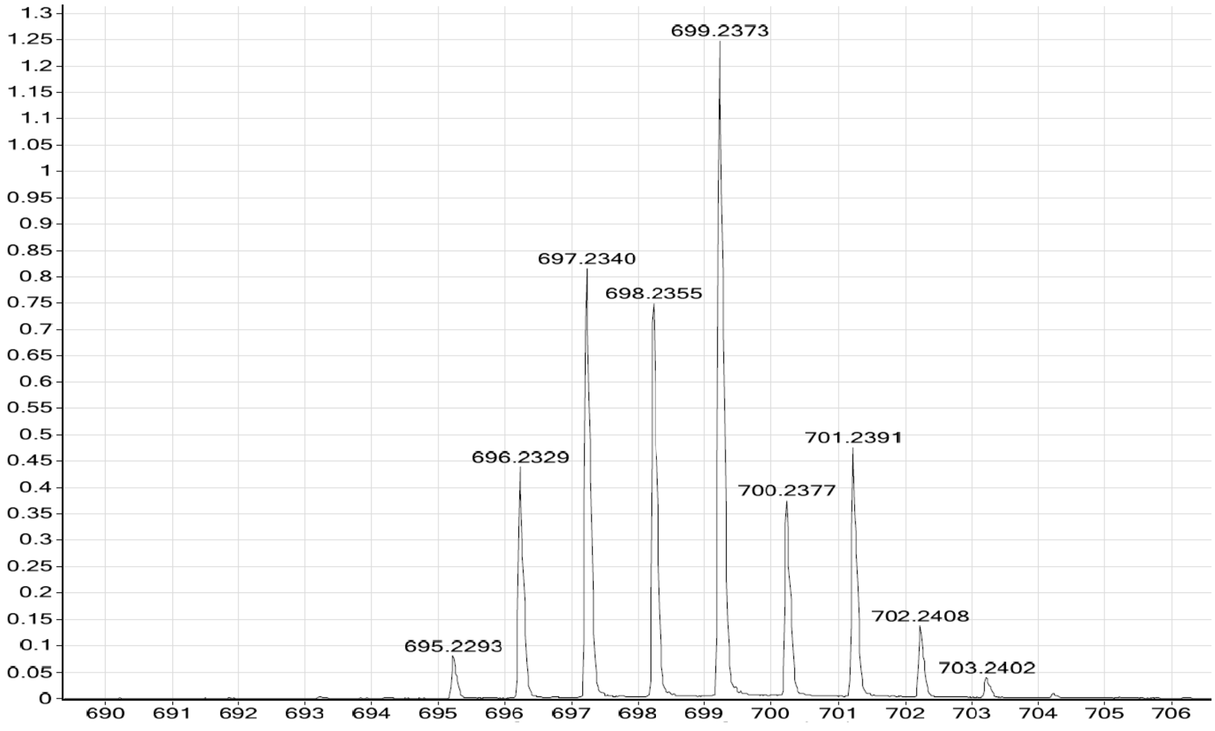

Figure S5. High-resolution mass spectrum of **C6-Yb^{3+}**.

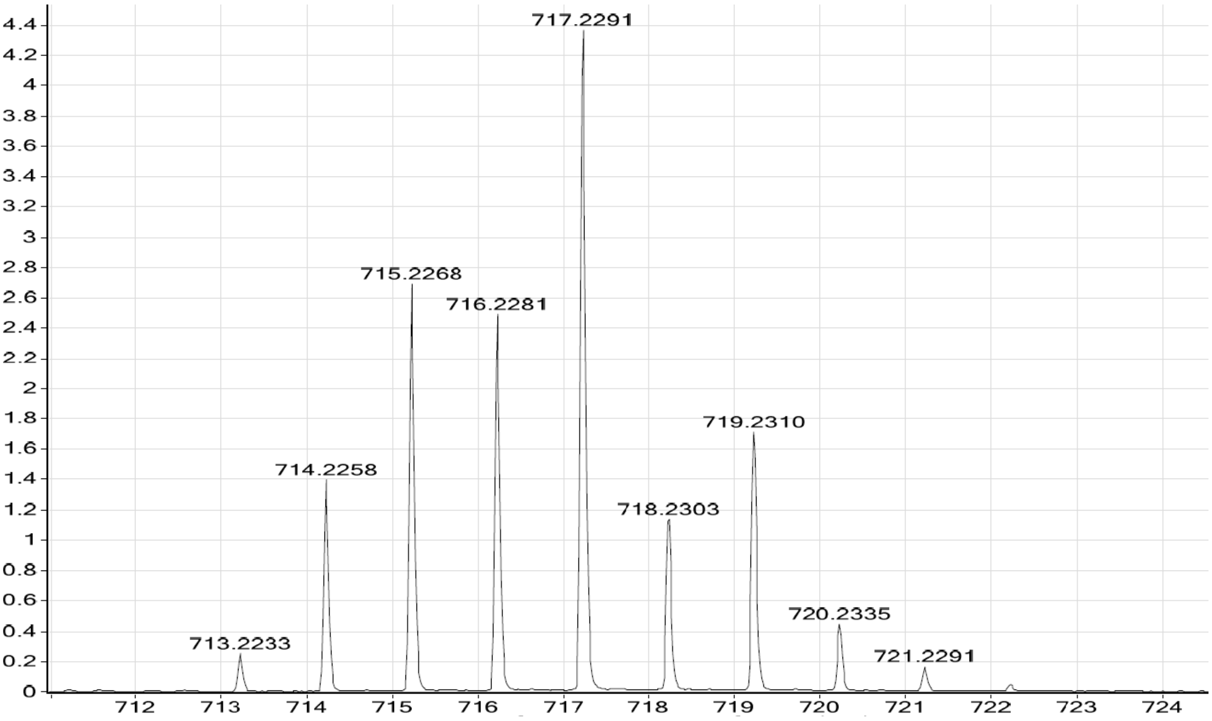

Figure S6. High-resolution mass spectrum of **C7-Yb^{3+}**.

Table S1. Predicted masses of Yb^{3+} complexes of **C5**–**C7**.

**Complex**	**Chemical formula**	**Predicted masses (relative abundance)**[a]
**C5-Yb^{3+}**	$[C_{26}H_{46}N_6O_4S_2Yb]^+$	744.2406 (100.0%), 742.2381 (68.6%), 743.2399 (50.7%), 741.2380 (44.9%), 746.2443 (40.1%), 745.2439 (28.1%), 743.2414 (19.3%), 744.2433 (14.3%), 742.2414 (12.6%), 747.2476 (11.3%)
**C6-Yb^{3+}**	$[C_{25}H_{43}N_5O_5SYb]^+$	699.2368 (100.0%), 697.2344 (68.6%), 698.2362 (50.7%), 696.2343 (44.9%), 701.2406 (40.1%), 700.2402 (27.0%), 698.2377 (18.5%), 699.2396 (13.7%), 697.2377 (12.1%), 702.2439 (10.8%)
**C7-Yb^{3+}**	$[C_{25}H_{45}N_5O_4S_2Yb]^+$	717.2297 (100.0%), 715.2272 (68.6%), 716.2290 (50.7%), 714.2271 (44.9%), 719.2334 (40.1%), 718.2330 (27.0%), 716.2305 (18.5%), 717.2324 (13.7%), 715.2305 (12.1%), 720.2367 (10.8%)
[a] Only masses of the 10 most abundant predicted species are listed.		

GB1 expression, purification and tagging

Uniformly ^{15}N-labelled GB1 Q32C was expressed with a C-terminal His_6-tag using the pETMCSI T7 vector[1] in *E. coli* BL21(DE3) grown at 37 °C overnight in the presence of 100 mg/mL ampicillin. 10 mL of overnight culture were subsequently inoculated into 1 L minimal media containing 0.5 g/L ^{15}N-ammonium chloride and 100 mg/L ampicillin. The cultures were grown at 37 °C and induced with 1 mM isopropyl-*β*-*D*-thiogalactopyranoside (IPTG) at OD_{600} 0.6. After overnight expression at room temperature (16 h), cultures were harvested by centrifugation at 5,000 *g* for 20 min. Pellets were resuspended into buffer A (20 mM Tris-HCl, pH 7.5, 150 mM NaCl, 20 mM imidazole) and lysed using a French press at 12,000 psi. Cell lysates were then centrifuged for 1 h at 34,000 *g*. The supernatant was loaded onto a 5 mL Ni-NTA column (GE Healthcare, USA) and the proteins were eluted with buffer B (same as buffer A but containing 500 mM imidazole). Fractions were analyzed by 15% SDS-PAGE. Fractions containing protein were pooled and dialyzed against 20 mM Tris-HCl, pH 7.5, and concentrated using an Amicon ultrafiltration centrifugal tube with a molecular weight cutoff of 3 kDa.

To attach **C7** or **C8** tags, GB1 Q32C was first reduced by 5 equivalents of DTT, followed by buffer exchange to 20 mM Tris-HCl, pH 7.5, to wash out DTT. The reduced protein was labelled with 5 equivalents of **C7** or **C8** tag loaded with Tb^{3+}, Tm^{3+} or Y^{3+} in 20 mM Tris-HCl, pH 7.5, and left at room temperature overnight. Excess tag was washed out using NMR buffer (20 mM MES, pH 6.5). The final protein concentration was 0.1 mM.

^{15}N-HSQC spectra of differently tagged GB1 Q32C were recorded at 25 °C using t_{1max} (^{15}N) = 43 ms and t_{2max} (^{1}H) = 136 ms.

HPPK expression, purification and tagging

The HPPK S112C/C80A mutant was created by the QuickChange (Stratagene (La JollA), CA, USA) method using pET28a-HPPK vector as a template. Uniformly ^{15}N-labeled HPPK S112C/A80C was expressed and purified following established protocols for the wild-type protein.[2,3]

To attach **C7** to HPPK S112C/C80A, DTT in the HPPK storage buffer was removed by passage over a PD-10 column equilibrated with degassed buffer (50 mM HEPES, pH 8). The eluate was made to 10 mM $MgCl_2$ and 1 mM α,β-methyleneadenosine 5'-triphosphate. **C7** loaded with either Tm^{3+} or Y^{3+} was added in 3 fold excess and the reaction stirred at room temperature for 15 min. Excess tag was removed by passage over a PD-10 column (50 mM HEPES, pH 8) and the eluate again made to 10 mM $MgCl_2$ and 1 mM α,β-methyleneadenosine 5'-triphosphate. The sample was then concentrated in an Amicon ultrafiltration centrifugal tube with a molecular weight cutoff of 3 kDa to a final protein concentration of approximately 100 µM. Prior to NMR measurements, 400 µM of a small molecule inhibitor was added to the sample.

^{15}N-HSQC spectra of differently tagged HPPK S112C/C80A were recorded at 22 °C using t_{1max} (^{15}N) = 56 ms and t_{2max} (^{1}H) = 107 ms.

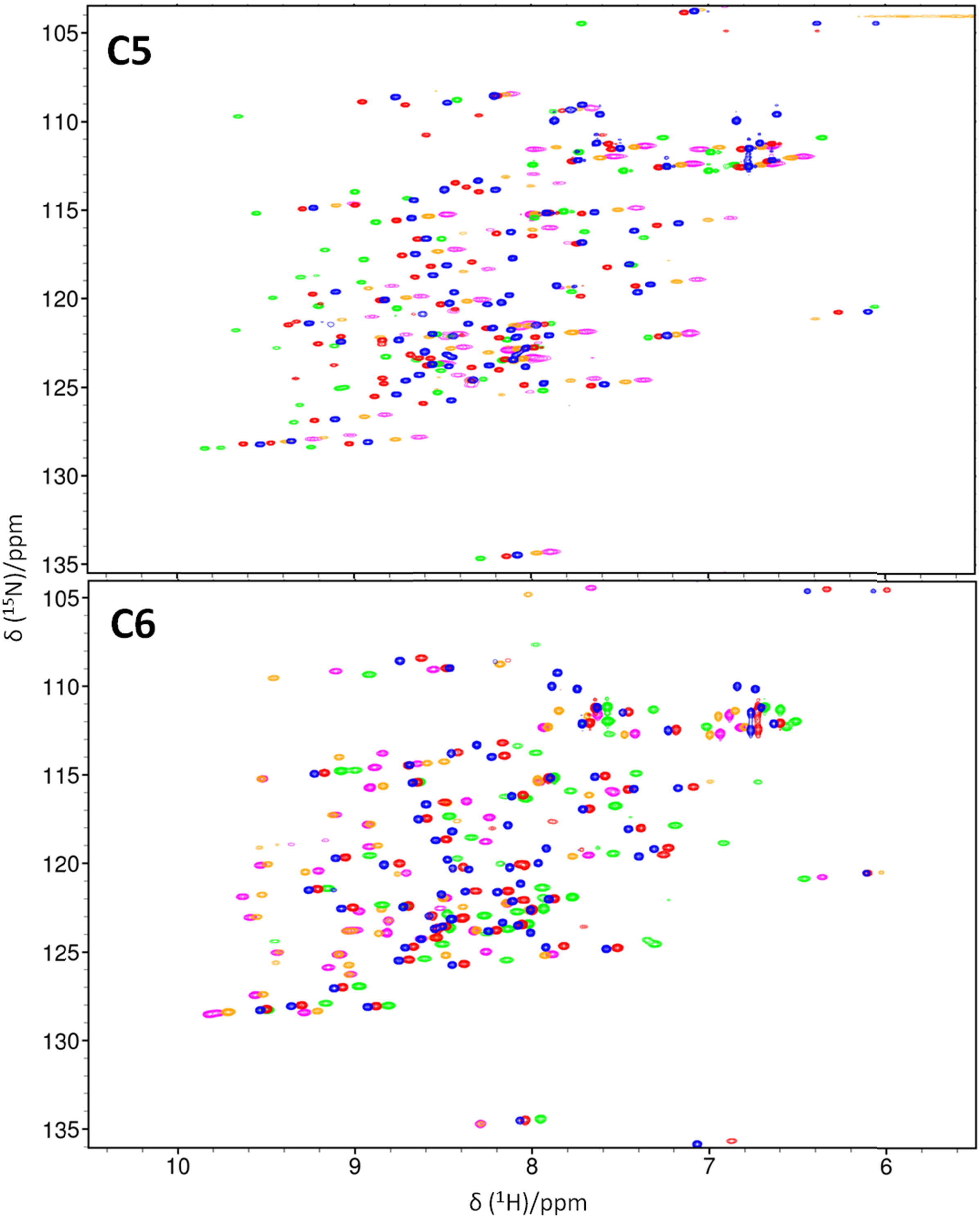

Figure S7. Overlay of ^{15}N-HSQC spectra of **C5** (top spectra) and **C6** (bottom spectra) tagged ubiquitin A28C, loaded with Y^{3+} (blue), Dy^{3+} (magenta), Tb^{3+} (orange), Tm^{3+} (green) or Yb^{3+} (red). The spectra were recorded at 25 °C and pH 8 at a ^{1}H NMR frequency of 600 MHz.

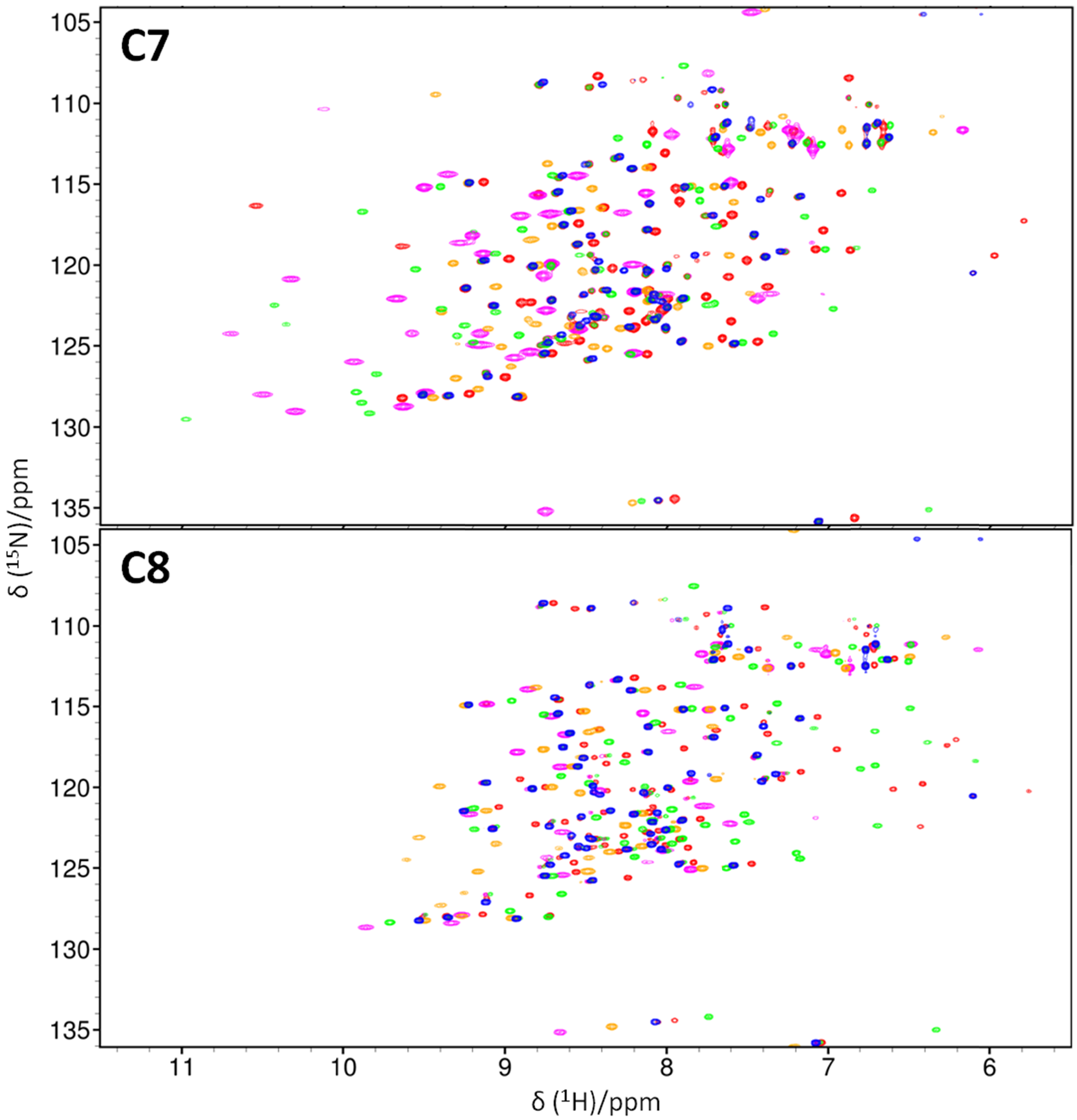

Figure S8. Overlay of ^{15}N-HSQC spectra of **C7** (top spectra) and **C8** (bottom spectra) tagged ubiquitin A28C, loaded with Y^{3+} (blue), Dy^{3+} (magenta), Tb^{3+} (orange), Tm^{3+} (green) or Yb^{3+} (red). The spectra were recorded at 25 °C and pH 8 at a ^{1}H NMR frequency of 600 MHz.

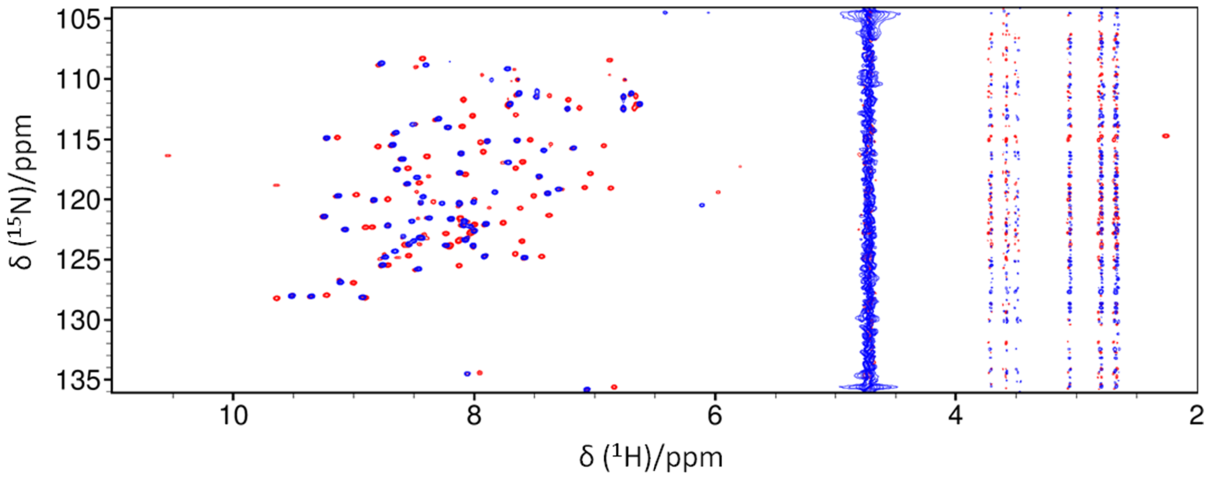

Figure S9. Overlay of ^{15}N-HSQC spectra of **C7** tagged ubiquitin A28C, loaded with Y^{3+} (blue) and Yb^{3+} (red). The spectra are the same as in the top panel of Figure S7, except that a larger spectral width is displayed, and only Y^{3+} and Yb^{3+} data are included.

Table S2. Experimental PCSs for **C5** and **C6** tagged ubiquitin A28C.

Residue		C5 Dy^{3+}	C5 Tb^{3+}	C5 Tm^{3+}	C5 Yb^{3+}	C6 Dy^{3+}	C6 Tb^{3+}	C6 Tm^{3+}	C6 Yb^{3+}
2	GLN	-0.212	-0.125	0.315	0.030	0.293	0.307	-0.253	-0.059
3	ILE	-0.349	-0.198	0.500	0.094	0.422	0.368	-0.248	-0.071
4	PHE	-0.305	-0.168	0.405	0.102	0.377	0.329	-0.199	-0.059
5	VAL	-0.354	-0.184	0.414	0.119	0.375	0.265	-0.105	-0.050
6	LYS	-0.287	-0.154	0.320	0.106	0.357	0.283	-0.114	-0.046
7	THR	-0.196	-0.095	0.202	0.086	0.241	0.168	-0.036	-0.027
8	LEU	-0.131	-0.061	0.116	0.059	0.069	0.176		
9	THR								
10	GLY	-0.115	-0.049	0.097	0.047				
11	LYS	-0.127	-0.053	0.104	0.044	0.159	0.100	-0.010	-0.021
12	THR	-0.170	-0.080	0.153	0.051				
13	ILE	-0.295	-0.145	0.312	0.096	0.284	0.171	-0.039	-0.036
14	THR	-0.266	-0.172	0.361	0.091	0.248	0.123	-0.014	-0.031
15	LEU	-0.419	-0.224	0.548	0.126	0.396	0.281	-0.145	-0.057
16	GLU		-0.311	0.760	0.095	0.409	0.279	-0.178	-0.063
17	VAL	-0.361	-0.206	0.572	0.049	0.513	0.528	-0.447	-0.105
18	GLU	-0.376	-0.178	0.825	0.090	0.718	1.088	-1.004	-0.227
20	SER	-0.170	-0.039	0.636	0.053	0.599	0.954	-1.009	-0.194
21	ASP	-0.378	-0.155	1.044	0.149	1.055	1.438	-1.398	-0.298
22	THR				0.580				
23	ILE				0.515				
24	GLU								
25	ASN								
26	VAL				0.731				
27	LYS				0.756				
28	CYS				1.454				
29	LYS				1.029				
30	ILE				0.712				-0.059
31	GLN				0.863			0.947	-0.020
32	ASP				1.071			1.293	
33	LYS				0.563	0.133	-0.432	0.610	0.031
34	GLU				0.331	0.198	-0.196	0.386	0.004
35	GLY			0.118	0.237	0.092	-0.283	0.456	0.017
36	ILE		0.295	-0.042	0.162	0.248	-0.085	0.350	-0.005
39	ASP				-0.125	0.654		-0.372	-0.037
40	GLN		0.026	-0.350	0.028	0.529	0.709	-0.182	-0.036
41	GLN		-0.224	-0.022	0.121	0.806	0.876	-0.267	-0.071
42	ARG		-0.234	0.173	0.091	0.572	0.585	-0.188	-0.052
43	LEU		-0.354	0.460	0.212	0.813	0.800	-0.372	-0.088

44	ILE	-0.366	-0.219	0.366	0.129	0.514	0.479	-0.228	-0.064
45	PHE	-0.294	-0.186	0.345	0.124	0.371	0.373	-0.209	-0.048
46	ALA								
47	GLY	-0.188	-0.114	0.206	0.059	0.221	0.219	-0.117	-0.029
48	LYS	-0.207	-0.142	0.240	0.078	0.238	0.242	-0.130	-0.029
49	GLN	-0.215	-0.145	0.220	0.081	0.230	0.228	-0.103	-0.018
50	LEU	-0.448	-0.282	0.428	0.158	0.575	0.585	-0.307	-0.066
51	GLU		-0.373	0.575	0.190	0.552	0.571	-0.356	-0.063
52	ASP				0.257			-0.588	-0.082
53	GLY								
54	ARG		-0.523		0.320	0.760		-0.889	-0.142
55	THR	-0.404	-0.226	0.889	0.189	0.360	0.711	-0.767	-0.122
56	LEU	-0.549	-0.268	1.106	0.228	1.224	1.302	-1.172	-0.250
57	SER	-0.316	-0.156	0.694	0.127	0.533	0.776	-0.745	-0.146
58	ASP	-0.288	-0.152	0.597	0.110	0.338	0.565	-0.574	-0.101
59	TYR	-0.294	-0.168	0.530	0.118	0.369	0.504	-0.449	-0.085
60	ASN	-0.226	-0.123	0.390	0.080	0.255	0.364	-0.332	-0.063
61	ILE	-0.266	-0.150	0.443	0.085	0.371	0.462	-0.391	-0.081
62	GLN	-0.223	-0.123	0.339	0.069	0.303	0.346	-0.276	-0.062
63	LYS	-0.174	-0.099	0.296	0.049	0.269	0.320	-0.263	-0.052
64	GLU	-0.221	-0.125	0.323	0.062	0.295	0.300	-0.228	-0.056
65	SER	-0.236	-0.133	0.335	0.071	0.312	0.319	-0.232	-0.057
66	THR	-0.228	-0.131	0.289	0.074	0.287	0.273	-0.171	-0.046
67	LEU	-0.329	-0.185	0.399	0.117	0.417	0.374	-0.194	-0.060
68	HIS	-0.313	-0.182	0.358	0.130	0.424	0.385	-0.191	-0.054
69	LEU	-0.287	-0.142	0.270	0.097	0.380	0.310	-0.108	-0.046
70	VAL	-0.278	-0.164	0.234	0.115	0.447	0.404	-0.139	-0.047

Table S3. Experimental PCSs for **C7** and **C8** tagged ubiquitin A28C.

		**C7**				**C8**			
Residue		Dy^{3+}	Tb^{3+}	Tm^{3+}	Yb^{3+}	Dy^{3+}	Tb^{3+}	Tm^{3+}	Yb^{3+}
2	GLN	0.007	-0.068	0.151	-0.077	-0.428	-0.053	-0.233	-0.093
3	ILE	0.338	-0.094	0.492	-0.119	-0.387	-0.087	-0.306	-0.189
4	PHE	0.585	0.000	0.508	-0.099	0.107	0.034	-0.290	-0.177
5	VAL	0.429	-0.187	1.184	0.004	-0.033	-0.140	-0.061	-0.216
6	LYS	0.705	-0.022	0.914	-0.020	0.406	0.028	-0.197	-0.206
7	THR	0.124	-0.211	1.210	0.121	0.041	-0.161	0.090	-0.136
8	LEU								
9	THR								
10	GLY								
11	LYS					-0.151	-0.212	0.196	-0.076
12	THR								
13	ILE	-0.014	-0.345	1.464	0.125	-0.260	-0.265	0.180	-0.171
14	THR	-0.710	-0.589	1.642	0.187	-0.923	-0.468	0.463	-0.095
15	LEU	0.094	-0.304	1.042	-0.043	-0.627	-0.284	-0.048	-0.194
16	GLU	-0.646	-0.600	0.974	-0.088				
17	VAL	0.133	-0.045	0.012	-0.203		-0.118	-0.530	-0.184
18	GLU	0.808	0.353	-0.775	-0.400		0.005	-1.196	-0.266
20	SER	0.416	0.330	-0.684	-0.226	-0.528	0.136	-0.746	-0.030
21	ASP	1.159	0.564	-1.036	-0.408	-0.013	0.447	-1.343	-0.166
22	THR								
23	ILE			-1.960	-0.900			-2.441	
24	GLU								
25	ASN				-2.435				
26	VAL				-2.109				-1.464
27	LYS				-2.630				
28	CYS				-6.010				
29	LYS								
30	ILE								-1.932
31	GLN							-0.464	
32	ASP								
33	LYS				2.219				
34	GLU				1.893				
35	GLY								
36	ILE				1.941				-0.352
39	ASP								
40	GLN		0.368	1.479	-0.122			-1.003	-0.336
41	GLN			1.082	-0.425			-1.054	-0.491
42	ARG		0.355	0.798	-0.223			-0.716	-0.353
43	LEU		0.643	-0.002	-0.434			-1.056	-0.333

44	ILE	1.622	0.327	0.325	-0.228		0.454	-0.642	-0.265
45	PHE	1.200	0.291	0.010	-0.194	1.013	0.449	-0.522	-0.146
46	ALA								
47	GLY	0.697	0.157	0.101	-0.103	0.588	0.266	-0.330	-0.120
48	LYS	0.845	0.216	-0.015	-0.142	0.741	0.340	-0.390	-0.112
49	GLN	0.992	0.273	-0.132	-0.172		0.470	-0.439	-0.106
50	LEU	1.914	0.505	-0.235	-0.336		0.797	-0.831	-0.218
51	GLU		0.683	-0.671	-0.396		1.158	-0.961	-0.187
52	ASP			-1.308	-0.627			-1.440	-0.174
53	GLY								
54	ARG			-1.317	-0.524				-0.121
55	THR	1.351	0.667	-0.867	-0.339		0.963	-0.929	-0.061
56	LEU		0.730	-0.972	-0.466		0.931		-0.220
57	SER	1.065	0.447	-0.607	-0.280	0.561	0.509	-0.836	-0.102
58	ASP	1.027	0.453	-0.574	-0.258	0.717	0.567	-0.730	-0.093
59	TYR	1.101	0.413	-0.445	-0.251	0.817	0.546	-0.684	-0.109
60	ASN	0.798	0.298	-0.310	-0.185	0.517	0.311	-0.513	-0.087
61	ILE	0.913	0.314	-0.276	-0.217	0.533	0.372	-0.528	-0.155
62	GLN	0.627	0.167	-0.049	-0.142	0.270	0.198	-0.410	-0.111
63	LYS	0.324	0.084	-0.027	-0.104	-0.037	0.085	-0.316	-0.083
64	GLU	0.282	0.005	0.181	-0.088	-0.112	0.021	-0.268	-0.109
65	SER	0.490	0.068	0.160	-0.107	0.105	0.093	-0.323	-0.121
66	THR	0.567	0.075	0.257	-0.092	0.286	0.122	-0.286	-0.130
67	LEU	0.949	0.100	0.539	-0.127	0.509	0.144	-0.385	-0.216
68	HIS	1.198	0.197	0.429	-0.150	0.900	0.291	-0.463	-0.209
69	LEU	0.920	0.051	0.966	-0.028	0.713	0.105	-0.279	-0.242
70	VAL	1.387	0.199	0.812	-0.109		0.277	-0.469	-0.270

**Table S4.** $\Delta\chi$-Tensor parameters for **C5–C8** tagged ubiquitin A28C (common fits).								
Tag	Ln^{3+}	# PCS	$\Delta\chi_{ax}$	$\Delta\chi_{rh}$	Q	α	β	γ
**C5**	Dy^{3+}	39	12.2 (0.6)	5.7 (0.2)	0.06	146	98	98
	Tb^{3+}	47	11.3 (0.5)	2.6 (0.2)	0.09	154	97	129
	Tm^{3+}	47	-15.4	-9.5	0.08	122	88	101
	Yb^{3+}	61	-6.6 (0.3)	-1.4 (0.2)	0.09	119	100	111
**C6**	Dy^{3+}	49	9.7	5.7	0.09	34	147	82
	Tb^{3+}	47	-14.1 (0.3)	-3.1 (0.1)	0.05	44	64	100
	Tm^{3+}	51	11.8 (0.3)	4.0 (0.1)	0.10	40	71	132
	Yb^{3+}	51	1.9 (0.1)	1.0 (0.0)	0.12	47	62	107
**C7**	Dy^{3+}	35	34.6 (0.6)	4.7 (0.7)	0.07	59	35	24
	Tb^{3+}	40	12.7 (0.3)	0.8 (0.3)	0.04	42	45	71
	Tm^{3+}	44	-22.0	-12.2	0.07	32	61	63
	Yb^{3+}	51	-8.1 (0.3)	-2.1 (0.5)	0.05	41	39	23
**C8**	Dy^{3+}	28	37.1 (1.1)	8.5 (0.7)	0.03	88	46	2
	Tb^{3+}	37	18.6 (0.7)	7.8 (0.6)	0.07	71	48	8
	Tm^{3+}	43	-15.4	-10.0	0.05	75	26	40
	Yb^{3+}	46	-4.6 (0.1)	-1.9 (0.2)	0.05	129	23	176

The axial and rhombic components of the $\Delta\chi$-tensors are reported in units of 10^{-32} m^3, and the Euler angles in degrees, using the *zyz* convention and unique tensor representation.[4] The determined metal coordinates (x, y, z) for each tag relative to the first structure of the NMR structure of ubiquitin (PDB ID 2MJB)[5] are: C5 2.507, -2.130, -17.878; C6 7.278, 3.052, -12.752; C7 –2.505, -3.481, -14.778; C8 1.396, -3.879, -14.698. Standard deviations (in brackets) were determined from random removal of 10% of the PCSs and recalculating the $\Delta\chi$-tensor 1,000 times, in some cases the *z* and *y* axes of the tensor were of similar magnitude and swapped in different fits, thus standard deviations were not determined. Quality factors (Q) were calculated as the root-mean-square deviation between the experimental and back-calculated PCSs divided by the root-mean-square of the experimental PCSs.

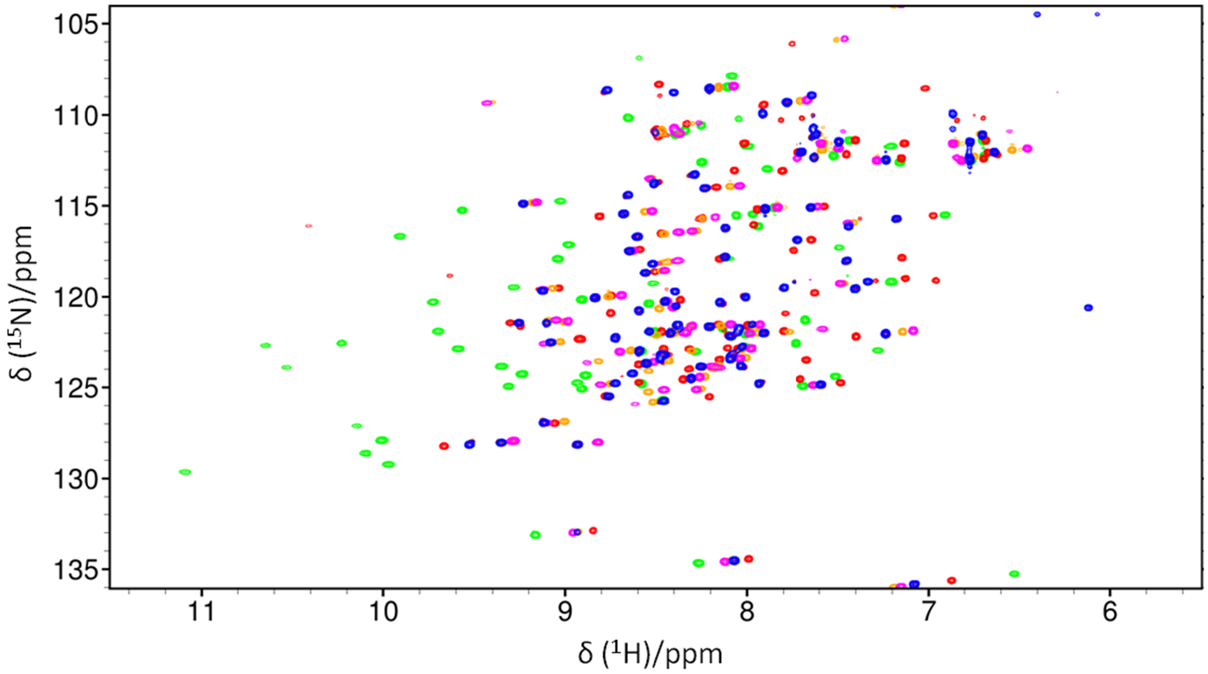

Figure S10. Overlay of ^{15}N-HSQC spectra of **C7** tagged ubiquitin A28C, loaded with Y^{3+} (blue), Dy^{3+} (magenta), Tb^{3+} (orange), Tm^{3+} (green) or Yb^{3+} (red). The spectra were recorded at 25 °C and pH 6.5 at a ^{1}H NMR frequency of 600 MHz.

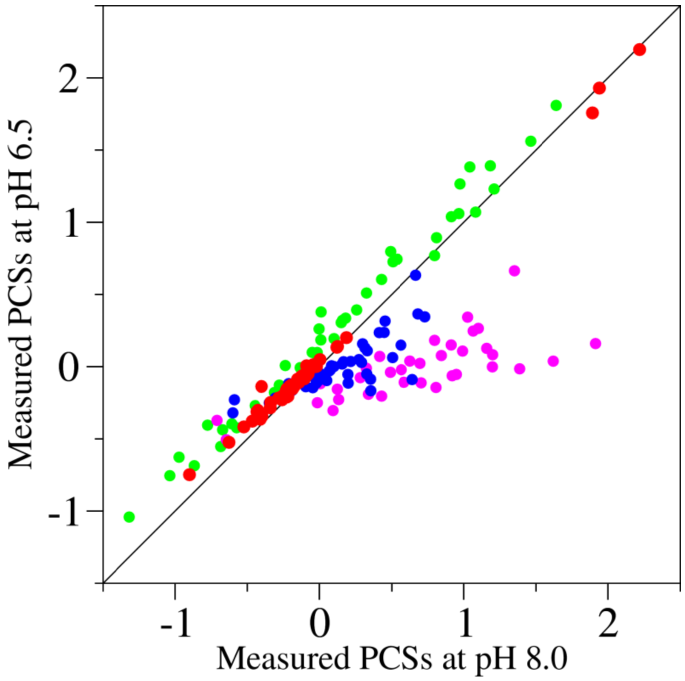

Figure S11. Correlations between measured PCSs at pH 8.0 and pH 6.5 for **C7** tagged ubiquitin A28C, loaded with Dy^{3+} (magenta), Tb^{3+} (blue), Tm^{3+} (green) or Yb^{3+} (red). Only PCSs which were assigned at both pH values are shown. The solid line represents a perfect correlation.

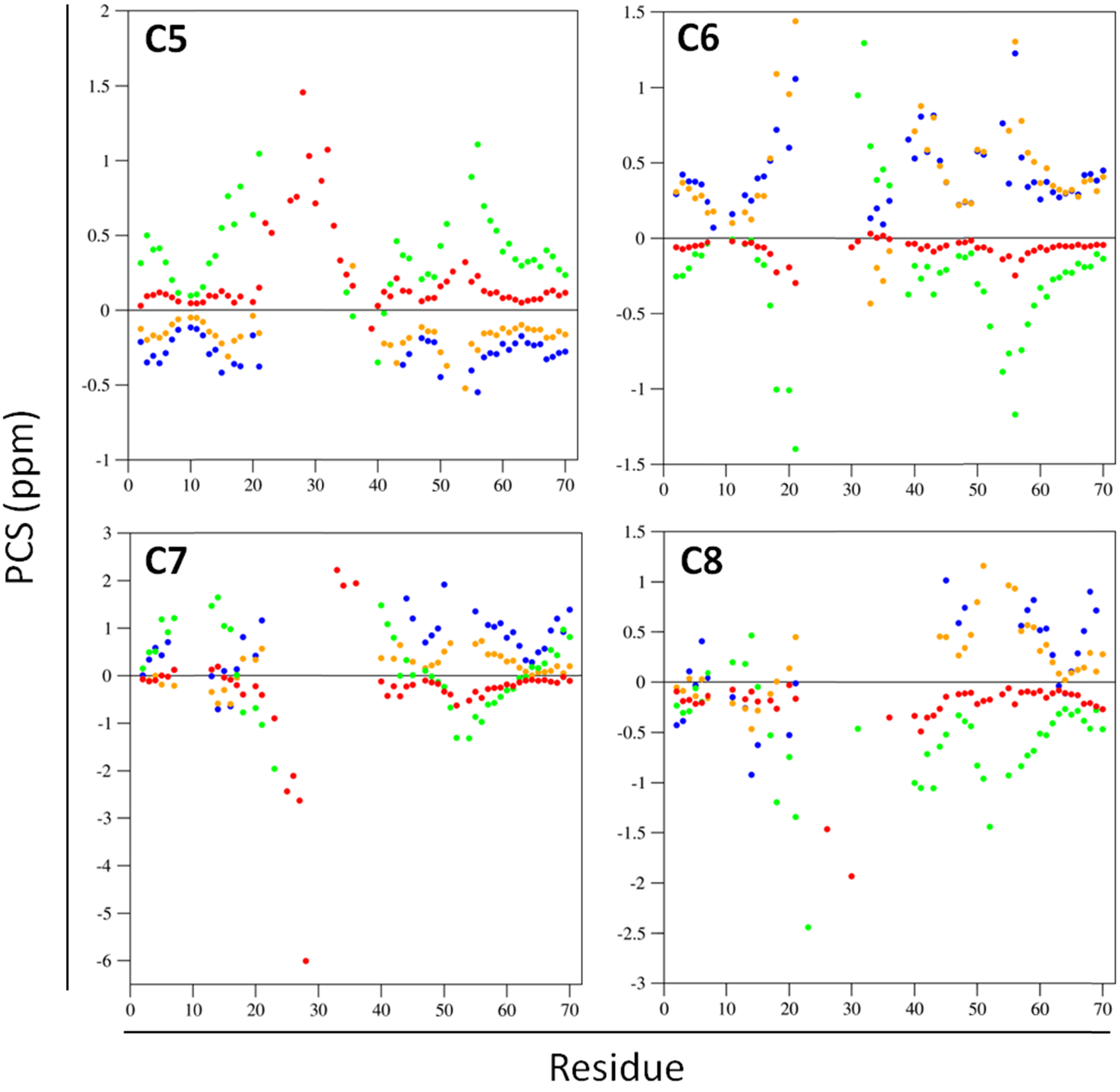

Figure S12. Plots of PCSs measured at 25 °C and pH 8 *vs.* residue number for **C5–C8** tagged ubiquitin A28C. Colour scheme: Dy^{3+} - blue, Tb^{3+} - orange, Tm^{3+} - green, Yb^{3+} - red.

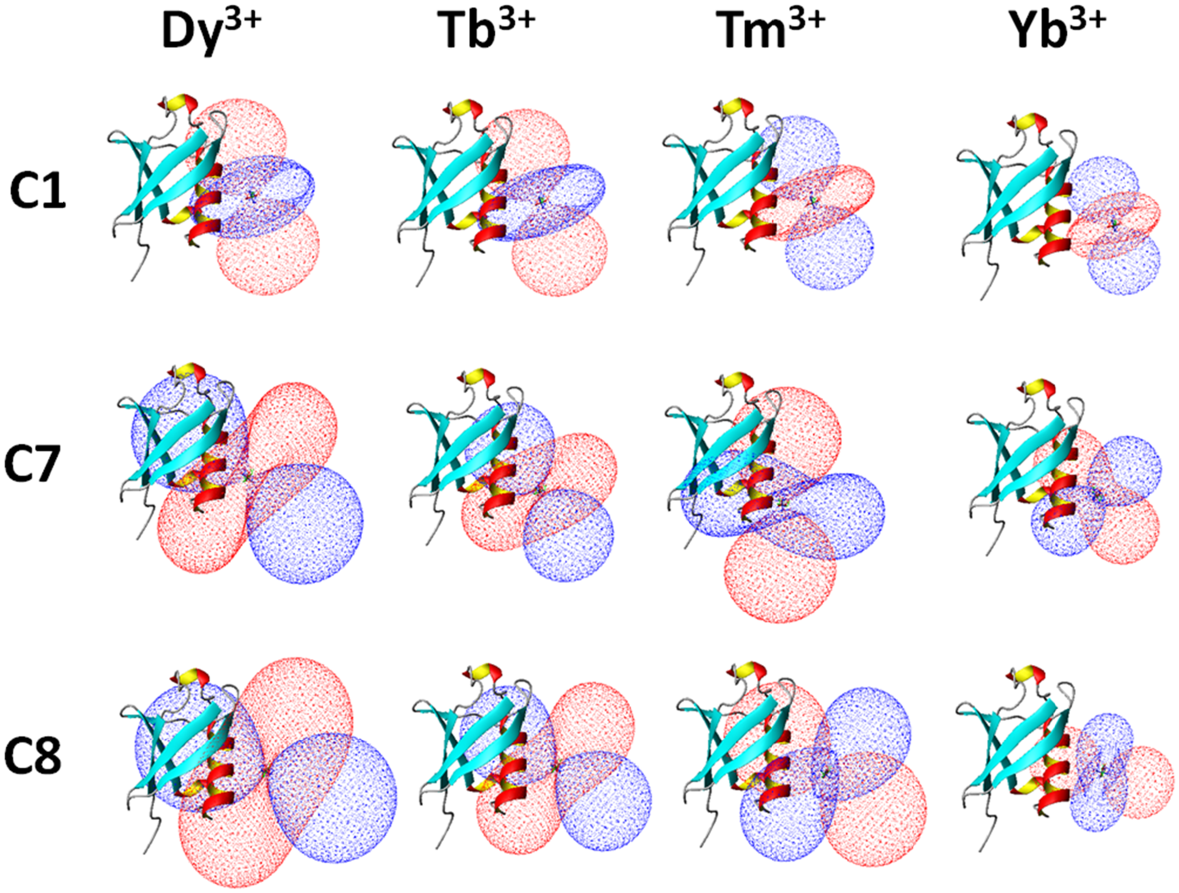

Figure S13. Comparison of $\Delta\chi$-tensor orientations and individual metal positions determined for different metal complexes of **C1**, **C7** and **C8** bound to ubiquitin A28C. Blue/red isosurfaces indicate positive/negative PCSs of 1 ppm.

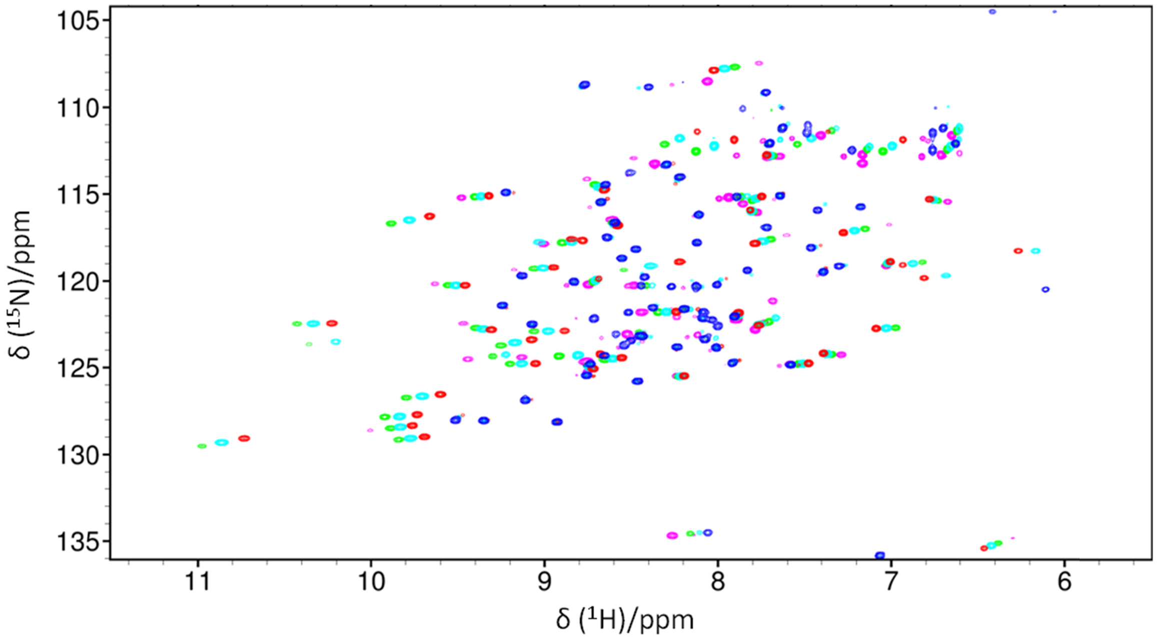

Figure S14. Overlay of ^{15}N-HSQC spectra of **C7** tagged ubiquitin A28C, loaded with Y^{3+} at 25 °C (blue) or Tm^{3+} at 10 °C (magenta), 25 °C (green), 32 °C (cyan) and 40 °C (red). The spectra were recorded at pH 8 at a ^{1}H NMR frequency of 600 MHz.

Table S5. $^{1}D_{HN}$ RDCs of **C7** and **C8** tagged ubiquitin A28C loaded with Tm^{3+}, measured at 600 MHz.

**C7-Tm^{3+}**			**C8-Tm^{3+}**		
Residue		$^{1}D_{HN}$ RDC (Hz)	Residue		$^{1}D_{HN}$ RDC (Hz)
2	GLN	1.82	2	GLN	-2.86
3	ILE	-2.67	3	ILE	-4.99
4	PHE	-7.35	4	PHE	-3.77
5	VAL	-8.08	5	VAL	0.06
6	LYS	-7.41	6	LYS	1.03
7	THR	-2.25	7	THR	3.22
13	ILE	-7.73	13	ILE	0.24
15	LEU	-2.73	14	THR	-1.83
17	VAL	3.60	15	LEU	-5.35
18	GLU	-0.17	17	VAL	-1.87
20	SER	-5.36	18	GLU	-0.30
44	ILE	-3.65	20	SER	0.19
47	GLY	-6.98	40	GLN	-6.01
50	LEU	-6.08	41	GLN	2.55
55	THR	-8.09	43	LEU	6.14
57	SER	4.80	44	ILE	3.95
58	ASP	-1.21	45	PHE	3.33
59	TYR	-5.83	47	GLY	1.65
61	ILE	12.46	48	LYS	0.91
62	GLN	5.65	49	GLN	2.43
64	GLU	4.38	50	LEU	4.43
65	SER	9.30	55	THR	-0.68
66	THR	-2.80	57	SER	-3.28
67	LEU	-7.40	58	ASP	-5.40
68	HIS	-4.86	59	TYR	-3.45
69	LEU	3.28	61	ILE	1.22
70	VAL	6.27	62	GLN	0.55
			64	GLU	-4.44
			65	SER	2.67
			66	THR	-4.87
			67	LEU	-1.52
			68	HIS	3.28
			69	LEU	4.68
			70	VAL	5.41

**Table S6.** Alignment tensor parameters for **C7** and **C8** tagged ubiquitin A28C loaded with Tm^{3+}.[a]										
Tag	Ln^{3+}	# RDC	A_{ax}	A_{rh}	*Q*	α	β	γ	$\Delta\chi_{ax}$[b]	$\Delta\chi_{rh}$[b]
**C7**	Tm^{3+}	27	5.33	1.67	0.13	105	140	164	20.1	6.5
**C8**	Tm^{3+}	34	2.77	1.41	0.23	90	104	154	10.1	5.5

[a] The axial and rhombic components of the alignment tensor are reported in units of 10^{-4} and the Euler angles in degrees, using the *zyz* convention. Quality factors (*Q*) were calculated as the root-mean-square deviation between the experimental and back-calculated RDCs divided by the root-mean-square of the experimental RDCs.
[b] Δχ-Tensor parameters in units of 10^{-32} m^3 determined from the A_{ax} and A_{rh} using Equation S1.

Equation S1. For comparison of $A_{ax,rh}$ and $\Delta\gamma_{ax,rh}$

$$\Delta\chi_{ax,rh} = A_{ax,rh}\frac{15\mu_0 KT}{B_0^2}$$

where B_0 is the field strength (14.1 T), μ_0 is the magnetic permeability of vacuum (12.566 x 10^{-7} T^2 m^3 J^{-1}), *k* is the Boltzmann constant (1.38 x 10^{-23} J K^{-1}), *T* is temperature (in Kelvin), $\Delta\chi_{ax,rh}$ are the axial and rhombic components of the magnetic susceptibility anisotropy tensor (in m^3) respectively and $A_{ax,rh}$ are the axial and rhombic components of the alignment tensor respectively.

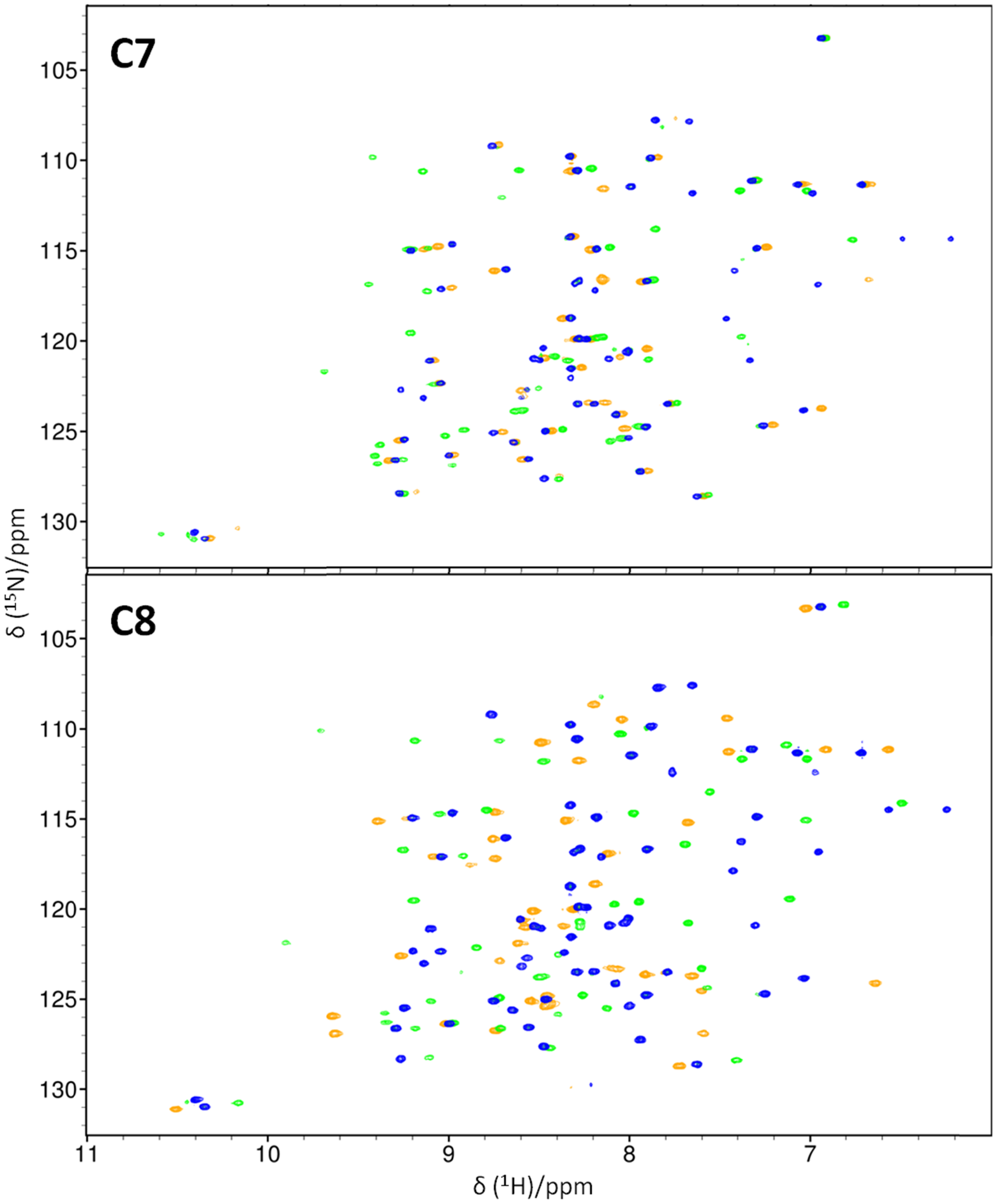

Figure S15. Overlay of ^{15}N-HSQC spectra of **C7** (top spectra) and **C8** (bottom spectra) tagged GB1 Q32C, loaded with Y^{3+} (blue), Tb^{3+} (orange) or Tm^{3+} (green). The spectra were recorded at 25 °C and pH 6.5 at a ^{1}H NMR frequency of 600 MHz.

**Table S7.** Δχ-Tensor parameters for C7 and C8 tagged GB1 Q32C (common fits).[a,b]								
Tag	Ln^{3+}	# PCS	$\Delta\chi_{ax}$	$\Delta\chi_{rh}$	*Q*	α	β	γ
**C7**	Tb^{3+}	47	2.9 (0.2)	1.5 (0.1)	0.13	28	35	47
	Tm^{3+}	37	-13.8 (0.7)	-4.7 (0.3)	0.06	145	55	79
**C8**	Tb^{3+}	40	-5.7	-3.5	0.05	73	90	57
	Tm^{3+}	40	-15.0 (0.4)	-4.1 (0.5)	0.06	164	54	35

[a] See footnote a in Table 1.
[b] The determined metal coordinates (x, y, z) for each tag relative to the crystal structure of GB1 (PDB ID 1PGA[6]) are: C7 31.338, 29.630, 12.581; C8 32.563, 31.104, 14.230.

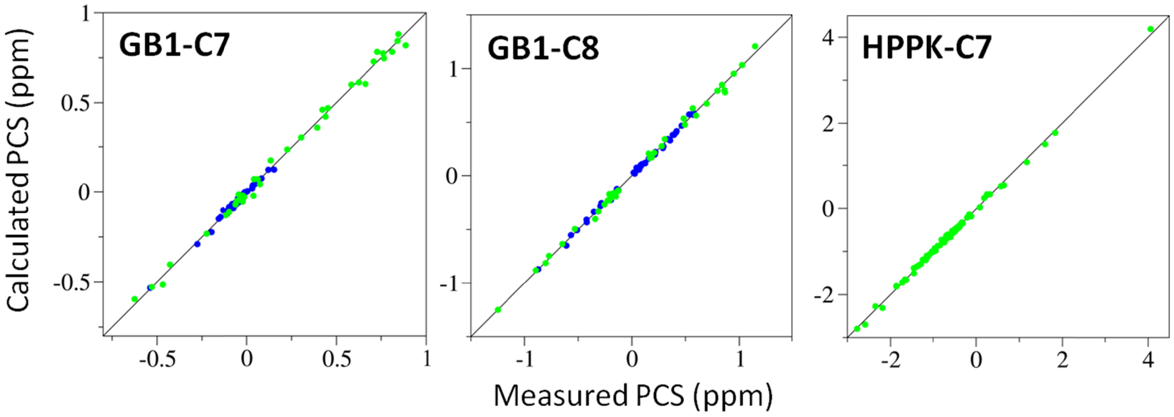

Figure S16. Correlations between experimental and back-calculated PCSs for **C7** and **C8** tagged GB1 Q32C and **C7** tagged HPPK S112C/C80A, loaded with Tb^{3+} (blue) or Tm^{3+} (green). Solid lines represent perfect correlation.

Table S8. Experimental PCSs for **C7** and **C8** tagged GB1 Q32C.

Residue		C7		C8	
		Tb^{3+}	Tm^{3+}	Tb^{3+}	Tm^{3+}
2	THR	0.031	-0.032	0.217	-0.210
3	TYR	0.039	-0.044	0.336	-0.312
4	LYS	0.004	0.041	0.221	-0.200
5	LEU	0.035	0.422	0.182	0.157
6	ILE	-0.018	0.393	0.016	0.187
7	LEU	-0.007	0.766	-0.181	0.697
8	ASN	-0.048	0.625	-0.293	0.598
9	GLY	-0.039	0.728	-0.420	0.839
10	LYS	-0.022	0.583	-0.511	0.797
11	THR	-0.034	0.662	-0.567	0.948
12	LEU	-0.050	0.846	-0.610	1.146
13	LYS	-0.029	0.841	-0.422	1.025
14	GLY	-0.010	0.811	-0.284	0.862
15	GLU	0.044	0.886	-0.140	0.867
16	THR	0.065	0.761	0.068	0.568
17	THR	0.153	0.709	0.291	0.484
18	THR	0.083	0.134	0.412	-0.194
19	GLU	0.121	0.038	0.535	-0.342
20	ALA	0.033	-0.224	0.394	-0.531
21	VAL	-0.012	-0.468	0.418	-0.771
22	ASP	-0.049	-0.529	0.382	-0.803
23	ALA	-0.058	-0.428	0.296	-0.647
24	ALA	-0.100	-0.625	0.358	-0.894
25	THR	-0.131		0.465	-1.249
26	ALA	-0.097		0.571	
27	GLU	-0.157		0.575	
28	LYS	-0.276			
29	VAL	-0.197			
30	PHE	-0.146			
31	LYS	-0.538			
32	CYS				
33	TYR				
34	ALA				
35	ASN				
36	ASP				
37	ASN				
38	GLY				
39	VAL				
40	ASP	-0.080		-0.872	

41	GLY	-0.109				0.309
42	GLU	-0.061	0.226			0.167
43	TRP	-0.095	-0.022			-0.162
44	THR	-0.073			0.027	-0.149
45	TYR	-0.056	-0.116		0.043	-0.256
46	ASP	-0.035	-0.062		0.095	-0.226
47	ASP	-0.035	-0.097		0.082	-0.208
48	ALA	-0.024	-0.060		0.075	-0.154
49	THR	-0.015	-0.013		0.083	-0.128
50	LYS	-0.016	-0.053		0.123	-0.188
51	THR	-0.025	-0.022		0.124	-0.193
52	PHE	-0.031	0.062		0.157	-0.192
53	THR	-0.063	0.075		0.040	-0.126
54	VAL	-0.060	0.439		-0.125	0.280
55	THR	-0.067	0.304		-0.196	0.211
56	GLU	-0.041	0.452		-0.354	0.495

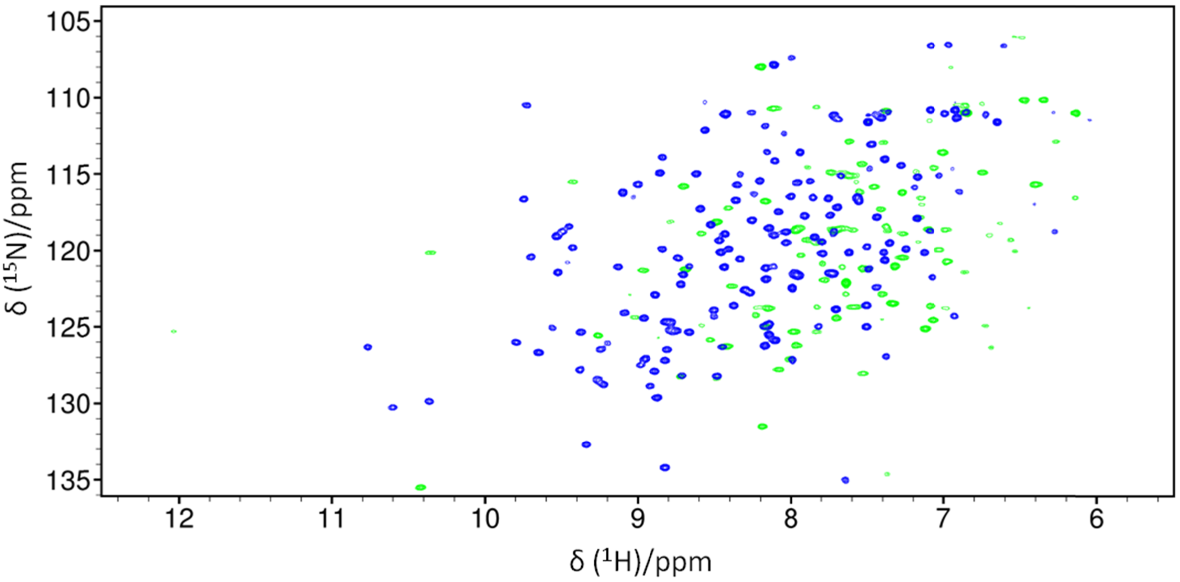

Figure S17. Overlay of ^{15}N-HSQC spectra of **C7** tagged HPPK S112C/C80A, loaded with Y^{3+} (blue) and Tm^{3+} (green). The spectra were recorded at 22 °C and pH 8 at a ^{1}H NMR frequency of 600 MHz, in the presence of 10 mM $MgCl_2$, 1 mM α,β-methyleneadenosine 5'-triphosphate and 400 μM of a small molecule inhibitor.

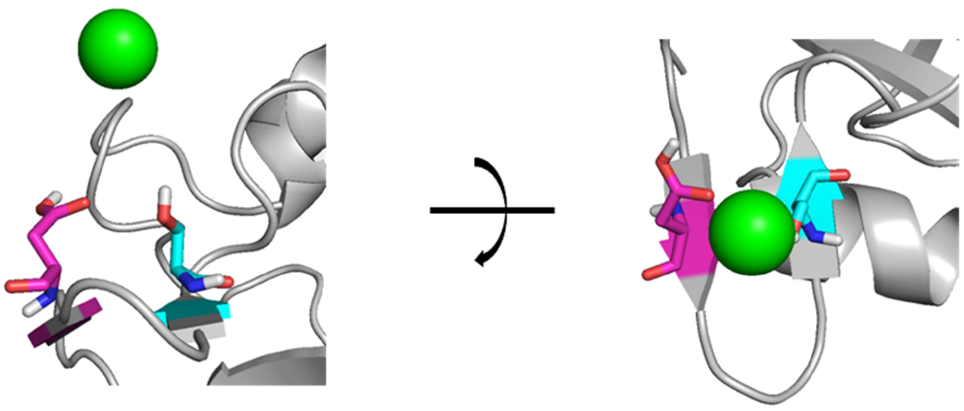

Figure S18. Structure of HPPK with the PCS-determined metal ion position for **C7** tagged HPPK S112C/C80A loaded with Tm^{3+}. The Tm^{3+} ion is represented by a green sphere. D107 and S112 are shown as sticks in magenta and cyan, respectively.

Table S9. Experimental PCS for **C7** tagged HPPK S112C/C80A loaded with Tm^{3+}.

C7-Tm^{3+}								
Residue		PCS	Residue		PCS	Residue		PCS
2	ILE	-0.990	42	GLU	-0.533	102	GLY	-1.865
3	GLN	-1.049	43	THR	-0.703	103	GLU	-1.252
4	ALA	-1.348	44	ALA	-0.689	122	ALA	-1.15
5	TYR	-2.350	50	GLU	-0.485	128	LEU	-0.934
6	LEU	-1.864	51	GLN	-0.479	130	ASP	-0.423
7	GLY	-2.774	53	ASN	-0.466	131	ILE	-0.479
8	LEU	-1.665	54	PHE	-0.732	132	ALA	-0.402
9	GLY	-1.461	55	LEU	-0.763	133	ALA	-0.112
10	SER	-0.952	56	ASN	-0.838	134	ASN	0.089
11	ASN	-0.676	57	LEU	-1.220	135	VAL	0.085
12	ILE	-0.497	58	CYS	-1.043	136	VAL	0.319
13	GLY	-0.310	60	GLU	-0.975	137	GLU	1.596
14	ASP	-0.321	63	THR	-1.047	142	LEU	4.056
15	ARG	-0.412	64	THR	-0.892	143	LYS	1.830
16	GLU	-0.424	65	LEU	-1.101	144	VAL	0.643
17	SER	-0.380	66	THR	-1.184	145	LYS	0.575
19	LEU	-0.591	67	VAL	-2.186	146	ASP	1.174
20	ASN	-0.542	69	GLN	-1.458	149	PHE	0.187
21	ASP	-0.501	70	LEU	-1.729	150	VAL	0.252
22	ALA	-0.589	72	GLU	-1.297	151	ASP	-0.160
23	ILE	-0.799	73	CYS	-1.135	152	ASP	-0.205
24	LYS	-0.654	74	CYS	-1.404	153	SER	-0.398
29	TYR	-0.689	75	LEU	-1.162	154	VAL	-0.436
30	ASP	-0.555	81	LEU	-0.462	155	LYS	-0.496
31	GLY	-0.615	95	ASP	-1.187	156	ARG	-0.330
36	ASN	-0.741	96	VAL	-1.639	157	TYR	-0.433
41	TYR	-0.716	97	ASP	-2.588	158	LYS	-0.370

Figure S19. ^{1}H and ^{13}C NMR spectra of **C5**.

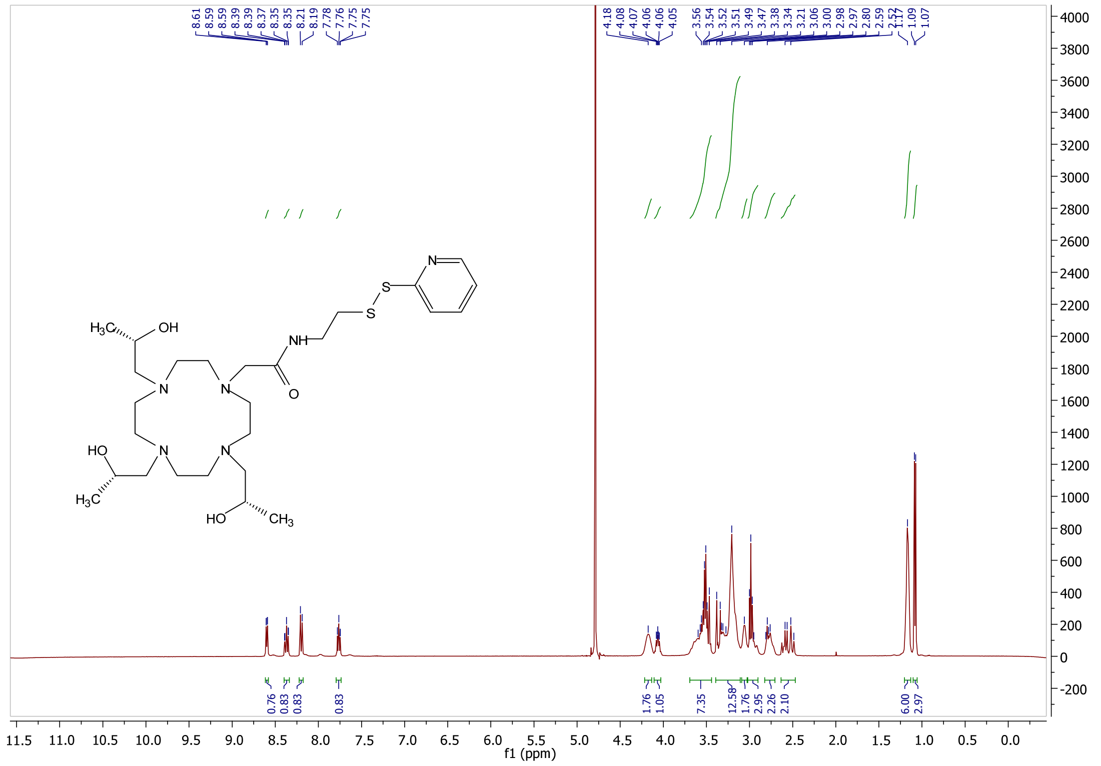

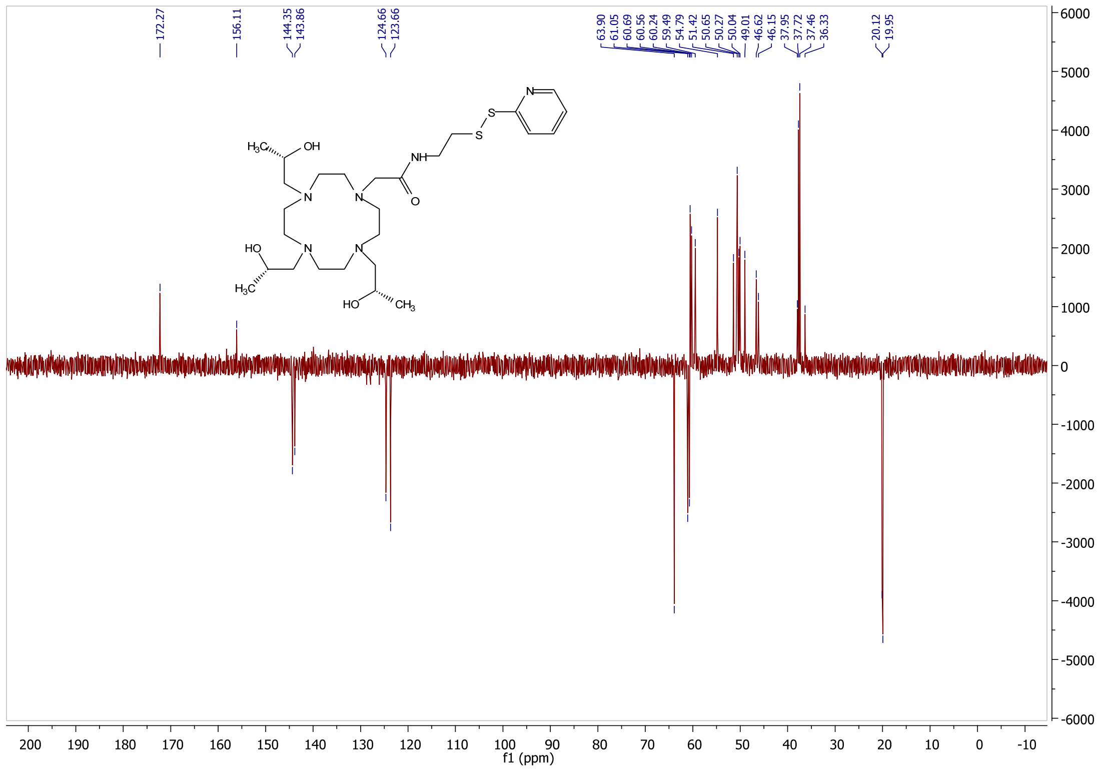

Figure S20. ^{1}H NMR spectrum of **C5-Y^{3+}**.

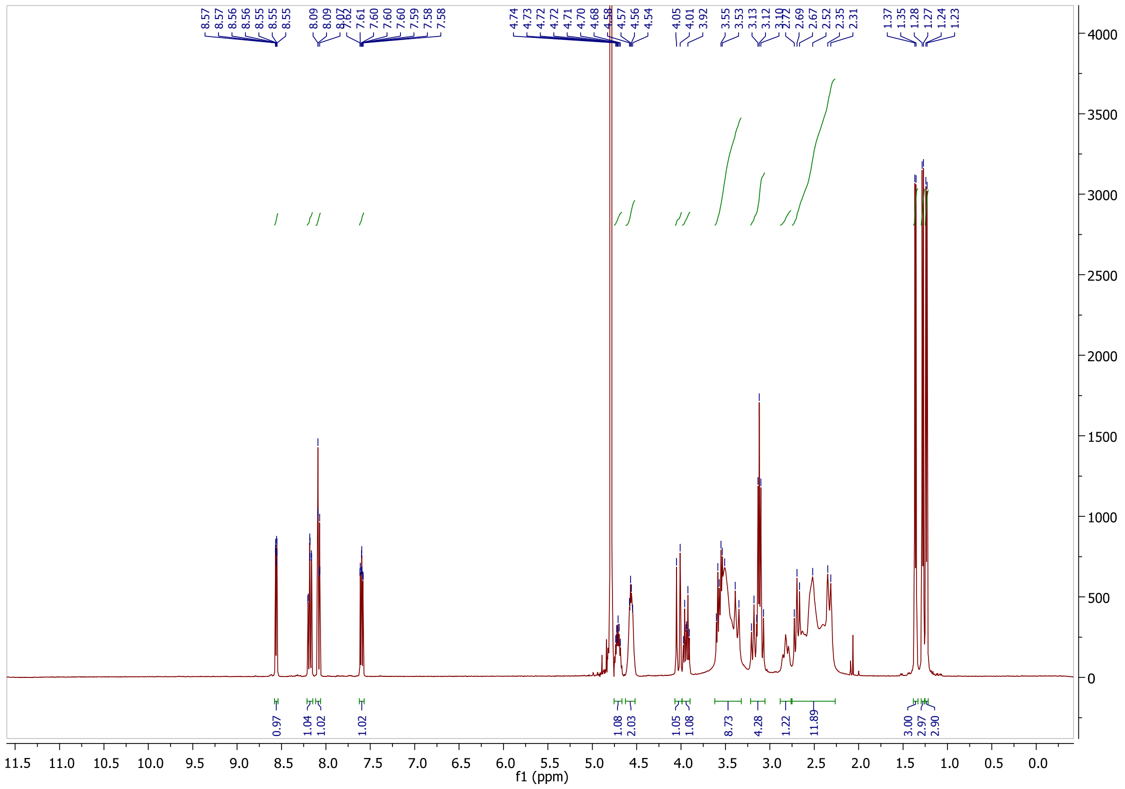

Figure S21. ^{1}H and ^{13}C NMR spectra of **4**.

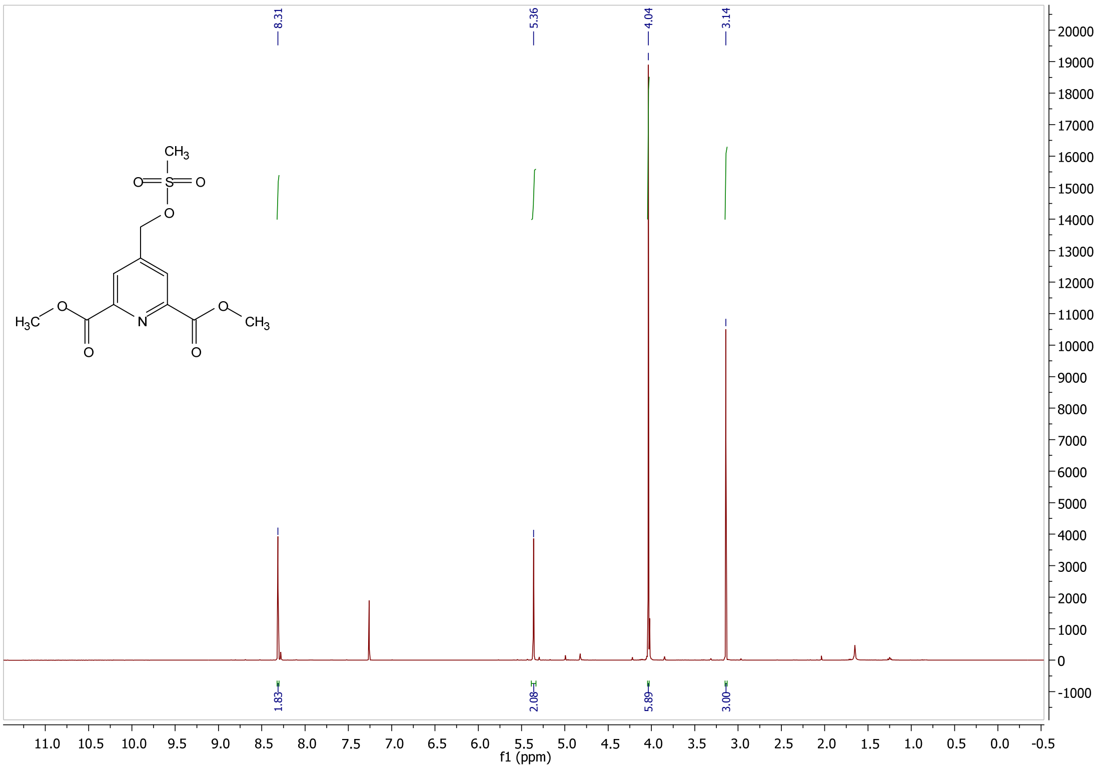

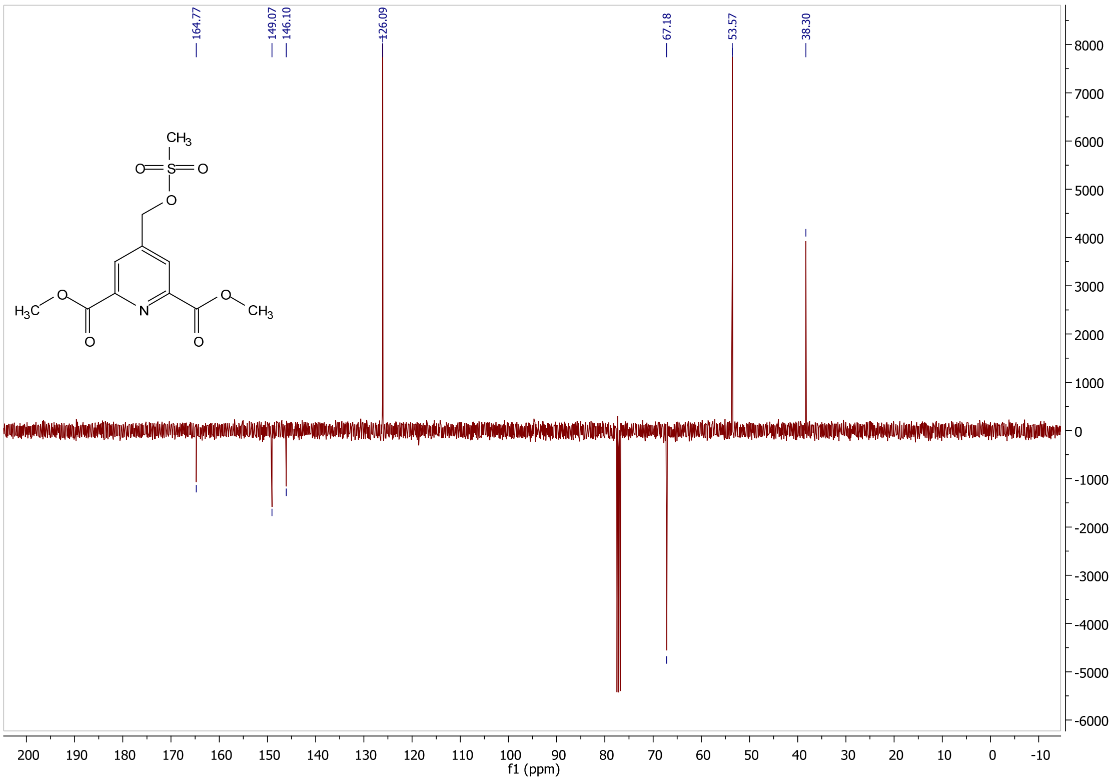

Figure S22. ^{1}H and ^{13}C NMR spectra of **5**.

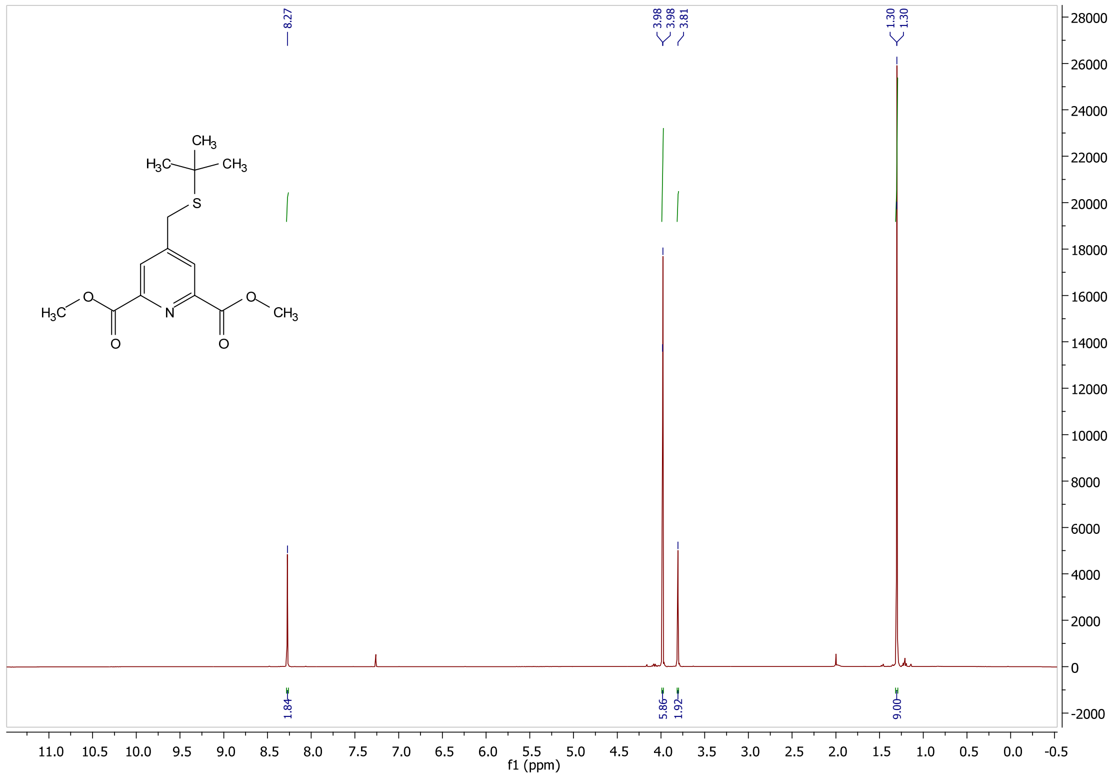

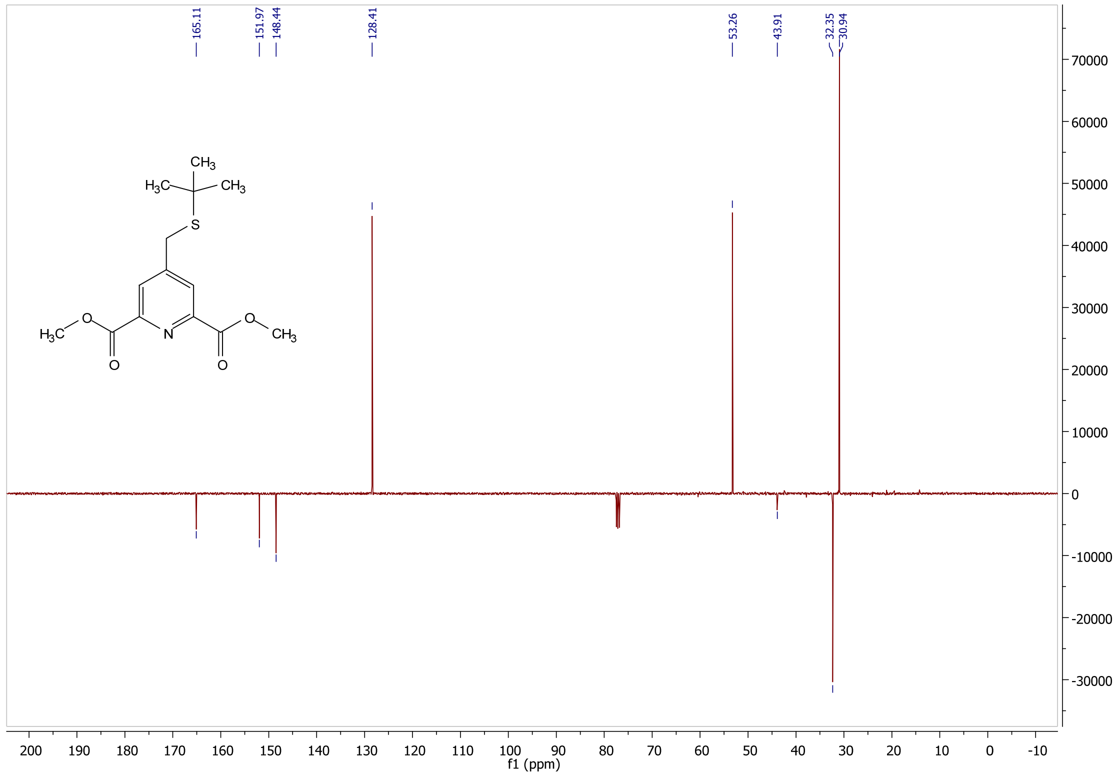

Figure S23. ^{1}H and ^{13}C NMR spectra of **6**.

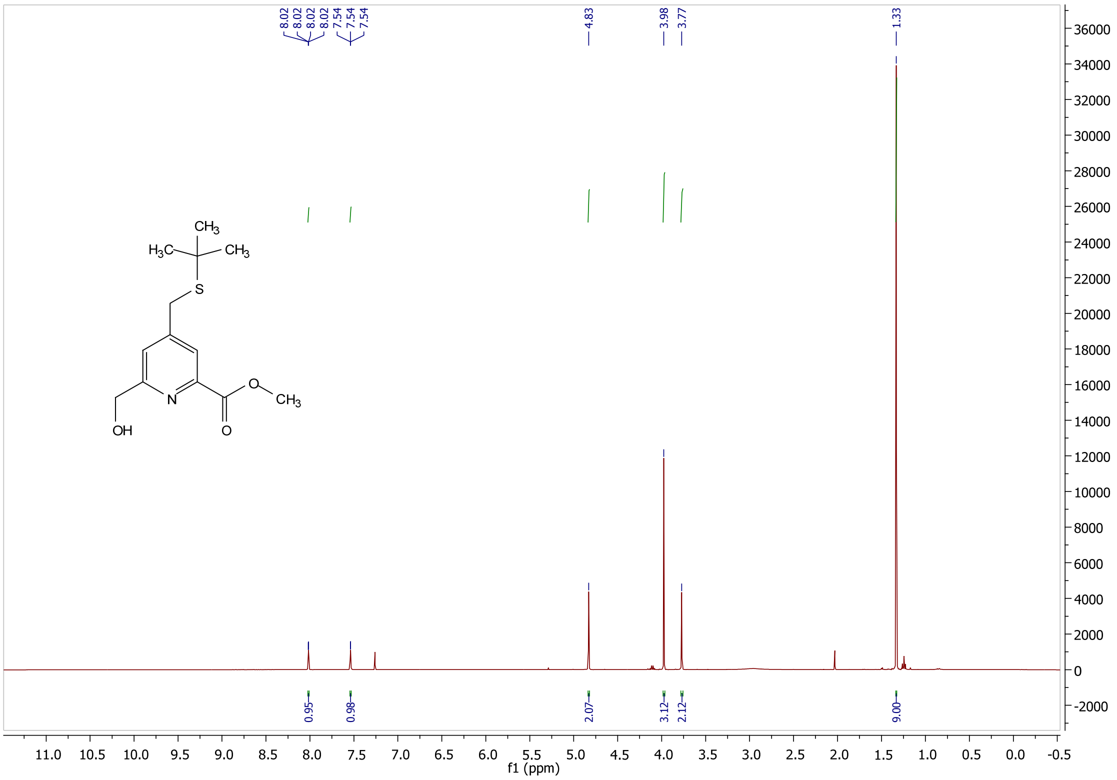

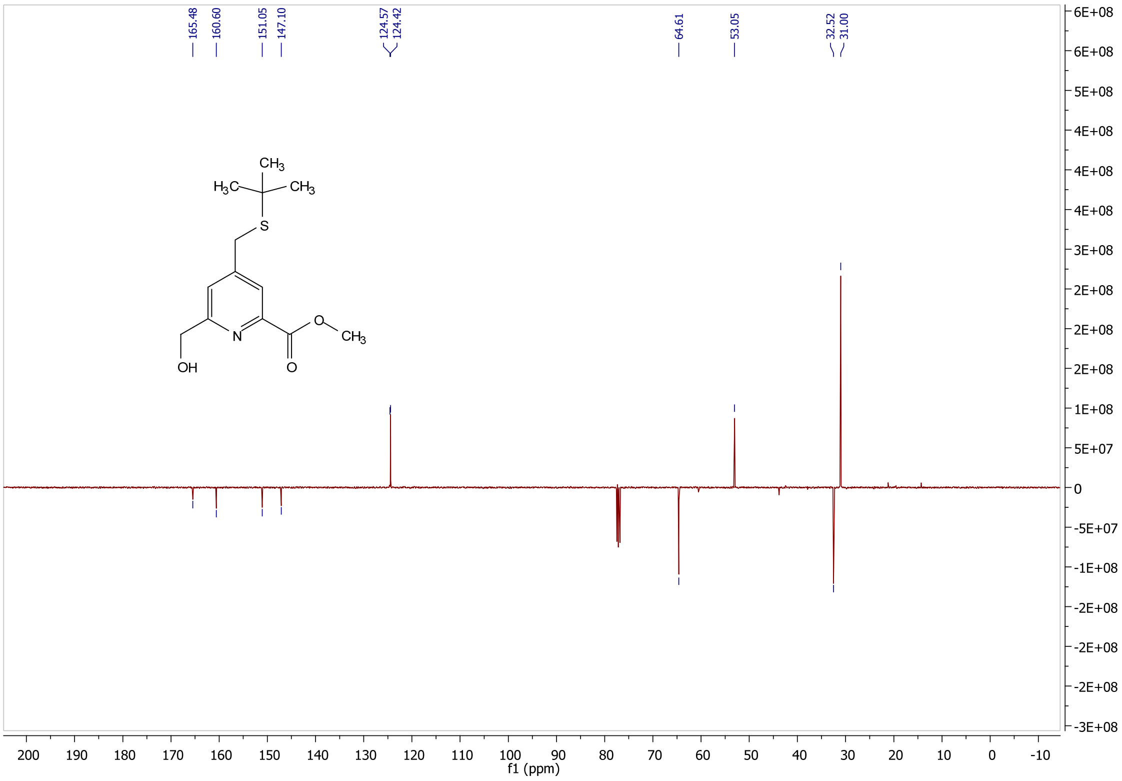

Figure S24. ^{1}H and ^{13}C NMR spectra of **7**.

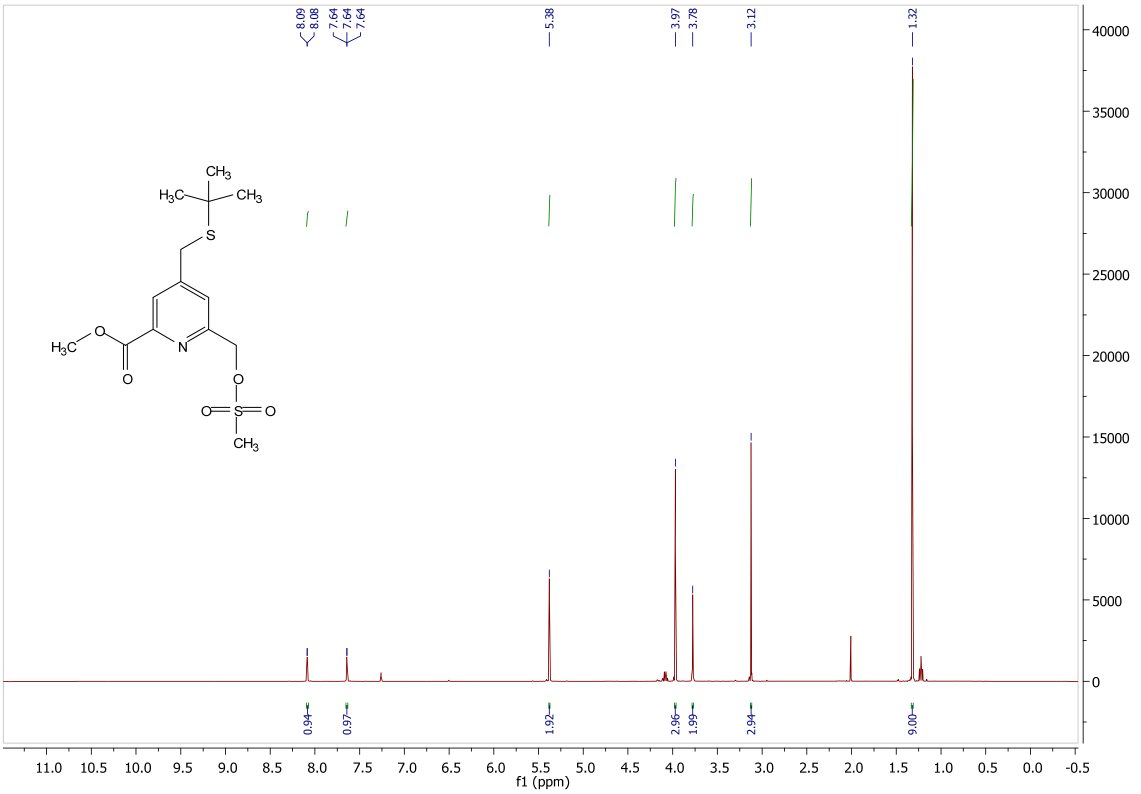

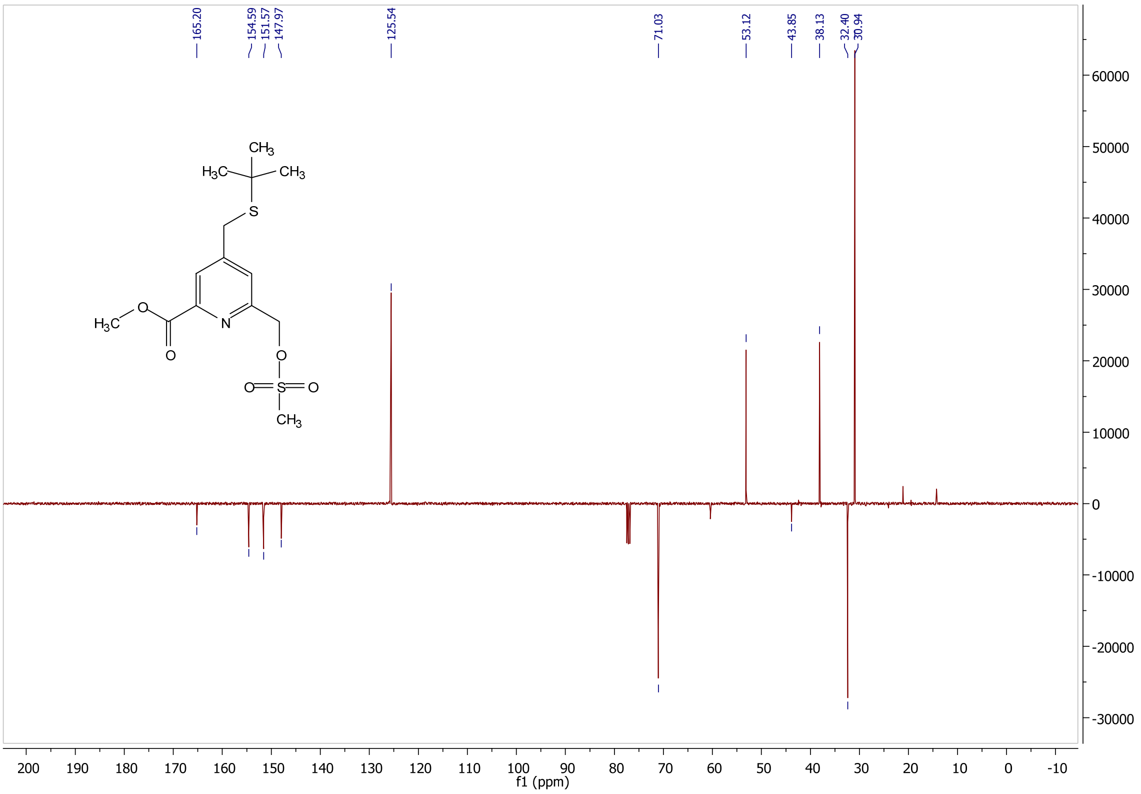

Figure S25. ^{1}H and ^{13}C NMR spectra of **8**.

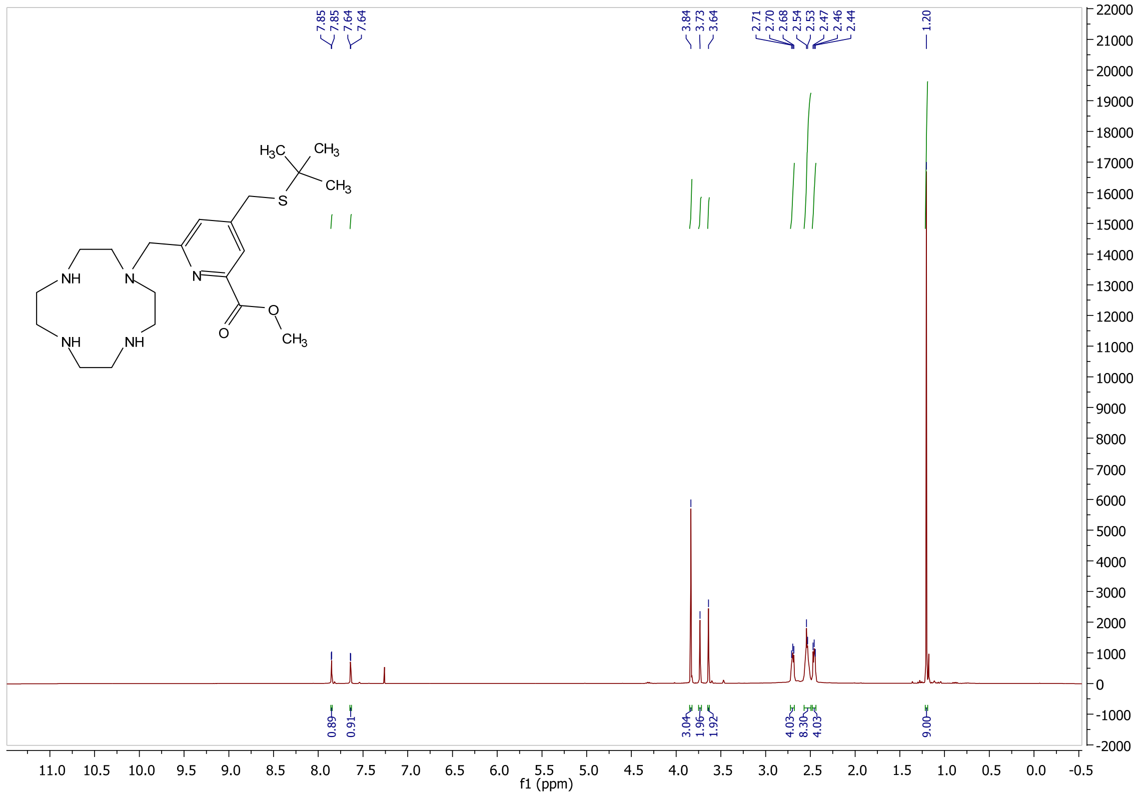

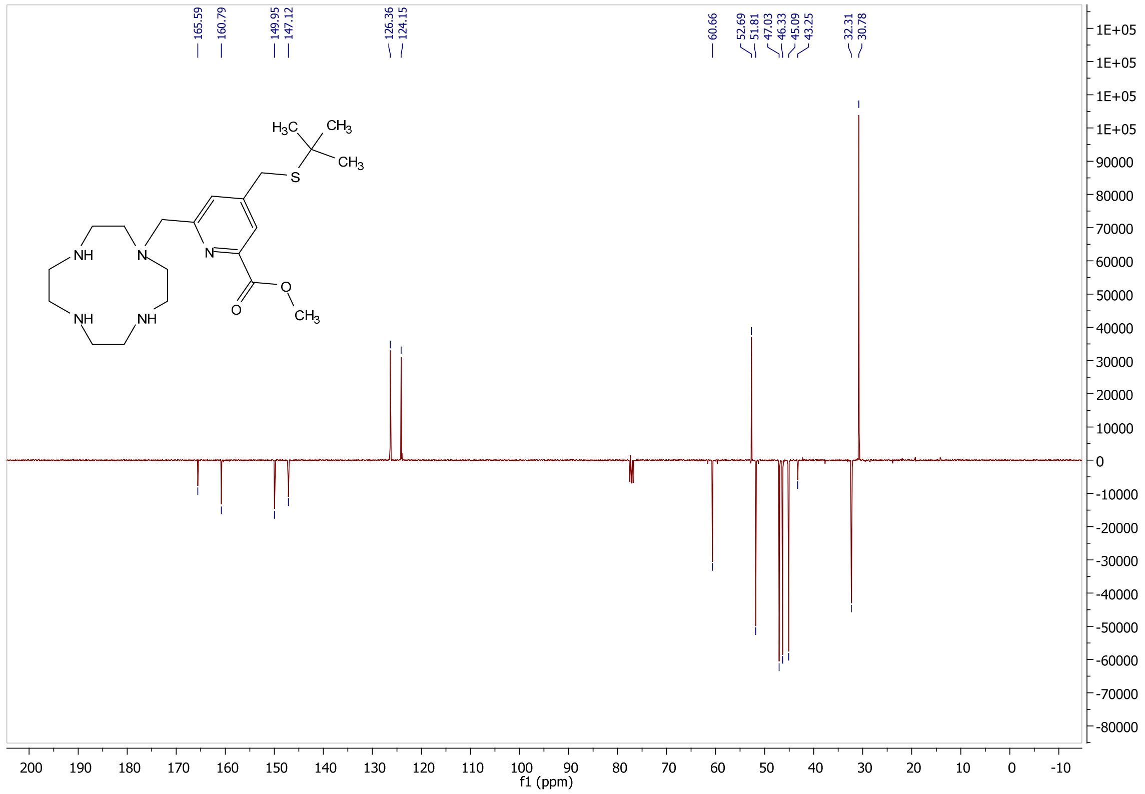

Figure S26. ^{1}H and ^{13}C NMR spectra of **9**.

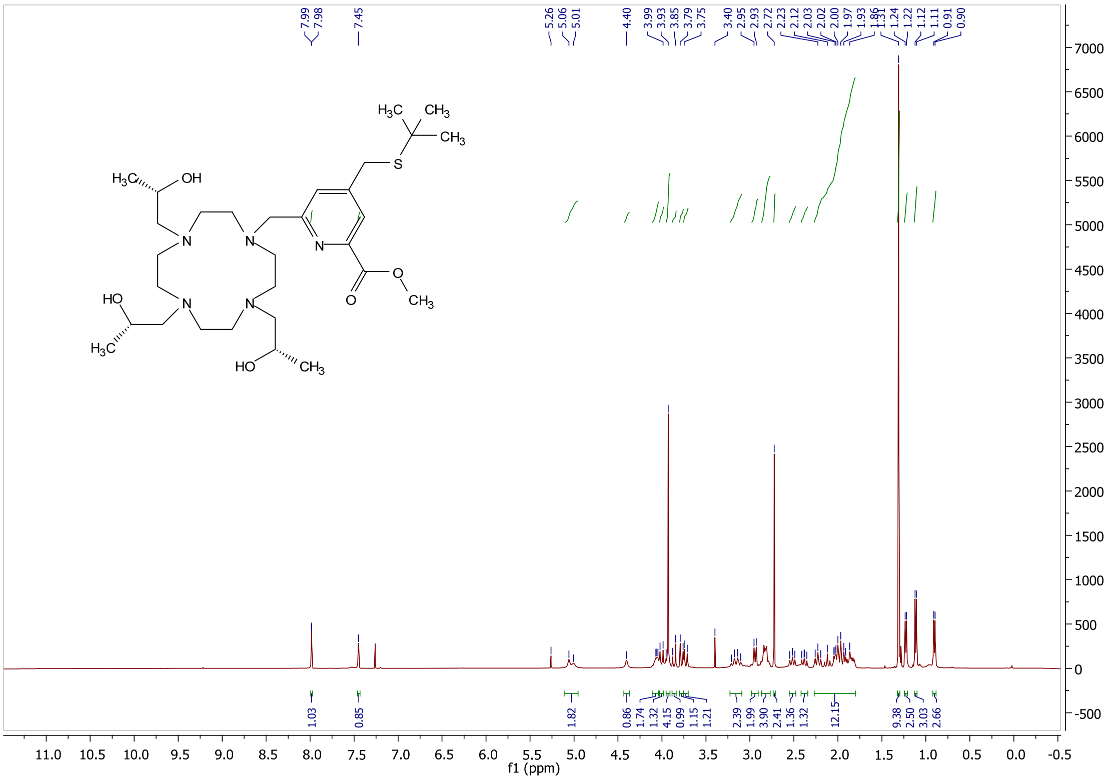

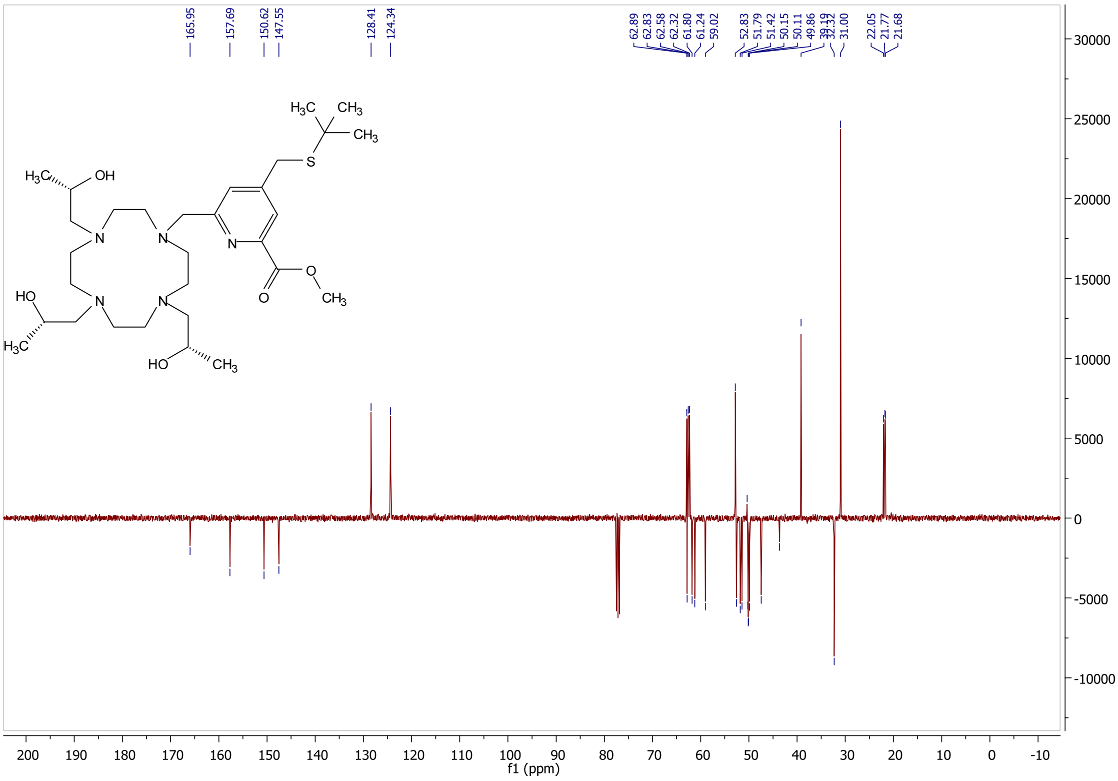

Figure S27. ^{1}H and ^{13}C NMR spectra of **C6**.

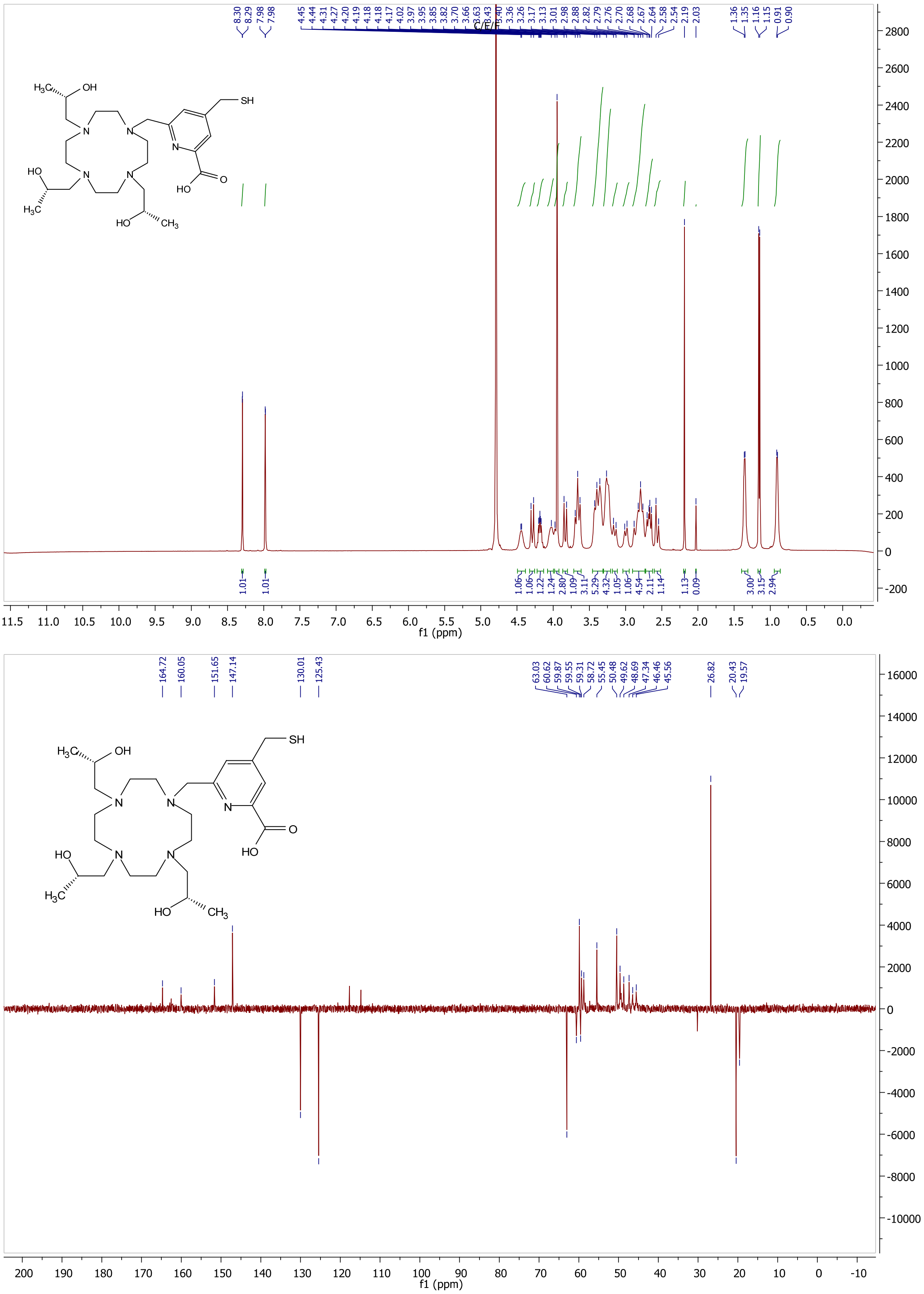

Figure S28. ^{1}H NMR spectrum of **C6-Y^{3+}**.

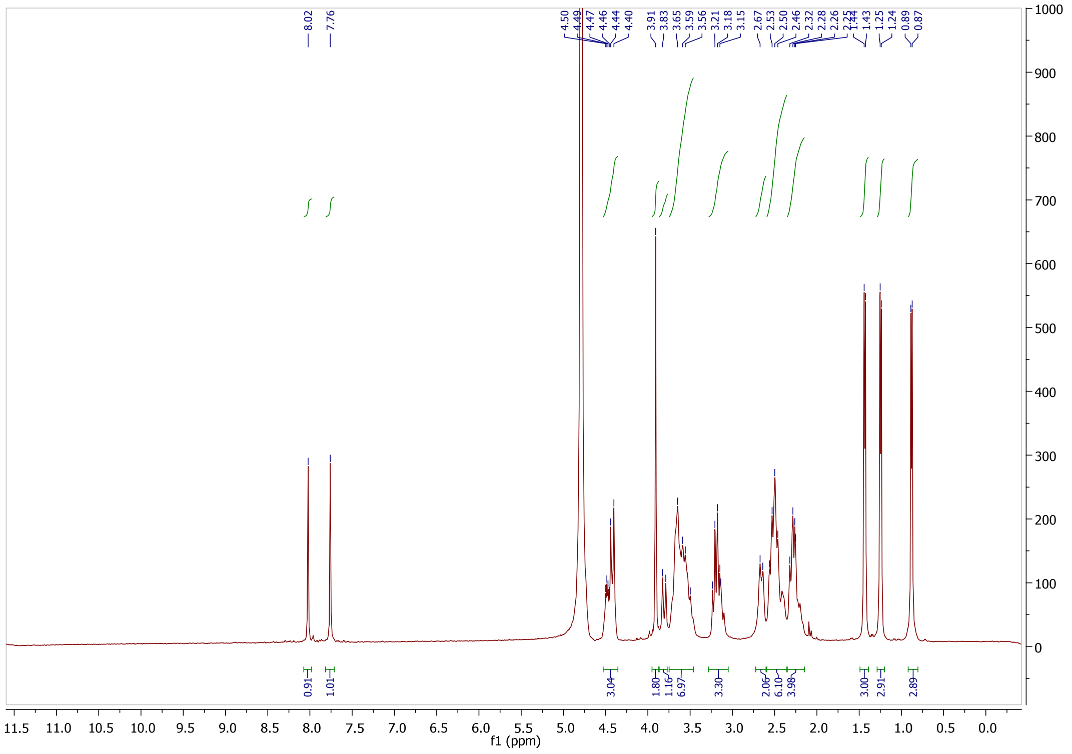

Figure S29. ^{1}H and ^{13}C NMR spectra of **10**.

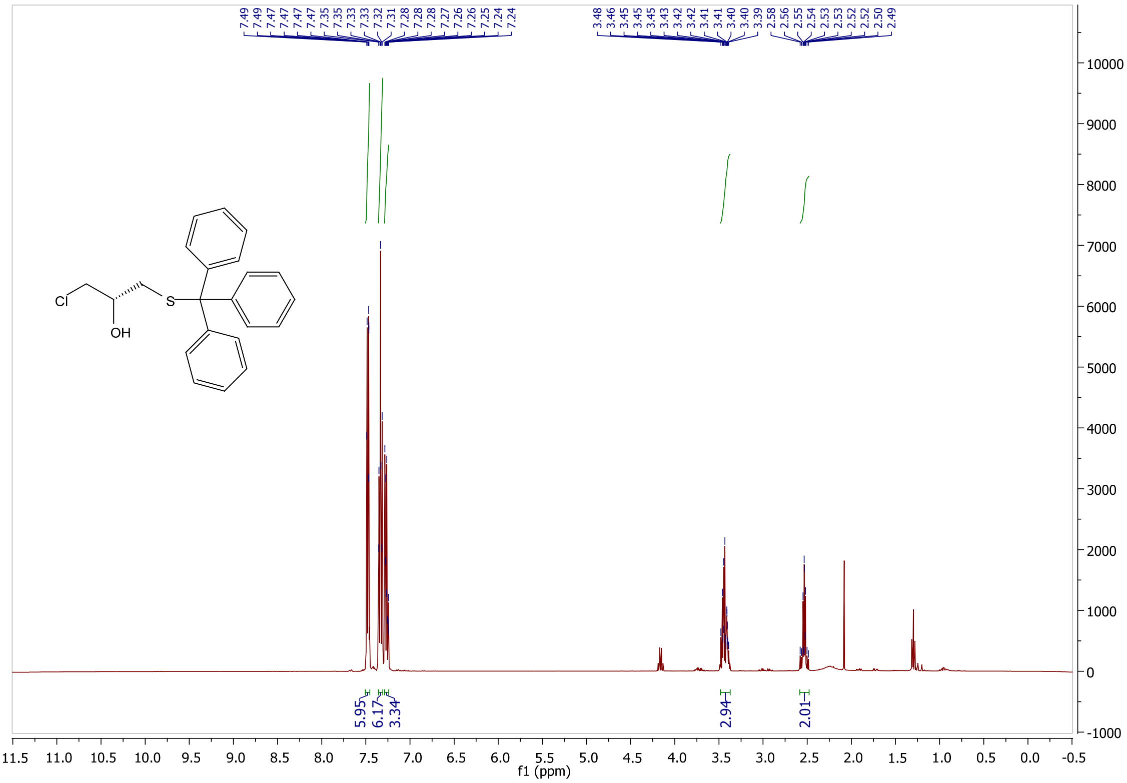

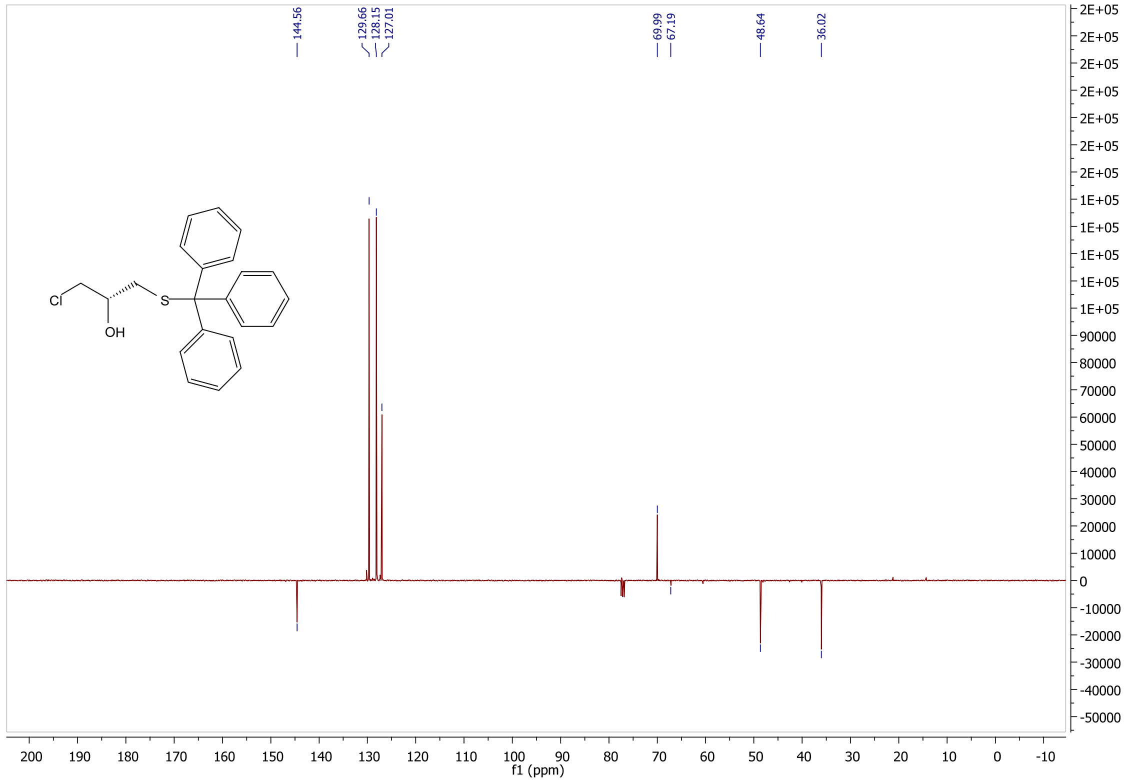

Figure S30. ^{1}H and ^{13}C NMR spectra of **11**.

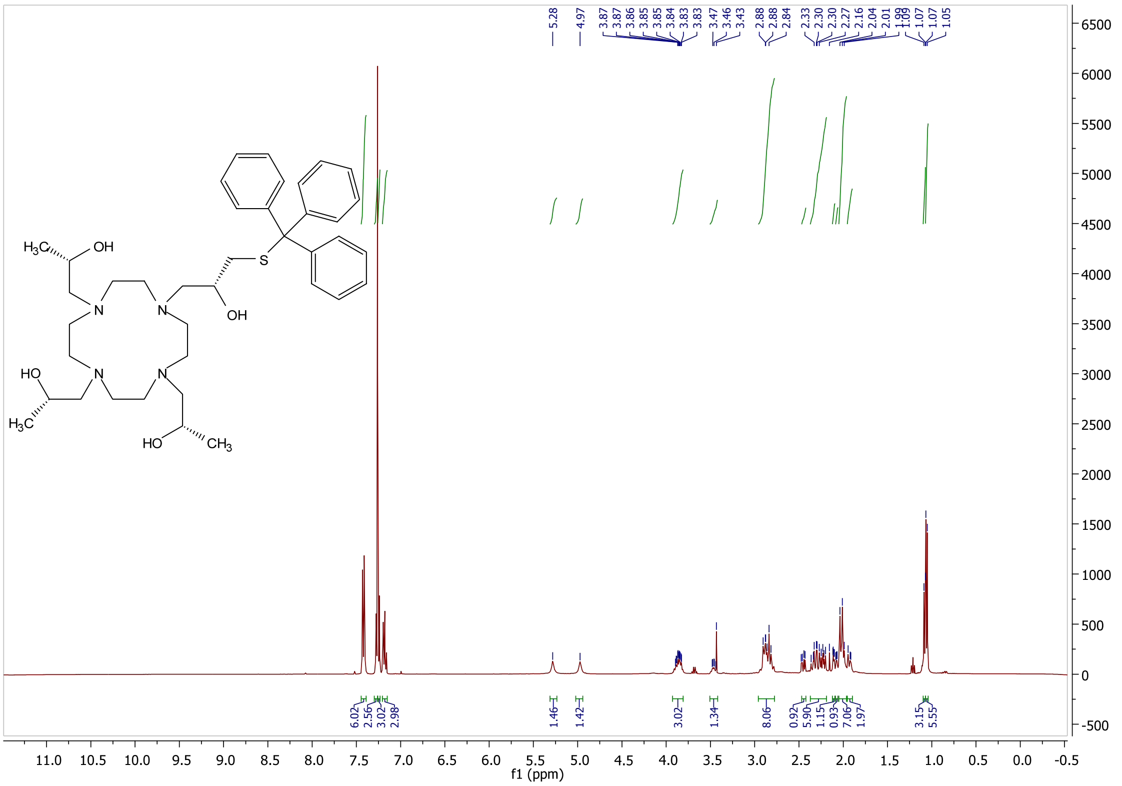

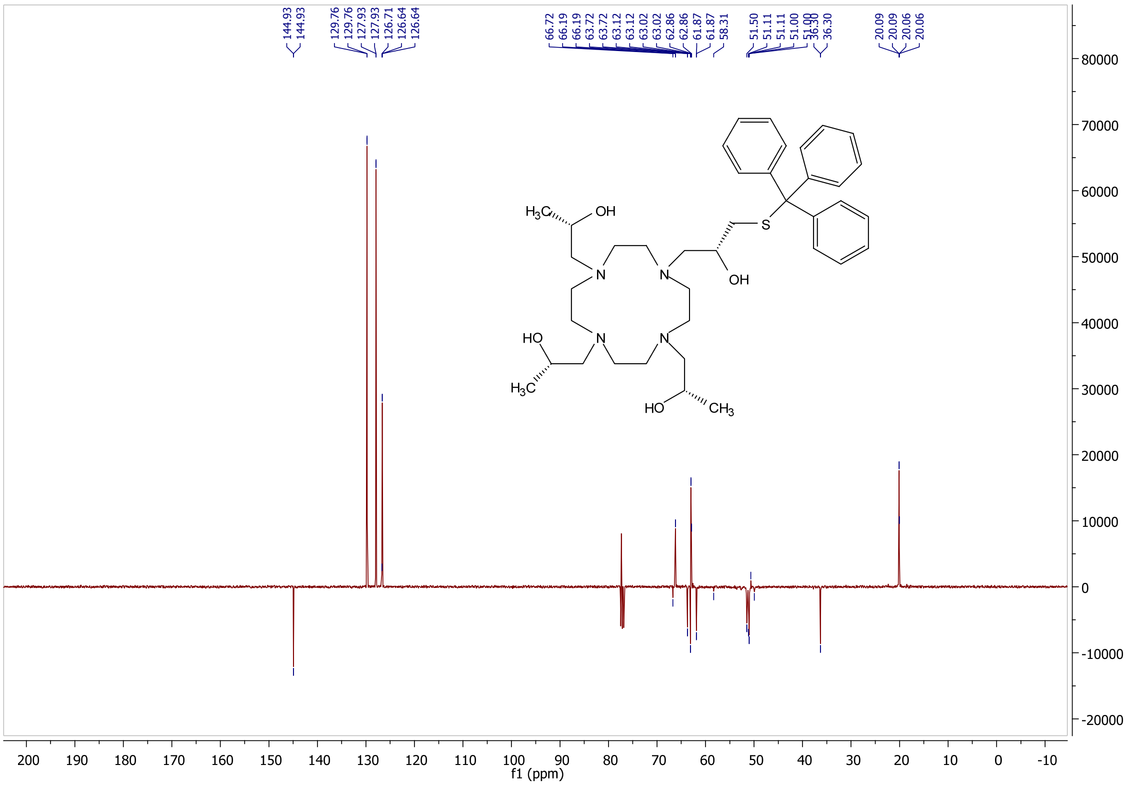

Figure S31. ^{1}H and ^{13}C NMR spectra of **C7**.

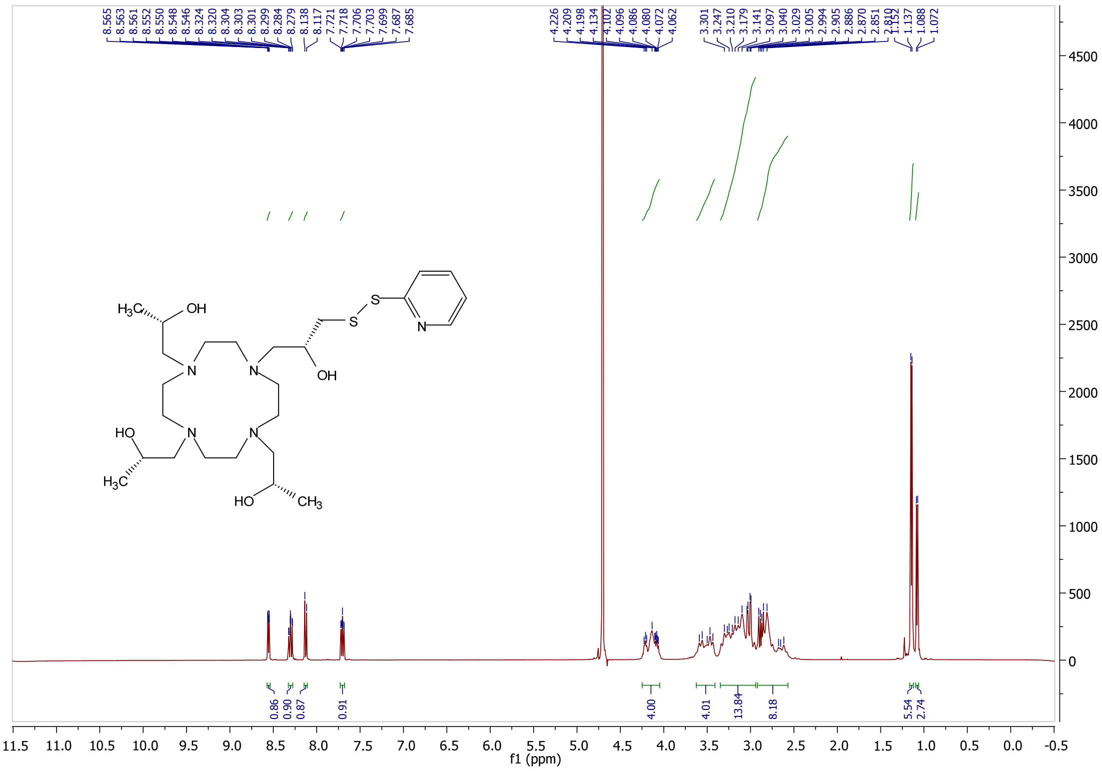

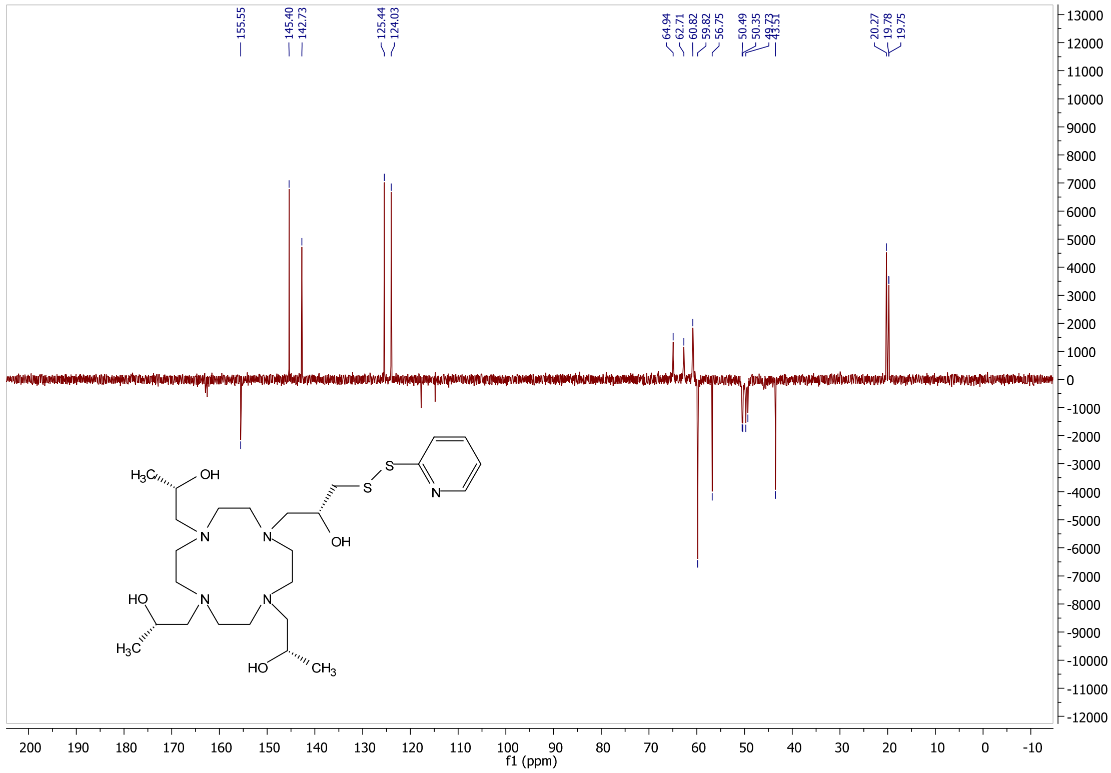

Figure S32. ^{1}H NMR spectrum of **C7-Y^{3+}**.

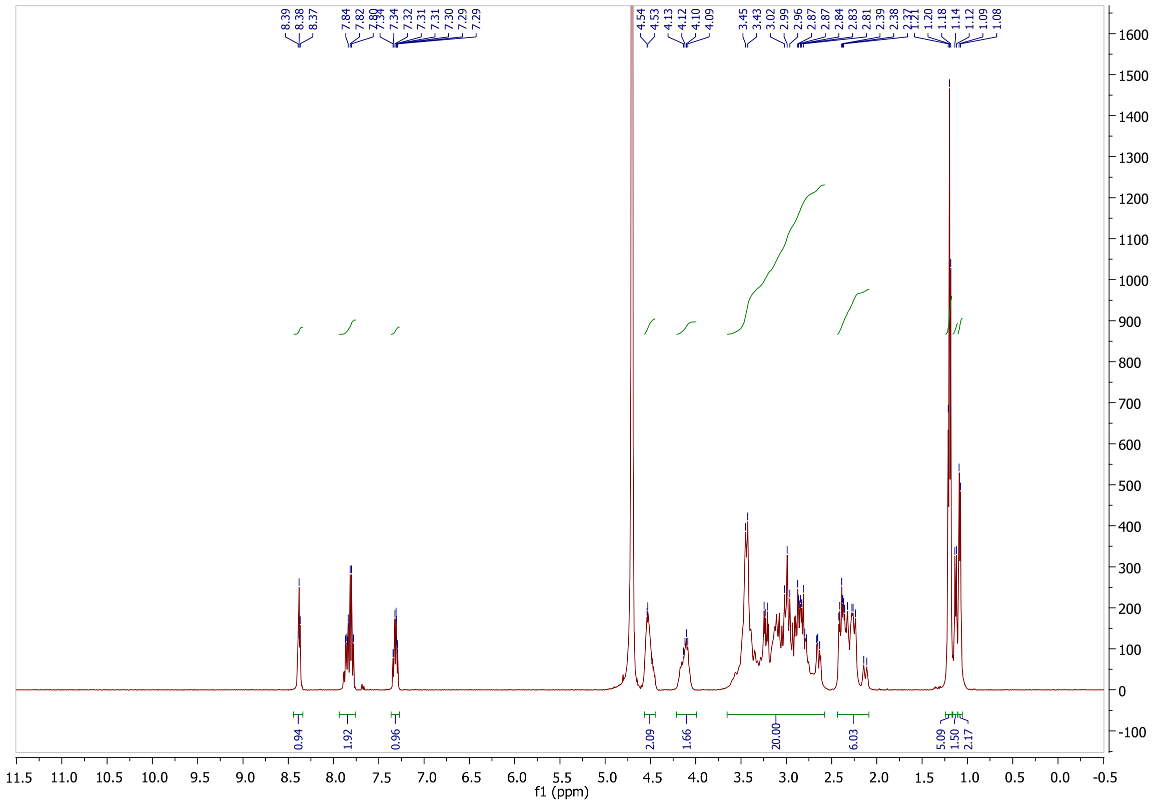

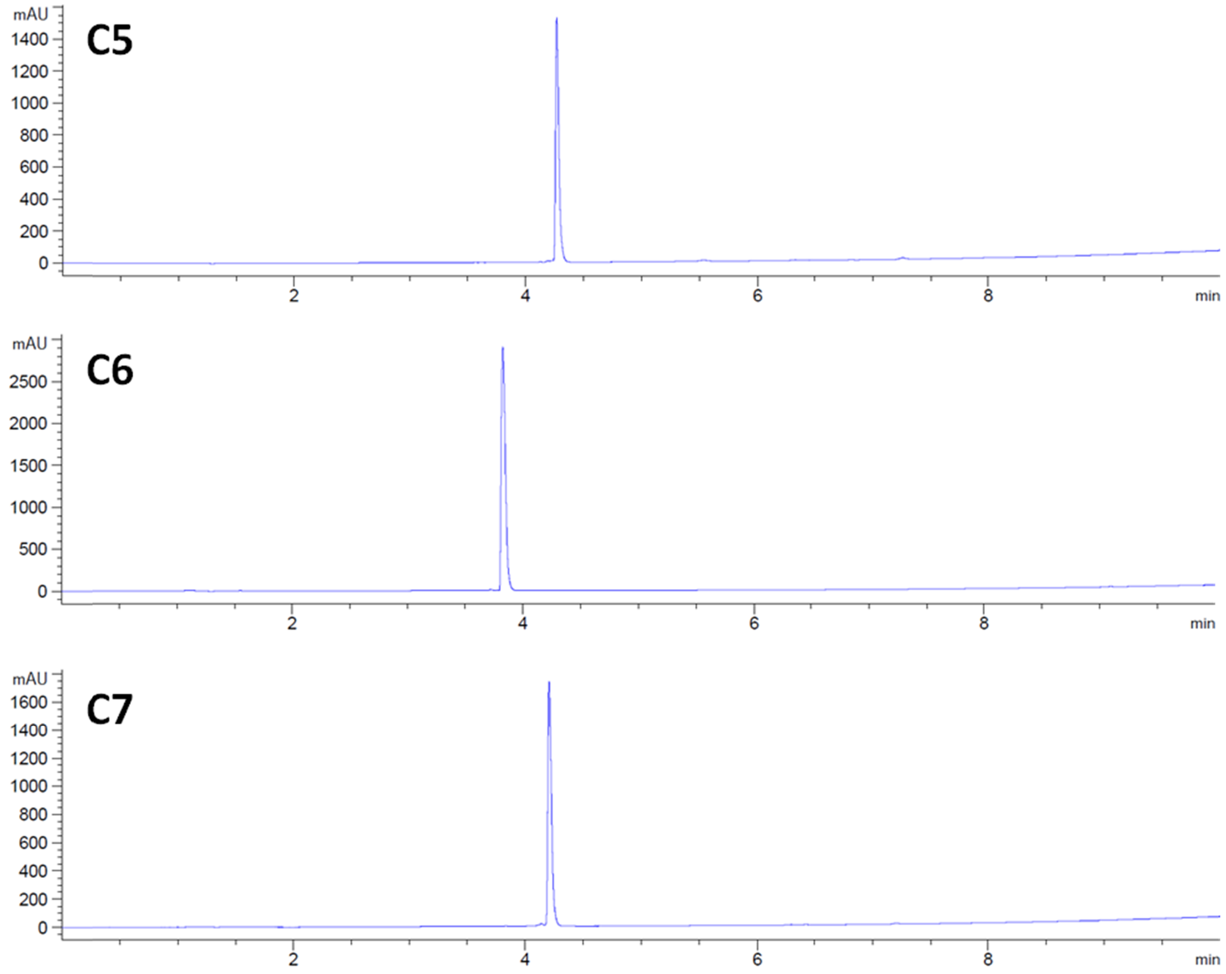

Figure S33. Analytical HPLC traces showing absorption at 254 nm of purified **C5**, **C6** and **C7** tags.

References

1. C. Neylon, S. E. Brown, V. Kralicek, C. S. Miles, C. Love and N. E. Dixon, *Biochemistry*, **2000**, 39, 11989–11999.
2. S. Chhabra, O. Dolezal, B. M. Collins, J. Newman, J. S. Simpson, I. G. Macreadie, R. Fernley, T. S. Peat and J. D. Swarbrick, *PLoS One*, **2012**, 7, e29444.
3. S. Chhabra, J. Newman, T. S. Peat, R. T. Fernley, J. Caine, J. S. Simpson and J. D. Swarbrick, *Acta Crystallogr. Sect. F. Struct. Biol. Cryst. Commun.*, **2010**, 66, 575–578.
4. C. Schmitz, M. J. Stanton-Cook, X.-C. Su, G. Otting and T. Huber, *J. Biomol. NMR*, **2008**, 41, 179–189.
5. A. S. Maltsev, A. Grishaev, J. Roche, M. Zasloff and A. Bax, *J. Am. Chem. Soc.*, **2014**, 136, 3752–3755.
6. T. Gallagher, P. Alexander, P. Bryan and G. Gilliland, *Biochemistry*, **1994**, 33, 4721–4729.
